# Direct, Selective α-Aryloxyalkyl Radical Cyanation and Allylation of Aryl Alkyl Ethers

**DOI:** 10.1021/acs.orglett.4c00392

**Published:** 2024-03-07

**Authors:** Iain Robb, John A. Murphy

**Affiliations:** Department of Pure and Applied Chemistry, 295 Cathedral Street, Glasgow, G1 1XL, Scotland

## Abstract

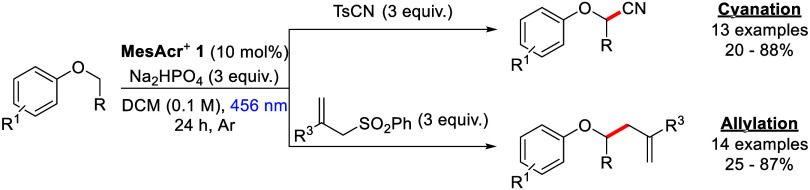

We report the site-selective α-aryloxyalkyl C–H cyanation and allylation of aryl alkyl ethers using an acridinium photocatalyst with phosphate base under LED irradiation (456 nm). Oxidation of the aryl alkyl ether to its corresponding radical cation by the excited stated photocatalyst allowed facile deprotonation of the ArOC(sp^3^)–H bond to afford an α-aryloxyalkyl radical, which was trapped with sulfone substrates, resulting in expulsion of a sulfonyl radical and formation of allylated or cyanated products.

Aryl alkyl ethers occur widely in natural products and pharmaceutical compounds. More than 250 FDA-approved drug molecules^1^ include this feature from melatonin to the complex antifungal agent, micafungin (Figure 1).^2,3^ Due to their abundance and ready availability, aryl alkyl ethers serve as popular targets for functionalization through a variety of chemical transformations.^4,5^

**Figure 1:**
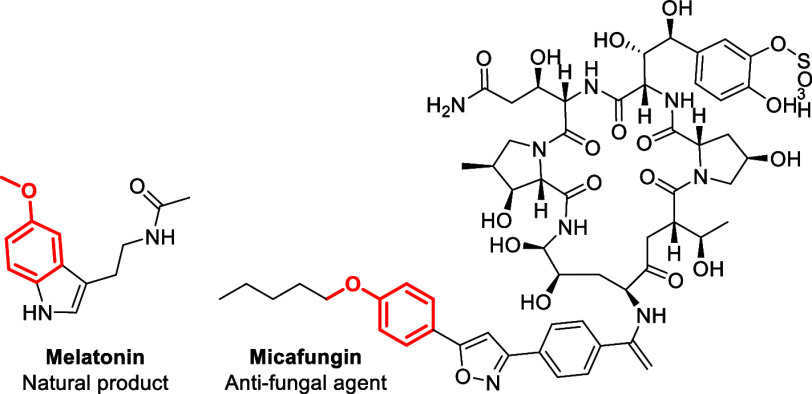
Examples of aryl alkyl ethers.

In recent years photoredox catalysis has become very important,^6,7^ allowing access to highly reactive organic species which would otherwise be unavailable under typical thermal conditions. Currently, great efforts are being made to move away from traditional ruthenium- and iridium-based photoredox catalysts to more environmentally sustainable and economically viable organic photoredox catalysts.^7,8^

Among the most effective organic photoredox catalysts are acridinium salts.^9,10^ Catalyst **1** (Figure 2) is very efficient in the direct oxidation of aryl alkyl ethers to their corresponding radical cations. These radical cations can then facilitate functionalization around the arene ring of the substrate either through direct C–H functionalization^7,10^ or by cation-radical-accelerated nucleophilic aromatic substitution (CRA-S_N_Ar).^8,11^

**Figure 2:**
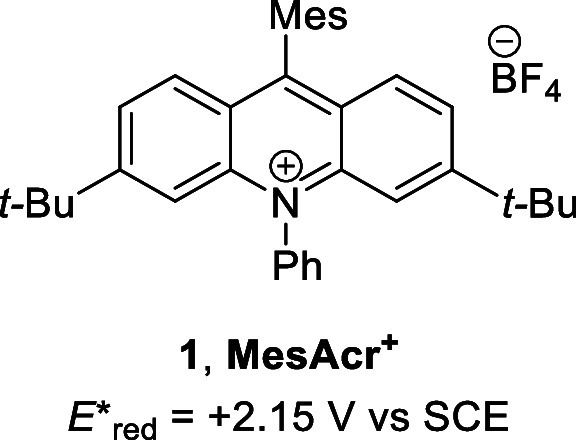
Mesitylacridinium salt **1**.

Recent work within our laboratory has shown that treatment of radical cation **2** with an appropriate base leads to deprotonation at the α-aryloxyalkyl carbon to afford neutral alkyl radical **3** that can then be trapped by suitable Giese acceptors to afford new C–C bonded products **4** (Scheme 1).^12^ Applications following deprotonation of radical cations have also been reported in allylic and benzylic systems, as well as aryl alkyl thioethers.^13,14^

**Scheme 1:**
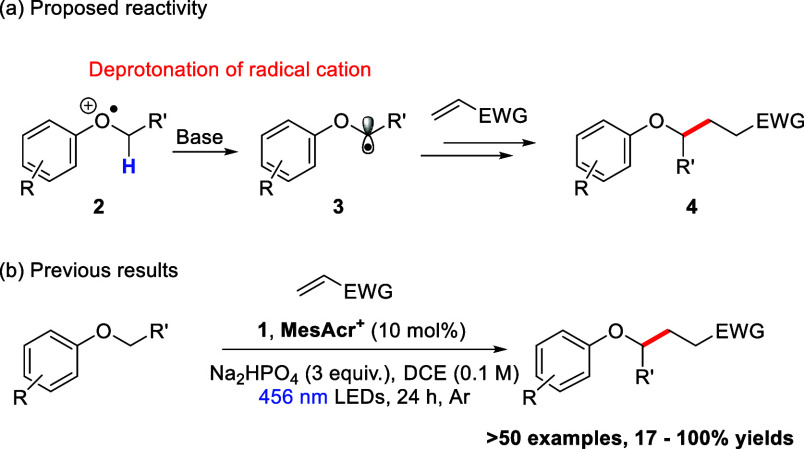
Proposed Formation and Previous Work Regarding α-Aryloxyalkyl Radical **3**

This paper expands this chemistry^12^ to selective radical (i) cyanation and (ii) allylation of aryl alkyl ethers through the use of arenesulfonyl β-leaving groups. Nitriles are widely found in biological and pharmaceutical compounds including the cathepsin C inhibitor, brensocatib, which has also been investigated as a COVID-19 treatment (Figure 3),^15,16^ as well as the anti-inflammatory/antiasthmatic agent, cilomolast, and the natural product renieramycin M.^16,17^ Nitriles are also flexible functional handles in synthesis.^18^ Besides nitriles, installation of an allyl group in a molecule can also allow for diverse further functionalization.^19−21^ Moreover, through variation of the substituents on the starting allylic sulfone substrate, radical allylation can enable direct incorporation of new functional groups, which can be important in the coupling and modification of pharmaceutically relevant compounds.^22^

**Figure 3:**
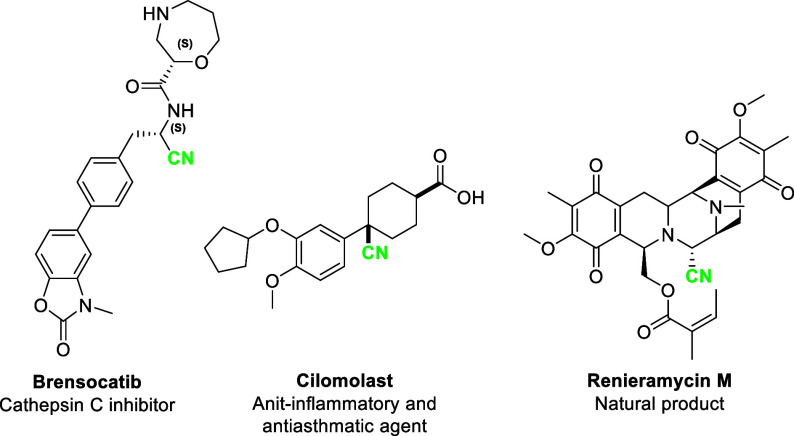
Pharmaceutically relevant compounds containing a nitrile group.

Recent reports of the photochemically mediated radical cyanation/allylation of α-oxy C(sp^3^) carbons either proceed primarily via hydrogen atom transfer (HAT) or through transformation of a functional group (which we will refer to below as a “radical precursor”) present on the molecule into a radical.^19,21,23−30^ In 2011, Inoue et al. published methods for the α-oxyalkyl cyanation of aliphatic ethers using benzophenone under UV light irradiation as an HAT agent and tosyl cyanide (TsCN) as the cyano source, work which they later expanded in 2013.^23,26^ More recently, Hong et al. demonstrated the use of tetrabutylammonium decatungstate (TBADT) as a visible-light-activated HAT catalyst to cyanate C(sp^3^)–H bonds, including aryl alkyl ethers (Scheme 2a).^24^ Moreover, Kamijo allylated aliphatic carbons through a HAT process facilitated by irradiating the aryl ketone 5,7,12,14-pentacenetetrone.^19^ Also, a 1,5-HAT reaction enabled by arylcarboxyl radicals resulting from visible light irradiation of a donor–acceptor complex with a Hantzsch ester designed by the Chen group allowed the allylation of several α-oxy C(sp^3^)–H bonds, as shown with aryl alkyl ether **7** (Scheme 2b).^30^

**Scheme 2:**
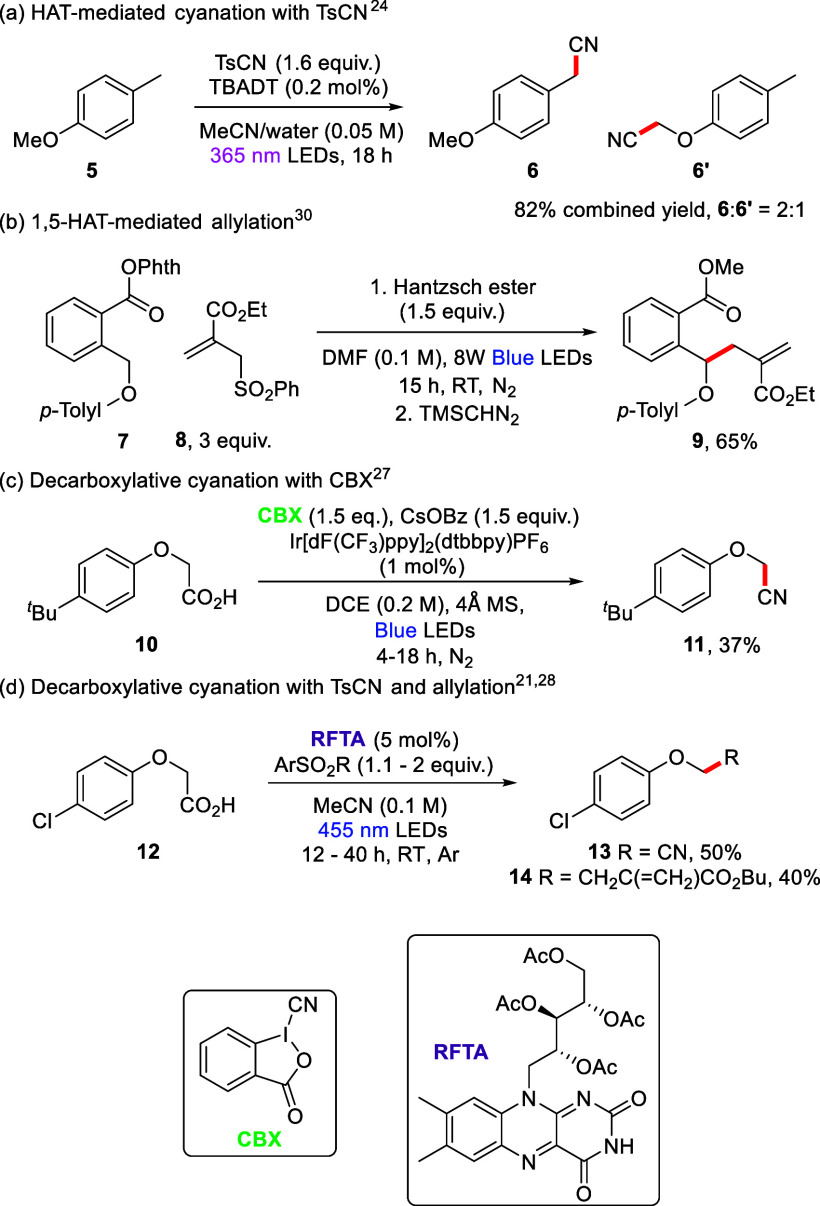
Previous Photochemically Enabled Cyanation/Allylation of Aryl Alkyl Ethers

The cyanation and allylation of aliphatic carbons via alkylboranes have been championed by Renaud et al,^31,32^ while, in 2016, the Molander group reported both C(sp^3^)–H cyanations and allylations employing potassium alkyltrifluoroborates as radical precursors, which afforded the corresponding alkyl radicals following oxidation by either eosin-Y or an acridinium photocatalyst.^25^ Meanwhile, Xu et al. independently published C(sp^3^)–H cyanation also using potassium alkyltrifluoroborates but with a ruthenium photocatalyst.^33^ Another popular radical precursor group used in both cyanation and allylation reactions is COOH/R, which can decarboxylate via different visible-light-mediated pathways to produce alkyl radicals.^27,28^ Waser used an iridium photocatalyst along with cyanobenziodoxolone (CBX) to give decarboxylative cyanation of aryl alkyl ethers (Scheme 2c).^27^ Gonzalez-Gomez et al. have also reported decarboxylative cyanation and allylation of aryl alkyl ethers, this time using riboflavin tetraacetate (RFTA) as both the photocatalyst and base with visible light irradiation (Scheme 2d).^21,28^

Despite the impressive progress made in the visible-light-mediated cyanation of C(sp^3^) carbons, issues with the techniques previously discussed still remain. The HAT methodologies tend to suffer from regioselectivity problems due to their dependence on bond dissociation energies (BDEs) which can lead to multiple active C(sp^3^)–H bonds in the same molecule.^12,19,23,24,29^ In the case of radical precursors, if the desired substrate is not commercially available with the required precursor group already attached (e.g., carboxylic acids),^21,27,28^ then at least one extra step is required in the synthetic pathway to reach the desired substrate.^30,33^ Therefore, this work addresses these drawbacks through direct and selective α-aryloxyalkyl C–H cyanation and allylation of aryl alkyl ethers (Scheme 3).

**Scheme 3:**
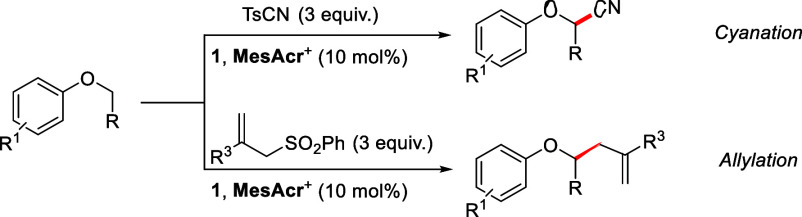
The Proposed Cyanation/Allylation of Aryl Alkyl Ethers

The best protocol for the cyanation of aryl alkyl ethers used DCM as solvent (see Supporting Information, Table S1).^12^ The optimized conditions included **1**, **MesAcr**^**+**^ (10 mol %) with TsCN (3 equiv) and Na_2_HPO_4_ (3 equiv) in DCM (0.1 M) under argon and irradiation with blue light (Kessil Lamp, 456 nm) for 24 h (Scheme 4). Under this procedure, both primary and secondary aryl alkyl ethers reacted in high yield to afford the corresponding cyanohydrin ethers (**15** and **16**). Increasing the alkyl chain length made little difference to the reaction outcome, and product **18** was recovered in 79% yield. A tertiary aryl alkyl ether, isoproproxybenzene, also gave the expected cyanohydrin ether containing a tetrasubstituted carbon (**17**). The lower yield with this substrate may be due to the steric hindrance afforded to the resultant α-aryloxyalkyl radical by the adjacent methyl groups.

**Scheme 4:**
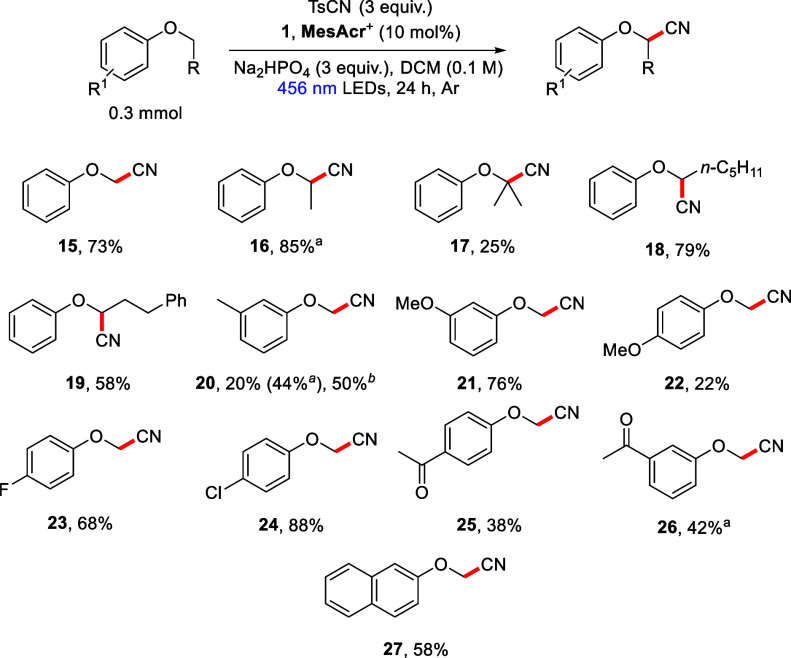
Substrate Scope of the α-Aryloxyalkyl Cyanation^,^ NMR yield determined using 1,1,2,2-tetrachloroethane as internal standard. 390 nm LEDs used.

(3-Phenoxypropyl)benzene underwent selective cyanation at the α-aryloxyalkyl position, giving **19** in moderate yield. Given that under HAT conditions this substrate has been shown to undergo functionalization at all three C(sp^3^) carbons, this result highlights the superior regioselectivity of this procedure.^12,34^ 3-Methylanisole was monocyanated exclusively at the α-aryloxyalkyl carbon to give cyanohydrin ether **20** with no benzylic cyanation observed (this product was volatile: NMR yield, 44%; isolated yield, 20%). This regioselectivity contrasts with previous HAT protocols using 4-methylanisole where benzylic cyanation was favored.^23,24,26^ Additionally, reaction of 3-methoxyanisole delivered the expected monocyanated product **21** (76%) with only 7% NMR yield of the dicyanated adduct detected. 4-Methoxyanisole, however, gave only the desired monocyanated compound **22**, albeit in lower yield. Electron-deficient aryl ethers were also successfully cyanated in moderate to high yield (**23**–**26**), with electron-withdrawing groups tolerated at both the 3- and 4-position of the ring (**26** and **25**, respectively). The halogen atoms present in **23** and **24** provide functional handles for the construction of larger, more complex structures. Finally, 2-methoxynaphthalene was also a successful substrate under these conditions, producing adduct **27** (58%).

The same reaction conditions were also applied to the allylation of a range of aryl alkyl ethers with various allylic sulfones (Scheme 5). Again, primary, secondary, and tertiary aryl alkyl ethers were all successfully allylated to give the corresponding products in low to high yields (**28**–**31**) with the formation of tetrasubstituted carbon centers in both **30** and **31**. The impact of altering the length and functionalization of the alkyl chain of the aryl alkyl ethers was next investigated (**32**–**35**). (Hexyloxy)benzene underwent radical allylation to afford compound **32** (59%). Mirroring the cyanation result, (3-phenoxypropyl)benzene was selectively allylated at the α-aryloxyalkyl site, producing the single regioisomer **33** in modest yield. Benzyl phenyl ether was also smoothly allylated to give compound **34** (65%). Allylation product **35**, containing a protected amine moiety, was pleasingly recovered (73%) and, following deprotection, could offer a versatile functional handle for further manipulations. Different electron-donating alkyl groups were tolerated around the aryl ring, affording products **36** and **37** in low yield. Furthermore, the reaction to yield compound **36** offered no benzylic allylation, again highlighting the selectivity of this radical reaction. However, over-reaction of compound **37** to give adduct **38** was observed, with **38** being isolated (15%). When this is taken into consideration, it can be seen that compound **37** must have been formed in at least 26% yield over the course of the reaction. Electron-deficient halo-substituted anisole substrates were also allylated, providing **39** and **40** in moderate yields, each with a convenient handle for further modifications.

**Scheme 5:**
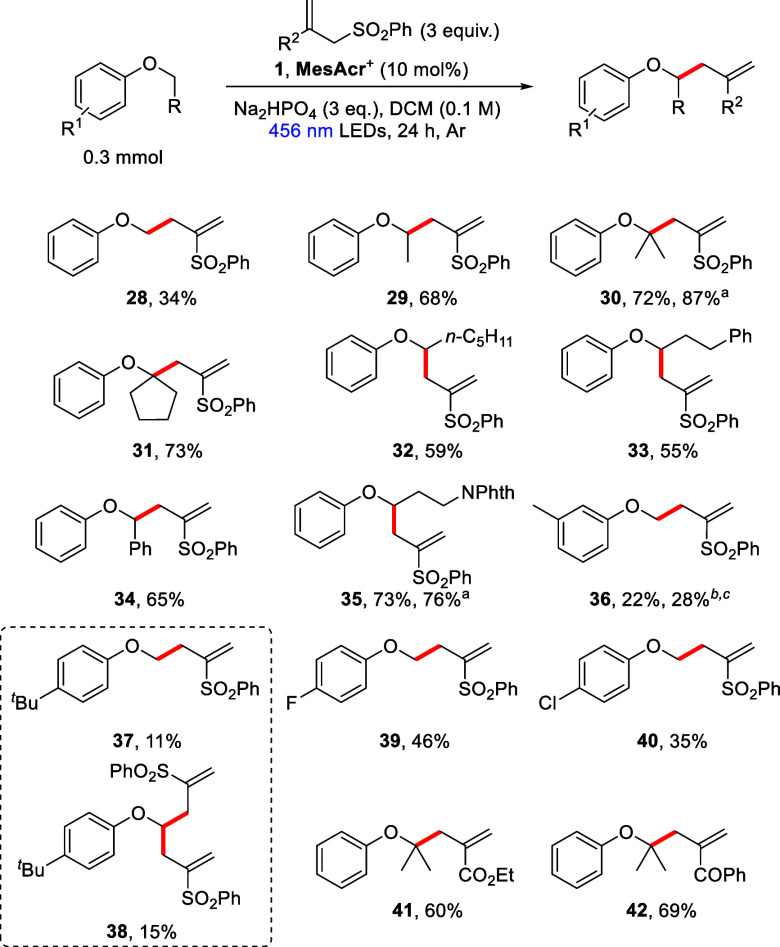
Substrate Scope of the α-Aryloxyalkyl Allylation^,^^,^ The reaction was run on a 1.0 mmol scale. NMR yield determined using 1,1,2,2-tetrachloroethane as internal standard. 390 nm LEDs used.

Overall, when looking at the aryl alkyl ether scope for this reaction, both tertiary (**30** and **31**) and secondary (**29** and **32**–**35**) ether substrates outperformed the primary anisole derivatives (**28**, **36**, **37**, **39**, and **40**). This may be due to the lower nucleophilicity of the resultant primary radical making addition to the allylic sulfone less efficient.^35^ Moreover, despite only a single diallylated product **38** being isolated [when 4-*tert*-butyl-anisole was used], characteristic minor peaks of the corresponding diallylated products were observed in the crude ^1^H NMR spectra of the reactions involving anisole and its other substituted derivatives. This means that this may also contribute to the overall lower yields obtained from primary ethers when compared with secondary and tertiary substrates.

Given our success in improving product yields in our previous work using 390 nm purple light, this was again investigated in this work using 3-methylanisole.^12^ However, the yields of products **20** and **36** were only slightly increased (Scheme 4 and Scheme 5, respectively).

Further studies regarding the nature of the allylic sulfone reagent revealed that both an ester and a ketone group, in place of the phenyl sulfone moiety, also allowed smooth allylation, affording compounds **41** and **42** in high yield (Scheme 5). These results indicate that this reaction is not limited to 2,3-bis(phenylsulfonyl)-1-propene and that another level of variability is available in the substituents of the allylic substrate.

To demonstrate the practicality of this allylation protocol, the reactions concerning two of the highest yielding ether substrates were scaled up (from 0.3 to 1.0 mmol), and pleasingly, in both cases, an increase in yield was observed giving **30** and **35** (87% and 76%, respectively) (Scheme 5). These products were then further functionalized under the same reaction conditions, making use of the newly installed alkene moiety to facilitate another C(sp^3^)–C(sp^3^) coupling to a different aryl alkyl ether (Scheme 6). In this way, compound **30** was successfully coupled to 4-fluoroaisole (**43**) to give sulfone **44** (27%), and product **35** was reacted with phenetole (**45**) to yield adduct **46** (77%) as a complex mixture of diastereomers (see the Supporting Information).

**Scheme 6:**
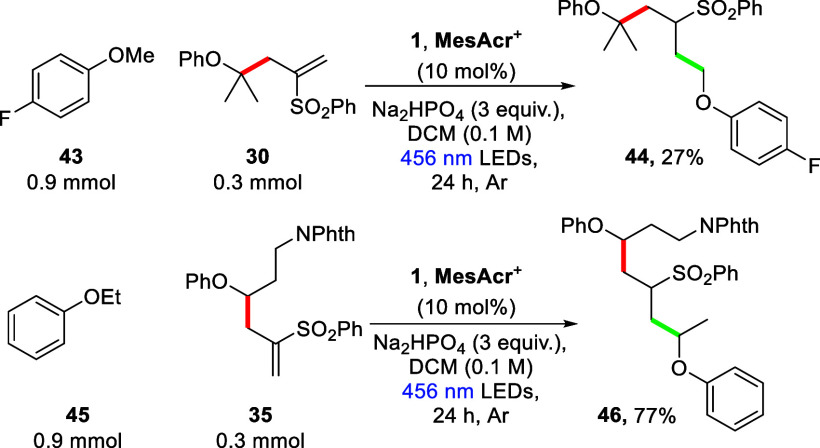
Further Functionalization of Previously Allylated Products under the Developed Reaction Conditions

A proposed reaction pathway, based on previous mechanistic investigations,^12^ is shown in Scheme 7.^21,28^ The reaction begins with the excitation of acridinium salt **1**, through visible light irradiation, to give **[MesAcr**^**+**^]*. This excited state then oxidizes aryl alkyl ether **47** to afford the corresponding radical cation **48** and the reduced form of the photocatalyst, **MesAcr**^•^. Deprotonation of **48** then produces the nucleophilic α-aryloxyalkyl radical **49**, which undergoes a radical addition–elimination reaction with the chosen ArSO_2_R reagent forming a new C–C bond to yield the desired product **50** and the resulting sulfonyl radical (ArSO_2_^•^). The catalytic cycle is then closed through the single electron transfer from **MesAcr**^•^ (*E*_ox_ = −0.56 V vs SCE)^36^ to ArSO_2_^•^ (*E*_red_ = +0.50 V vs SCE),^37^ which regenerates the ground state acridinium salt **1** and produces the sulfinate anion (ArSO_2_^–^).

**Scheme 7:**
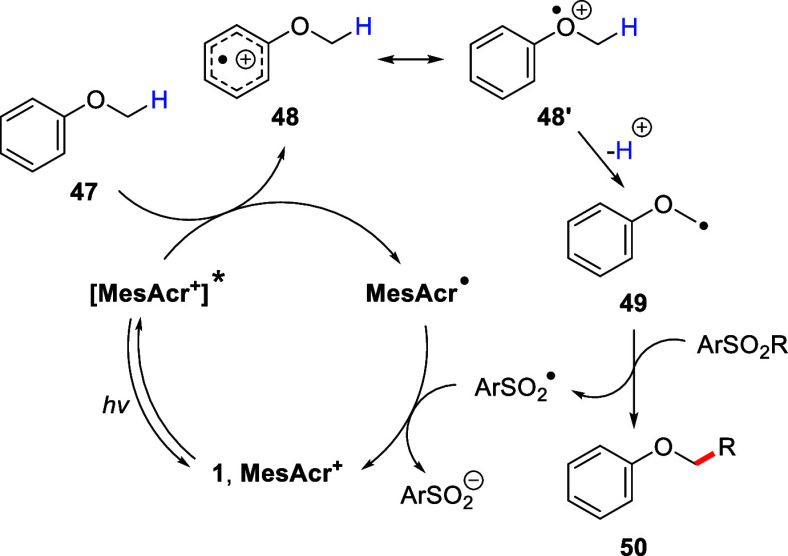
Proposed Reaction Mechanism

In conclusion, the highly selective, direct α-aryloxyalkyl C–H cyanation and allylation of aryl alkyl ethers has been achieved through the organophotoredox-mediated formation of an α-aryloxyalkyl radical which then underwent either cyanation or allylation through an addition-β-elimination process liberating a sulfonyl radical. Under the optimized conditions, an array of aryl alkyl ether substrates bearing several functional groups around the ring was selectively cyanated as well as ethers with modified alkyl chains. A similarly mixed scope of ethers was also successfully allylated exclusively at the α-aryloxyalkyl position. The applicability of the allylation procedure was then highlighted by further functionalization of products **30** and **35** using the developed reaction conditions to couple a second aryl alkyl ether utilizing the installed alkene group as a radical acceptor. A mechanism for this process has also been suggested based on previous findings.

## Data Availability

The data underlying this study are available in the published article and its Supporting Information.

## Supporting Information Available

Experimental procedures and characterization data for compounds, as well as copies of ^1^H and ^13^C NMR spectra and LCMS data (PDF)
