# Supplementary material for: Direct, Selective α-Aryloxyalkyl Radical Cyanation and Allylation of Aryl Alkyl Ethers
Source: Org Lett. 2024 Mar 7;26(11):2218–22. doi: 10.1021/acs.orglett.4c00392 (PMC10964245; doi:10.1021/acs.orglett.4c00392)
Supplement: Supplementary file 1 — ol4c00392_si_001.pdf [file ol4c00392_si_001.pdf]

Direct, Selective α-Aryloxyalkyl Radical Cyanation and Allylation of Aryl Alkyl Ethers.

Iain Robb and John A. Murphy*

Department of Pure and Applied Chemistry, University of Strathclyde, 295 Cathedral Street, Glasgow G1 1XL, Scotland.

*john.murphy@strath.ac.uk

Table of Contents

General Experimental Details SI3

Experimental Procedures and Data For All Compounds SI4

- Catalyst Synthesis SI4
- Ether Substrate Synthesis SI6
- Allylic Sulfone Substrate Synthesis SI8
- General Procedures SI11
- Optimization SI12
- Cyanation Reactions and Products SI12
- Allylation Reactions and Products SI17
- Further Functionalization of Allylation Products **30** and **35** SI25

Appendix 1: ^{1}H and ^{13}C NMR Spectra SI27

- Catalyst Synthesis S27
- Aryl Alkyl Ether Substrates SI31
- Allylic Sulfone Substrates SI35
- Cyanation Products SI41
- Allylation Products SI52
- Further Functionalization Products **44** and **46** SI67

Appendix 2: ^{1}H and ^{13}C NMR Spectra of Complex Mixtures of Diastereomers of Compound **46** SI69

Complex Mixture 1 .... SI69
....
Complex Mixture 2 .... SI70
Appendix 3: LCMS Data SI71
Complex Mixture 1 of Product **46** SI71
Complex Mixture 2 of Product **46** SI72
Appendix 4: Crude ^{1}H NMR Spectra Used For Calculation of NMR Yields SI74
Appendix 5: Example Calculation of NMR Yield SI75
References SI76

General Experimental Details

All reagents and starting materials were obtained from commercial sources and used as received. Dry 1,2-dichloroethane was obtained from Acros Organics, dry acetonitrile was obtained from Sigma-Aldrich, methanol was dried over 4 Å molecular sieves and dry DCM, along with other dry solvents, was purified using a PureSolv 400 solvent purification system. The reagent tetramethylethylenediamine was dried over CaH_2 and distilled before use. All reactions which required elevated reaction temperatures were heated in an oil bath. All photocatalytic reactions were performed in an oven-dried 2 – 5 mL or 5 – 20 mL Kinesis KX Microwave Vial under an atmosphere of argon and were sparged with argon for 10 minutes prior to irradiation. A Kessil® LED Photoreaction Light PR160L 456 nm was used as 456 nm light source in photocatalytic reactions and a Kessil® LED Photoreaction Light PR160L 390 nm was used as 390 nm light source in photocatalytic reactions. Brine refers to a saturated solution of sodium chloride. Flash column chromatography was carried out using silica gel (Fisher matrix silica 60). Merck aluminium-backed plates pre-coated with silica gel 60 (UV254) were used for thin-layer chromatography and visualised by staining with $KMnO_4$. ^1H NMR and ^{13}C NMR spectra were recorded on a Bruker DPX 400 spectrometer with chemical shift values in ppm relative to residual solvent signals as the internal standard, for ^1H NMR spectra: ^1H δ_H = 7.26 ppm; for ^{13}C NMR spectra $CDCl_3$ δ_C = 77.2 ppm. The following multiplet abbreviations are used: s = singlet, d = doublet, t = triplet, q = quartet, quin = quintet, sept = septet, m = multiplet, app. = apparent. The processed spectra can be found in the Appendices. IR spectra were recorded on an Agilent Technologies 5500 Series FTIR instrument. Melting points were determined on a Griffin platform melting point apparatus. GC-MS analysis was performed on a Thermo Finnigan Polaris Q, mass range 50-650 Da. The column temperature was 320 °C, and the carrier gas was helium with a flow rate of 1 mL/min. The adsorbent was Crossbond® (0.25 µm) with column dimensions of 30 m x 0.25 mm. Results are reported as *m/z*. All samples were prepared in $CHCl_3$, and electron ionization (EI) was utilized as the ionization method. LCMS analysis was performed on an Agilent Technologies 1200 series LC system connected to an Agilent 6130 MS mass detector. The LC was equipped with an Agilent column – Poroshell 20, 4.6 x 7.5 mm, 2.7 µm, EC-C18 with a mobile phase of H_2O + 0.1% formic acid. Results are reported as *m/z*. All samples were prepared in MeOH and electrospray ionization (ESI) was utilized as the ionization method. HRMS analysis was performed on a ThermoScientific Vanquish UHPLC system connected to a ThermoScientific Exactive Plus Orbi-Trap mass detector. The UHPLC was equipped with a Phenomenex Kinetex 1.7um C18 100 Å LC column 30 x 2.1 mm with a mobile phase of MeOH + 0.1% formic acid. Results are reported as *m/z*. All samples were prepared in MeOH and either ESI or atmospheric pressure chemical ionization (APCI) was utilized as the ionization method.

Experimental Procedures and Data For All Compounds

Catalyst Synthesis

Catalyst **1**, **MesAcr$^+$** was prepared following a literature procedure reported by the Nicewicz group.[1]

3,3'-Oxybis(*tert*-butylbenzene) (**S3**)[1]

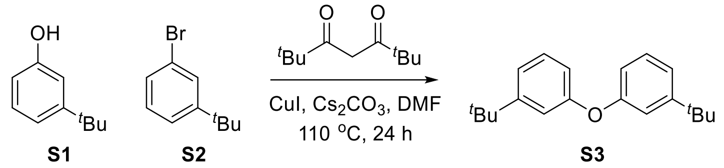

To a round-bottom flask equipped with a stirrer bar was added Cs_2CO_3 (15.3 g, 46.9 mmol), copper(I) iodide (447 mg, 2.35 mmol), 3-(*tert*-butyl)phenol (**S1**) (5.3 g, 35.2 mmol), 2,2,6,6-tetramethyl-3,5-heptanedione (490 µL, 2.35 mmol), 1-bromo-3-(*tert*-butyl)benzene (**S2**) (4 mL, 23.5 mmol) and DMF (5 mL). The reaction mixture was then heated to 110 °C for 24 h. The reaction was cooled to RT and concentrated *in vacuo*. Toluene was added in order to azeotropically remove remaining DMF. The residue was then suspended in Et_2O (100 mL), filtered through celite and the celite was washed with Et_2O until washings ran clear. The filtrate was washed with water (2 x 50 mL), sat. aq. brine (50 mL), passed through a hydrophobic frit and concentrated *in vacuo* which gave a brown liquid. Crude material was purified by column chromatography using 0 – 3% EtOAc in hexane as eluent which gave 3,3'-oxybis(*tert*-butylbenzene) (**S3**) (5.51 g, 19.5 mmol, 83%) as a colourless oil. ^{1}H NMR (400 MHz, $CDCl_3$) δ 7.25 (t, J = 7.9 Hz, 2H, 2 x ArH), 7.12 (ddd, J = 7.8, 1.8, 1.0 Hz, 2H, 2 x ArH), 7.09 (t, J = 2.1 Hz, 2H, 2 x ArH), 6.79 (ddd, J = 8.1, 2.4, 1.0 Hz, 2H, 2 x ArH), 1.30 (s, 18H, 6 x CH_3); ^{13}C NMR (101 MHz, $CDCl_3$) δ 156.5, 152.9, 128.6, 119.5, 115.7, 115.0, 34.3, 30.8; IR ν_{max}/cm^{-1} (neat): 3069 (w), 3026 (w), 2960 (m), 2866 (w), 1572 (m), 1483 (m), 1221 (s); *m/z* (ESI): 283.2 [*M*+H]$^+$. The NMR spectral data are in agreement with the literature.[1]

Methyl 2,4,6-trimethylbenzoate (**S5**)[1]

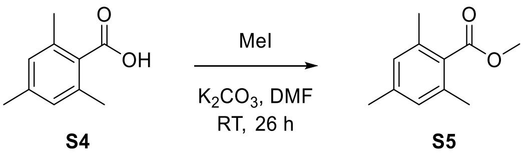

To a round-bottom flask equipped with a stirrer bar was added 2,4,6-trimethylbenzoic acid (**S4**) (3.29 g, 20.0 mmol), K_2CO_3 (4.14 g, 30.0 mmol) and DMF (25 mL). Iodomethane (1.5 mL, 24.0 mmol) was then slowly added with stirring and the reaction was left to stir at RT for 26 h. The reaction mixture was then poured into water (150 mL) and extracted with Et_2O (3 x 150 mL). The organics were combined, washed with water (3 x 75 mL), sat. aq. brine (2 x 75 mL), passed through a hydrophobic frit and concentrated *in vacuo* which gave a yellow liquid. Crude material was purified by passing through a pad of silica using 1:1 EtOAc:hexane as eluant which gave methyl 2,4,6-trimethylbenzoate (**S5**) (3.44 g, 19.3 mmol, 96%) as a pale-yellow oil.

^{1}H NMR (400 MHz, $CDCl_3$) δ 6.85 (s, 2H, 2 x ArH), 3.89 (s, 3H, CH_3), 2.28 (s, 9H, 3 x CH_3); ^{13}C NMR (101 MHz, $CDCl_3$) δ 170.1, 138.8, 134.7, 130.4, 127.9, 51.2, 20.6, 19.2; IR ν_{max}/cm^{-1} (neat): 2992 (w), 2949 (w), 2922 (w), 1724 (s), 1611 (m), 1435 (m), 1263 (vs); *m/z* (EI): 178 ([M]$^+$, 90), 163 (60), 147 (100), 91 (100), 77 (80). The NMR spectral data are in agreement with the literature.[1]

3,6-Di-*tert*-butyl-9-mesitylxanthylium tetrafluoroborate (**S6**)[1]

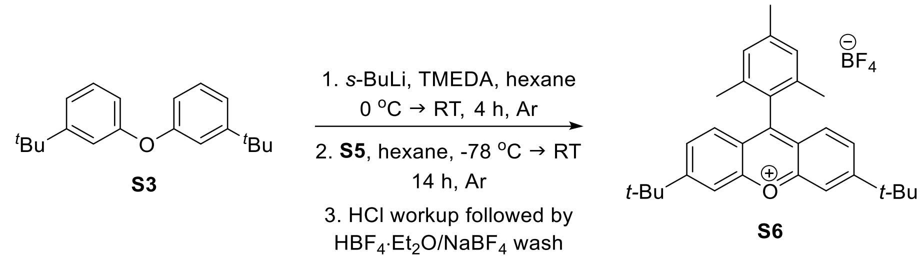

TMEDA (10 mL) was heated under reflux with CaH_2 (1.00 g) for 2 h. The TMEDA was then distilled off and placed under Ar. To an oven-dried round-bottom flask equipped with a stirrer bar under Ar was added anhydrous *n*-hexane (18 mL), 3,3'-oxybis(*tert*-butylbenzene) (**S3**) (5.00 g, 17.7 mmol) and TMEDA (4.00 mL, 26.7 mmol). The resulting solution was cooled in an ice-bath, *sec*-BuLi (1.4 M solution in cyclohexane, 33 mL, 46.2 mmol) was added dropwise, a yellow/orange colour developed and the reaction mixture was stirred at RT for 4 h. The reaction mixture was then cooled to -78 °C, a solution of methyl 2,4,6-trimethylbenzoate (**S5**) (3.20 g, 17.9 mmol) in anhydrous *n*-hexane (18 mL) was added slowly *via* syringe, a deep purple/black colour developed and reaction was left to stir at RT for 14 h. The reaction was quenched with water (15 mL), stirred for a further 30 mins and the reaction mixture turned a green colour. The reaction mixture was then diluted with Et_2O (60 mL), the layers were separated, and the organics washed with water (2 x 90 mL) and sat. aq. brine (90 mL). The organics were then stirred vigorously while conc. HCl (8 mL) was added. This solution was then diluted with water (90 mL), the layers were separated, and the organics extracted with water (3 x 90 mL). To the combined aqueous was added $NaBF_4$ (5.83 g, 53.1 mmol) which gave a yellow precipitate. This suspension was extracted with DCM (7 x 70 mL) until the extracts became colourless. To the combined organics was added $HBF_4{\cdot}Et_2O$ (2.40 mL, 17.7 mmol). Solution was swirled to achieve homogeneity, washed with water (100 mL) and 1M aq. $NaBF_4$ (100 mL), passed through a hydrophobic frit and concentrated *in vacuo* to give an orange solid. This crude was then triturated with hexane, filtered and washed with *n*-pentane which gave 3,6-di-*tert*-butyl-9-mesitylxanthylium tetrafluoroborate (**S6**) (4.00 g, 8.03 mmol, 45%) as an orange solid. Decomp. Temp. 154 °C; ^{1}H NMR (400 MHz, $CDCl_3$) δ 8.50 (d, *J* = 1.7 Hz, 2H, 2 x ArH), 7.87 (dd, *J* = 9.0, 1.7 Hz, 2H, 2 x ArH), 7.72 (d, *J* = 8.9 Hz, 2H, 2 x ArH), 7.15 (s, 2H, 2 x ArH), 2.47 (s, 3H, CH_3), 1.85 (s, 6H, 2 x CH_3), 1.53 (s, 18H, 6 x CH_3); ^{13}C NMR (101 MHz, $CDCl_3$) δ 173.5, 170.5, 158.1, 140.8, 134.9, 128.6 (2 x C), 127.9, 127.0, 121.5, 116.4, 37.0, 29.9, 20.8, 19.7; IR ν_{max}/cm^{-1} (neat): 2961 (w), 2916 (w), 1632 (w), 1599 (vs), 1435 (m), 1051 (vs); *m/z* (ESI): 411.4 [*M*]$^+$. The NMR spectral data are in agreement with the literature.[1]

3,6-Di-*tert*-butyl-9-mesityl-*N*-phenylacridinium tetrafluoroborate (**1**, **MesAcr**$^+$)[1]

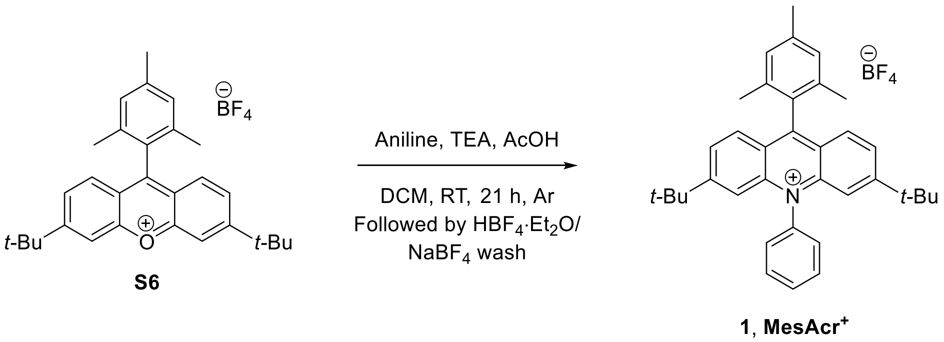

To an oven-dried round-bottom flask equipped with a stirrer bar under Ar was added 3,6-di-*tert*-butyl-9-mesitylxanthylium tetrafluoroborate (**S6**) (3.25 g, 6.52 mmol) and anhydrous DCM (10 mL). To the resulting solution was added acetic acid (1.12 mL, 19.56 mmol) and TEA (750 µL, 7.82 mmol). Aniline (1.36 mL, 9.78 mmol) was then added dropwise. The flask was covered with aluminium foil and left to stir at RT for 21 h. The reaction mixture was transferred to a separatory funnel and washed with water (30 mL) and sat. aq. $NaHCO_3$ (30 mL). To the organics was added $HBF_4{\cdot}Et_2O$ (900 µL, 6.52 mmol). The solution was swirled to achieve homogeneity, washed with water (50 mL) and 1M aq. $NaBF_4$ (50 mL). The organic layer was then passed through a hydrophobic frit and concentrated *in vacuo*. The red/orange residue was triturated with 2:1 hexane:Et_2O, filtered and the solid washed with *n*-pentane which gave 3,6-di-*tert*-butyl-9-mesityl-*N*-phenylacridinium tetrafluoroborate (**1**, **MesAcr**$^+$) (3.15 g, 5.49 mmol, 84%) as a golden yellow solid. Decomp. Temp. 162 °C; ^{1}H NMR (400 MHz, $CDCl_3$) δ 7.99 – 7.92 (m, 2H, 2 x ArH), 7.92 – 7.86 (m, 1H, ArH), 7.82 – 7.71 (m, 6H, 6 x ArH), 7.42 (s, 2H, 2 x ArH), 7.16 (s, 2H, 2 x ArH), 2.49 (s, 3H, CH_3), 1.86 (s, 6H, 2 x CH_3), 1.29 (s, 18H, 6 x CH_3); ^{13}C NMR (101 MHz, $CDCl_3$) δ 163.0, 161.8, 141.7, 139.7, 136.4, 135.7, 131.3, 131.1, 128.8, 128.4, 127.8, 127.6, 126.9, 123.6, 114.6, 36.2, 29.7, 20.8, 19.7; IR ν_{max}/cm^{-1} (neat): 2959 (w), 2911 (w), 1611 (m), 1430 (m), 1049 (vs); *m/z* (ESI): 486.4 [*M*]$^+$. The NMR spectral data are in agreement with the literature.[1]

Ether Substrate Synthesis

(Hexyloxy)benzene (**S9**)

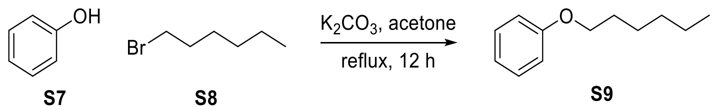

To a round-bottom flask equipped with a stirrer bar was added phenol (**S7**) (847 mg, 9.00 mmol), K_2CO_3 (1.10 g, 8.00 mmol), 1-bromohexane (**S8**) (845 µL, 6.00 mmol) and acetone (20 mL). The resulting heterogeneous reaction mixture was heated under reflux for 5 h. More phenol (188 mg, 2.00 mmol) and K_2CO_3 (412 mg, 3.00 mmol) was then added and the reaction mixture was heated under reflux for a further 5 h. More K_2CO_3 (276 mg, 2.00 mmol) was then added and the reaction mixture was heated under reflux for a further 2 h. The reaction mixture was then concentrated *in vacuo* and residue was dissolved in Et_2O (50 mL) and washed with

2N NaOH (2 x 50 mL), sat. aq. brine (50 mL), passed through a hydrophobic frit and concentrated *in vacuo* which gave (hexyloxy)benzene (**S9**) (911 mg, 5.11 mmol, 85%) as a colourless oil. ^{1}H NMR (400 MHz, $CDCl_3$) δ 7.30 – 7.24 (m, 2H, 2 x ArH), 6.96 – 6.86 (m, 3H, 3 x ArH), 3.95 (t, *J* = 6.6 Hz, 2H, CH_2), 1.83 – 1.73 (m, 2H, CH_2), 1.52 – 1.41 (m, 2H, CH_2), 1.39 – 1.29 (m, 4H, 2 x CH_2), 0.96 – 0.86 (m, 3H, CH_3); ^{13}C NMR (101 MHz, $CDCl_3$) δ 158.6, 128.9, 119.9, 114.0, 67.4, 31.1, 28.8, 25.2, 22.1, 13.5; IR ν_{max}/cm^{-1} (neat): 2953 (m), 2930 (m), 2858 (m), 1599 (m), 1585 (m), 1497 (s); *m/z* (ESI): 178.1 [*M*]$^+$. The NMR spectral data are in agreement with the literature.[2]

3-Phenoxypropylbenzene (**S11**)[3]

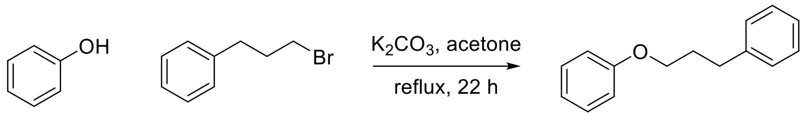

S7 **S10** **S11**

To a round-bottom flask equipped with a stirrer bar was added phenol (**S7**) (1.89 g, 20.1 mmol), K_2CO_3 (2.78 g, 5.02 mmol), 1-bromo-3-phenylpropane (**S10**) (765 µL, 5.02 mmol) and acetone (50 mL). The resulting heterogeneous reaction mixture was heated under reflux for 22 h. The reaction mixture was then concentrated *in vacuo* and residue was dissolved in Et_2O (50 mL) and washed with 2N NaOH (2 x 50 mL), sat. aq. brine (50 mL), passed through a hydrophobic frit and concentrated *in vacuo* which gave a yellow liquid. Crude material was purified by column chromatography using 10% Et_2O in hexane as eluent which gave 3-phenoxypropylbenzene (**S11**) (998 mg, 4.70 mmol, 94%) as a colourless liquid. ^{1}H NMR (400 MHz, $CDCl_3$) δ 7.33 – 7.26 (m, 4H, 4 x ArH), 7.25 – 7.17 (m, 3H, 3 x ArH), 6.98 – 6.87 (m, 3H, 3 x ArH), 3.97 (t, *J* = 6.3 Hz, 2H, CH_2), 2.82 (t, *J* = 7.7 Hz, 2H, CH_2), 2.17 – 2.06 (m, 2H, CH_2); ^{13}C NMR (101 MHz, $CDCl_3$) δ 158.5, 141.1, 128.9, 128.0, 127.9, 125.4, 120.1, 114.0, 66.3, 31.7, 30.4; IR ν_{max}/cm^{-1} (neat): 3015 (w), 2944 (m), 2933 (m), 2849 (m), 1592 (m), 1588 (m), 1491 (s); *m/z* (ESI): 212.2 [*M*]$^+$. The NMR spectral data are in agreement with the literature.[3]

(Cyclopentyloxy)benzene (**S13**)[3]

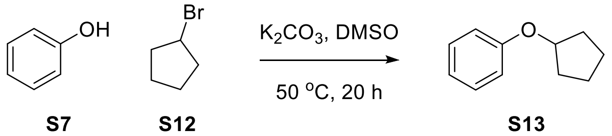

S7 **S12** **S13**

To a round-bottom flask equipped with a stirrer bar was added phenol (**S7**) (235 mg, 2.50 mmol), K_2CO_3 (691 mg, 5.00 mmol) and DMSO (5 mL). Bromocyclopentane (**S12**) (535 µL, 5.00 mmol) was then added and the resulting reaction mixture was stirred at 50 °C for 20 h. The reaction mixture was then poured onto water (20 mL) and extracted with Et_2O (3 x 15 mL). The organics were then combined, washed with sat. aq. brine (20 mL), passed through a hydrophobic frit and concentrated *in vacuo* which gave a colourless oil. This crude material was then purified by column chromatography using 5% Et_2O in hexane as eluent which gave

(cyclopentyloxy)benzene (**S13**) (257 mg, 1.58 mmol, 63%) as a colourless oil. ^{1}H NMR (400 MHz, $CDCl_3$) δ 7.29 – 7.23 (m, 2H 2 x ArH), 6.94 – 6.84 (m, 3H, 3 x ArH), 4.76 (m, 1H, CH), 1.96 – 1.74 (m, 6H, 3 x CH_2 protons), 1.67 – 1.56 (m, 2H, CH_2 protons); ^{13}C NMR (101 MHz, $CDCl_3$) δ 157.6, 128.8, 119.7, 115.1, 78.6, 32.3, 23.5; IR ν_{max}/cm^{-1} (neat): 3023 (w), 2995 (w), 2951 (w), 2833 (w), 1601 (m), 1591 (m), 1495 (s); *m/z* (ESI): 162.1 [*M*]$^{+}$. The NMR spectral data are in agreement with the literature.[3]

2-(3-Phenoxypropyl)isoindoline-1,3-dione (**S15**)[4]

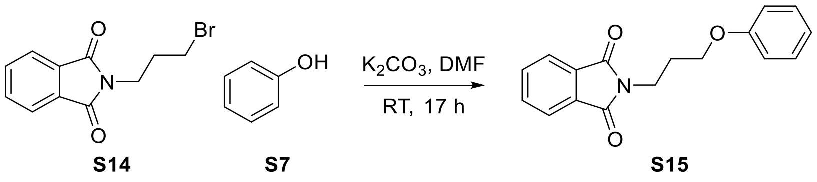

To a round-bottom flask equipped with a stirrer bar was added phenol (**S7**) (565 mg, 6.00 mmol), K_2CO_3 (1.40 g, 10.0 mmol), *N*-(3-bromopropyl)pthalimide (**S14**) (1.34 g, 5.00 mmol) and DMF (10 mL). The resulting heterogeneous reaction mixture was stirred at RT for 17 h. The reaction mixture was then quenched with water (10 mL) and extracted with EtOAc (3 x 10 mL). The organics were combined, washed with sat. aq. brine (3 x 30 mL), passed through a hydrophobic frit and concentrated *in vacuo* which gave a white solid. NMR analysis showed remaining phenol starting material and DMF. As a result, the white solid was dissolved in Et_2O (30 mL) washed with 2N NaOH (2 x 50 mL), sat. aq. brine (3 x 30 mL), passed through a hydrophobic frit and concentrated *in vacuo* which gave 2-(3-phenoxypropyl)isoindoline-1,3-dione (**S11**) (1.19 g, 4.23 mmol, 85%) as a white solid. M.pt: 86 – 88 °C (lit: 88 – 90 °C)[5]; ^{1}H NMR (400 MHz, $CDCl_3$) δ 7.90 – 7.84 (m, 2H, 2 x ArH), 7.76 – 7.71 (m, 2H, 2 x ArH), 7.28 – 7.23 (m, 2H, 2 x ArH), 6.98 – 6.91 (m, 1H, ArH), 6.87 – 6.81 (m, 2H, 2 x ArH), 4.06 (t, *J* = 6.1 Hz, 2H, CH_2), 3.94 (t, *J* = 6.9 Hz, 2H, CH_2), 2.21 (app. quin, *J* = 6.8 Hz, 2H, CH_2); ^{13}C NMR (101 MHz, $CDCl_3$) δ 167.9, 158.2, 133.4, 131.7, 128.9, 122.7, 120.3, 114.0, 65.0, 35.0, 27.8; IR ν_{max}/cm^{-1} (neat): 3053 (w), 2951 (w), 2934 (w), 1769 (m), 1697 (vs), 1601 (m), 1585 (m); *m/z* (ESI): 282.1 [*M*+H]$^{+}$. The NMR spectral data are in agreement with literature.[4]

Allylic Sulfone Substrate Synthesis

(Prop-2-ene-1,2-diyldisulfonyl)dibenzene (**S17**)[6]

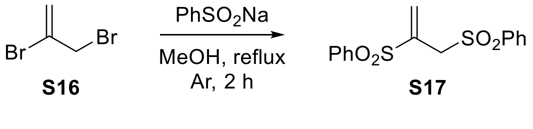

To an oven-dried round-bottom flask equipped with a stirrer bar and condenser under Ar was added sodium benzenesulfinate (3.28 g, 20.0 mmol) along with anhydrous MeOH (50 mL). 2,3-Dibromopropene (**S16**) (978 µL, 10.0 mmol) was then added and the resulting reaction mixture was refluxed for 2 h. The reaction mixture was allowed to cool to RT before being filtered and washed with more MeOH. The filtrate was then concentrated *in vacuo* which gave a pale-yellow solid. DCM was then added to this solid which gave a suspension which was again filtered. The resulting filtrate was then concentrated *in vacuo* which gave an

orange solid. Crude material was purified by column chromatography using 0 – 50% EtOAc in hexane as eluent which gave (prop-2-ene-1,2-diyldisulfonyl)dibenzene (**S17**) (1.31 g, 4.06 mmol, 41%) as a white solid. M.pt: 126 – 128 °C (lit: 128 – 129 °C)[7]; ^{1}H NMR (400 MHz, $CDCl_3$) δ 7.81 – 7.71 (m, 4H, 4 x ArH), 7.70 – 7.59 (m, 2H, 2 x ArH), 7.57 – 7.47 (m, 4H, 4 x ArH), 6.68 (d, *J* = 1.3 Hz, 1H, CH_2), 6.52 (app. q, *J* = 1.1 Hz, 1H, CH_2), 4.06 (d, *J* = 1.0 Hz, 2H, CH_2); ^{13}C NMR (101 MHz, $CDCl_3$) δ 139.0, 137.3 (2 x C), 133.8, 133.6, 130.7, 128.9, 128.8, 128.0, 127.9, 53.7; IR ν_{max}/cm^{-1} (neat): 3073(w), 2974 (w), 2907 (w), 1585 (w), 1448 (m), 1301 (s), 1139 (s), 1072 (s); *m/z* (ESI): 340.1 [*M*+H_2O]$^+$. The NMR spectral data are in agreement with literature.[6]

Ethyl 2-((phenylsulfonyl)methyl)acrylate (**S19**)[8]

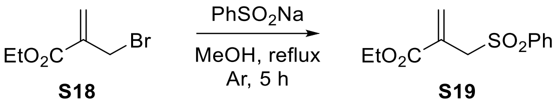

S18 **S19**

To an oven-dried round-bottom flask equipped with a stirrer bar and condenser under Ar was added sodium benzenesulfinate (616 mg, 3.75 mmol) followed by ethyl 2-(bromomethyl)acrylate (**S18**) (345 µl, 2.50 mmol) in solution with anhydrous MeOH (15 mL). The resulting reaction mixture was heated to reflux for 5 h before being concentrated *in vacuo*. The residue was then partitioned between water (20 mL) and DCM (20 mL), the layers were separated and the aqueous layer was extracted with DCM (3 x 20 mL). The organics were then combined, passed through a hydrophobic frit and concentrated *in vacuo* which gave a faint orange gum. Crude material was purified by column chromatography using 0 – 50% EtOAc in hexane as eluent which gave ethyl 2-((phenylsulfonyl)methyl)acrylate (**S19**) (508 mg, 2.00 mmol, 80%) as a colourless oil. ^{1}H NMR (400 MHz, $CDCl_3$) δ 7.90 – 7.83 (m, 2H, 2 x ArH), 7.68 – 7.61 (m, 1H, ArH), 7.58 – 7.50 (m, 2H, 2 x ArH), 6.51 (s, 1H, CH_2), 5.92 (s, 1H, CH_2), 4.16 (s, 2H, CH_2), 4.01 (q, *J* = 7.1 Hz, 2H, CH_2), 1.17 (t, *J* = 7.1 Hz, 3H, CH_3); ^{13}C NMR (101 MHz, $CDCl_3$) δ ^{13}C NMR (101 MHz, $CDCl_3$) δ 164.2, 137.9, 133.3, 132.8, 128.6, 128.5, 128.3, 61.0, 57.0, 13.5; IR ν_{max}/cm^{-1} (neat): 3065 (w), 2989 (w), 2941 (w), 1716 (s), 1451 (m), 1310 (s), 1147 (vs), 1087 (s); *m/z* (ESI): 255.2 [*M*+H]$^+$. The NMR spectral data are in agreement with literature.[9]

1-Phenyl-2-((phenylsulfonyl)methyl)prop-2-en-1-one (**S21**)[8]

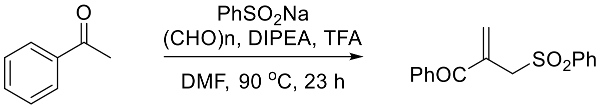

S20 **S21**

To a round-bottom flask equipped with a stirrer bar was added sodium benzenesulfinate (657 mg, 4.00 mmol), paraformaldehyde (480 mg, 16.0 mmol), acetophenone (**S20**) (467 µL, 4.00 mmol), DIPEA (697 µL, 4.00 mmol), TFA (613 µL, 8.00 mmol) and DMF (40 mL). The flask was then sealed and the resulting reaction mixture was stirred at 90 °C for 23 h. The reaction mixture was then diluted with EtOAc (40 mL), washed with sat. aq. LiCl (3 x 30 mL), passed through a hydrophobic frit and concentrated *in vacuo* which gave an

orange oil. The combined aqueous washings were then re-extracted with EtOAc (3 x 30 mL). These organics were then combined, washed with sat. aq. LiCl (2 x 50 mL), passed through a hydrophobic frit and concentrated *in vacuo* which gave an orange oil. The combined crude material was then purified by column chromatography using 0 – 40% EtOAc in hexane as eluent which gave 1-phenyl-2-((phenylsulfonyl)methyl)prop-2-en-1-one (**S21**) (400 mg, 1.40 mmol, 35%) as a white solid. M.pt: 98 – 100 °C (lit: 99 – 100 °C)[10]; ^{1}H NMR (400 MHz, $CDCl_3$) δ 7.93 – 7.90 (m, 2H, 2 x ArH), 7.68 – 7.64 (m, 2H, 2 x ArH), 7.63 – 7.49 (m, 4H, 4 x ArH), 7.46 – 7.40 (m, 2H, 2 x ArH), 6.29 (s, 1H, CH_2), 6.04 (s, 1H, CH_2), 4.37 (d, J = 0.8 Hz, 2H, CH_2); ^{13}C NMR (101 MHz, $CDCl_3$) δ 194.2, 138.4, 135.7, 135.2, 133.6, 133.4, 132.1, 129.1, 128.7, 127.9, 127.8, 57.2; IR ν_{max}/cm^{-1} (neat): 3104 (w), 3067 (w), 2989 (w), 2935 (w), 1652 (m), 1449 (m), 1303 (s), 1152 (s); *m/z* (ESI): 287.1 $[M+H]^+$. The NMR spectral data are in agreement with literature.[10]

((2-Bromoallyl)sulfonyl)benzene (**S22**)[8]

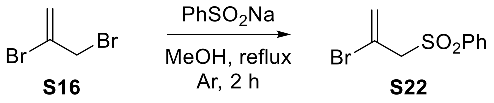

To an oven-dried round-bottom flask equipped with a stirrer bar and condenser under Ar was added sodium benzenesulfinate (591 mg, 3.60 mmol) along with anhydrous MeOH (15 mL). 2,3-Dibromopropene (**S16**) (294 µL, 3.00 mmol) was then added and the resulting reaction mixture was refluxed for 2 h. The reaction mixture was allowed to cool to RT before being concentrated *in vacuo* which gave an off-white solid. DCM was then added to this solid which gave a suspension which was again filtered. The resulting filtrate was then concentrated *in vacuo* which gave an orange solid. Crude material was purified by column chromatography using 0 – 30% EtOAc in hexane as eluent which gave ((2-bromoallyl)sulfonyl)benzene (**S22**) (283 mg, 1.08 mmol, 36%) as a white solid. M.pt: 77 – 79 °C (lit: 78 – 79 °C)[11]; ^{1}H NMR (400 MHz, $CDCl_3$) δ 7.97 – 7.92 (m, 2H, 2 x ArH), 7.72 – 7.66 (m, 1H. ArH), 7.61 – 7.56 (m, 2H, 2 x ArH), 5.86 – 5.83 (m, 1H, CH_2), 5.78 (d, J = 2.3 Hz, 1H, CH_2), 4.15 (d, J = 0.8 Hz, 2H, CH_2); ^{13}C NMR (101 MHz, $CDCl_3$) δ 137.5, 133.7, 128.7, 128.4, 126.1, 116.7, 66.0; IR ν_{max}/cm^{-1} (neat): 3071 (w), 2985 (w), 2922 (w), 1586 (w), 1625 (w), 1448 (m), 1305 (s), 1141 (s) 689 (vs); *m/z* (ESI): 278.0 $[M+H_2O]^+$. The NMR spectral data are in agreement with literature.[11]

(3-Bromoprop-1-en-2-yl)benzene (**S24**)[12]

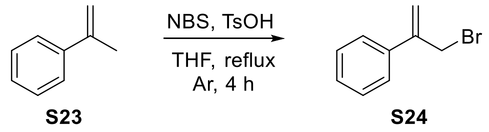

To an oven-dried 20 ml microwave vial containing a stirrer bar was added *N*-bromosuccinimide (935 mg, 5.25 mmol), *p*-toluenesulfonic acid monohydrate (95 mg, 0.50 mmol) and α-methylstyrene (**S23**) (650 µL, 6.00 mmol). The vial was then sealed and purged with Ar. Anhydrous THF (12 mL) was added and the reaction

mixture was sparged with Ar for 10 mins. The resulting reaction mixture was then stirred under reflux for 4 h before being diluted with EtOAc (30 mL) and washed with water (3 x 40 mL). The organic layer was then passed through a hydrophobic frit and concentrated *in vacuo* which gave (3-bromoprop-1-en-2-yl)benzene (**S24**) (920 mg, 4.67 mmol, 93%) as an orange liquid. This crude material was then used in a further reaction without further purification.

((2-Phenylallyl)sulfonyl)benzene (**S25**)[8]

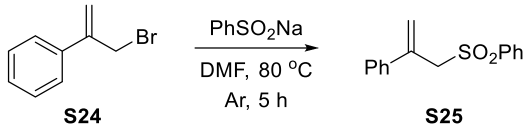

To an oven-dried 20 ml microwave vial containing a stirrer bar was added sodium benzenesulfinate (591 mg, 3.60 mmol) before the vial was sealed and purged with Ar. (3-Bromoprop-1-en-2-yl)benzene (**S24**) (920 mg, 4.67 mmol) in solution with anhydrous DMF (14 mL) was then added and the resulting reaction mixture was stirred at 80 °C for 5 h. The reaction mixture was then diluted with EtOAc (30 mL), washed with sat. aq. LiCl (3 x 30 mL), passed through a hydrophobic frit and concentrated *in vacuo* which gave an orange oil. Crude material was purified by column chromatography using 0 – 30% EtOAc in hexane as eluent which gave ((2-phenylallyl)sulfonyl)benzene (**S25**) (567 mg, 2.20 mmol, 47%) as a white solid. M.pt: 69 – 71 °C (lit: 73 – 75 °C)[13]; ^{1}H NMR (400 MHz, $CDCl_3$) δ 7.82 – 7.75 (m, 2H, 2 x ArH), 7.59 – 7.51 (m, 1H, ArH), 7.47 – 7.39 (m, 2H, 2 x ArH), 7.30 – 7.20 (m, 5H, 5 x ArH), 5.60 (s, 1H, CH_2), 5.23 (s, 1H, CH_2), 4.28 (s, 2H, CH_2); ^{13}C NMR (101 MHz, $CDCl_3$) δ 138.3, 138.0, 136.0, 133.1, 128.4, 128.2, 127.9, 127.6, 125.7, 121.3, 61.6; IR ν_{max}/cm^{-1} (neat): 3059 (w), 2986 (w), 2938 (w), 1621 (w), 1586 (s), 1448 (m), 1301 (s), 1139 (s); *m/z* (ESI): 259.1 [*M*+H]$^+$. The NMR spectral data are in agreement with literature.[14]

General Procedures

General procedure A - To an oven-dried 5 ml microwave vial containing a stirrer bar was added 3,6-di-*tert*-butyl-9-mesityl-*N*-phenylacridinium tetrafluoroborate (18 mg, 0.030 mmol), Na_2HPO_4 (128 mg, 0.900 mmol), aryl sulfone (0.900 mmol, 3.0 eq.) and ether substrate (0.300 mmol, 1.0 eq.). The vial was then sealed and purged with Ar. Anhydrous DCM (such that ether substrate concentration would be 0.1 M) was added and the reaction mixture was sparged with Ar for 10 min. The reaction was then irradiated with blue LEDs (456 nm) from a Kessil lamp (3 cm away from the vial) for 24 h. The reaction mixture was passed through a silica plug using DCM and concentrated *in vacuo* and crude material was purified by column chromatography.

In two 'general procedure A' experiments which are noted in the SI, larger scale reactions were performed, for which the quantities were modified as specified in those experiments, but the irradiation time and the irradiation source are the same.

General procedure B - To an oven-dried 5 ml microwave vial containing a stirrer bar was added 3,6-di-*tert*-butyl-9-mesityl-*N*-phenylacridinium tetrafluoroborate (18 mg, 0.030 mmol), Na_2HPO_4 (128 mg, 0.900 mmol),

aryl sulfone (0.900 mmol, 3.0 eq.) and ether substrate (0.300 mmol, 1.0 eq.). The vial was then sealed and purged with Ar. Anhydrous DCM (3 mL, such that ether substrate concentration would be 0.1 M) was added and the reaction mixture was sparged with Ar for 10 min. The reaction was then irradiated with blue LEDs (456 nm) from a Kessil lamp (3 cm away from the vial) for 24 h. The reaction mixture was passed through a silica plug using DCM and concentrated *in vacuo*. Tetrachloroethane (28 µL) was then added to the residue along with $CDCl_3$ (6 mL). Approximately 0.6 mL of this solution was then submitted for NMR analysis. For an example of the calculation of the NMR yield for compound **16** using the crude ^1H spectrum shown in Appendix 4 (page SI.74) see the calculation shown in Appendix 5 (page SI. 75).

Optimization

Optimisation with 4-chloroanisole (**S26**)

All optimisation reactions were carried out according to **General procedure B** with appropriate alterations detailed in *Table S1* below. NMR yields were calculated as shown in Appendix 5.

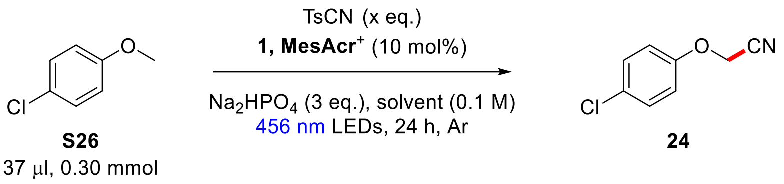

*Table S1: Optimisation with 4-chloroanisole (**S26**)*

Entry	Amount of TsCN (eq.)	Solvent	NMR yield
**1**	163 mg, 0.90 mmol (3.0)	DCE	91%
**2**	163 mg, 0.90 mmol (3.0)	MeCN	74%
**3**	**163 mg, 0.90 mmol (3.0)**	**DCM**	**95% (88%)**
**4**	96 mg, 0.45 mmol (1.5)	DCM	83%
**5**	64 mg, 0.30 mmol (1.0)	DCM	63%
**6**	163 mg, 0.90 mmol (3.0)	DCM	31%*

*Isolated yield shown in parenthesis. *10 mol% 9-Mesityl-10-methylacridinium tetrafluoroborate (Fukuzumi's Cat.) used.*

Cyanation Reactions and Products

2-Phenoxyacetonitrile (**15**)

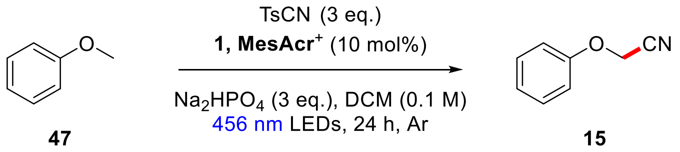

Synthesised according to **General procedure A** using tosyl cyanide (163 mg, 0.900 mmol) and anisole (**47**) (33 µL, 0.300 mmol). Purified by column chromatography using 0 – 30% Et_2O in pentane as eluent which gave 2-phenoxyacetonitrile (**15**) (29 mg, 0.218 mmol, 73%) as an orange oil. ^1H NMR (400 MHz, $CDCl_3$) δ 7.40 – 7.32 (m, 2H, 2 x ArH), 7.13 – 7.07 (m, 1H, ArH), 7.02 – 6.96 (m, 2H, 2 x ArH), 4.77 (s, 2H, CH_2);

^{13}C NMR (101 MHz, $CDCl_3$) δ 156.1, 129.4, 122.7, 114.6, 114.5, 53.1; IR ν_{max}/cm^{-1} (neat): 3053 (w), 2930 (w), 2913 (w), 2164 (vw), 1590 (w), 1493 (m); *m/z* (EI): 133.1 ($[M]^+$,100), 105.1 (6), 93.0 (95), 83.0 (36). The NMR spectral data are in agreement with literature.[15]

2-Phenoxypropanenitrile (**16**)

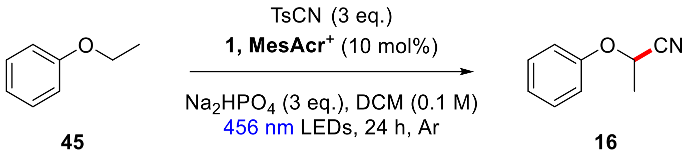

Synthesised according to **General procedure B** using tosyl cyanide (163 mg, 0.900 mmol) and phenetole (**45**) (38 µL, 0.300 mmol). Purification was attempted. However, product material co-eluted with the inseparable by-product **A** (shown below). NMR yield of 85% was calculated as shown in Appendix 5. The ^{1}H NMR of the crude reaction product is shown in Appendix 4.

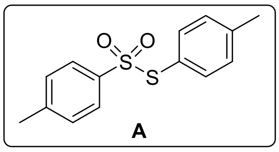

2-Methyl-2-phenoxypropanenitrile (**17**)

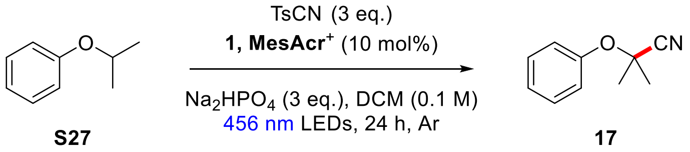

Synthesised according to **General procedure A** using tosyl cyanide (163 mg, 0.900 mmol) and isoproproxybenzene (**S27**) (44 µL, 0.300 mmol). Purified by column chromatography using 0 – 15% Et_2O in pentane as eluent which gave 2-methyl-2-phenoxypropanenitrile (**17**) (12 mg, 0.074 mmol, 25%) as a colourless oil. ^{1}H NMR (400 MHz, $CDCl_3$) δ 7.38 – 7.30 (m, 2H, 2 x ArH), 7.21 – 7.14 (m, 3H, 3 x ArH), 1.72 (s, 6H, 2 x CH_3); ^{13}C NMR (101 MHz, $CDCl_3$) δ 153.8, 129.1, 124.2, 121.3, 120.4, 71.5, 27.0; IR ν_{max}/cm^{-1} (neat): 2994 (w), 2917 (w), 2252 (vw), 1485 (m), 1223 (m); HRMS (APCI) *m/z*: $[M+H]^+$ calcd for $C_{10}H_{12}NO^+$: 162.0913; found: 162.0914.

2-Phenoxyheptanenitrile (**18**)

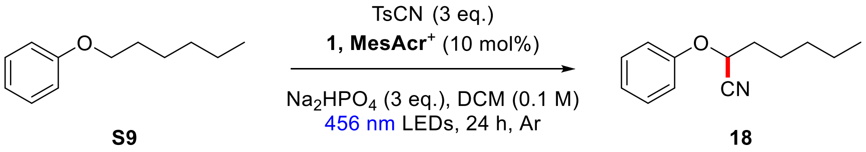

Synthesised according to **General procedure A** using tosyl cyanide (163 mg, 0.900 mmol) and (hexyloxy)benzene (**S9**) (55 mg, 0.300 mmol). Purified by column chromatography using 0 – 10% EtOAc in

hexane as eluent which gave 2-phenoxyheptanenitrile (**18**) (48 mg, 0.236 mmol, 79%) as a yellow oil. ^{1}H NMR (400 MHz, $CDCl_3$) δ 7.38 – 7.31 (m, 2H, 2 x ArH), 7.11 – 7.05 (m, 1H, ArH), 7.04 – 6.98 (m, 2H, 2 x ArH), 4.76 (t, *J* = 6.7 Hz, 1H, CH), 2.14 – 1.99 (m, 2H, CH_2), 1.68 – 1.57 (m, 2H, CH_2), 1.44 – 1.33 (m, 4H, 2 x CH_2), 0.96 – 0.89 (m, 3H, CH_3); ^{13}C NMR (101 MHz, $CDCl_3$) δ 156.1, 129.3, 122.6, 117.3, 115.4, 66.3, 33.0, 30.6, 23.9, 21.9, 13.4; IR ν_{max}/cm^{-1} (neat): 3042 (w), 2954 (w), 2930 (w), 2859 (w), 2159 (vw), 1592 (m), 1495 (m) 1221 (m); HRMS (ESI) *m/z*: $[M+H]^+$ calcd for $C_{13}H_{18}NO^+$: 204.1383; found: 204.1383.

2-Phenoxy-4-phenylbutanenitrile (**19**)

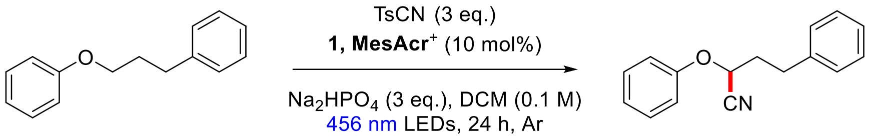

S11 **19**

Synthesised according to **General procedure A** using tosyl cyanide (163 mg, 0.900 mmol) and and 3-phenoxypropylbenzene (**S11**) (64 mg, 0.300 mmol). Purified by column chromatography using 0 – 25% Et_2O in hexane as eluent which gave 2-phenoxy-4-phenylbutanenitrile (**19**) (41 mg, 0.173 mmol, 58%) as a pale-yellow oil. ^{1}H NMR (400 MHz, $CDCl_3$) δ 7.41 – 7.31 (m, 4H, 4 x ArH), 7.28 – 7.19 (m, 3H, 3 x ArH), 7.15 – 7.08 (m, 1H, ArH), 7.04 – 6.98 (m, 2H, 2 x ArH), 4.73 (dd, *J* = 7.4, 5.9 Hz, 1H, CH), 2.98 (t, *J* = 7.3 Hz, 2H, CH_2), 2.52 – 2.32 (m, 2H, CH_2); ^{13}C NMR (101 MHz, $CDCl_3$) δ 156.0, 138.8, 129.4, 128.3, 128.0, 126.2, 122.7, 117.1, 115.3, 65.2, 34.6, 30.2; IR ν_{max}/cm^{-1} (neat): 3064 (w), 3029 (w), 2930 (w), 2863 (w), 2165 (vw), 1592 (m), 1495 (m), 1221 (m); HRMS (ESI) *m/z*: $[M+H]^+$ calcd for $C_{16}H_{16}NO^+$: 238.1226; found: 238.1226.

2-(*m*-Tolyloxy)acetonitrile (**20**)[15]

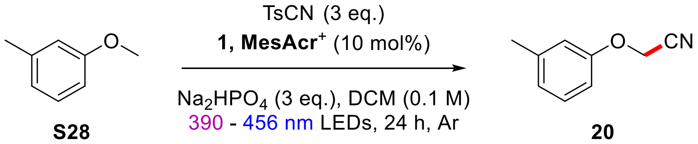

S28 **20**

Synthesised according to **General procedure A** (NMR yield (44%) was calculated as described in **General procedure B**) using tosyl cyanide (163 mg, 0.900 mmol) and 3-methylanisole (**S28**) (38 µL, 0.300 mmol). Purified by column chromatography using 0 – 20% Et_2O in pentane as eluent which gave 2-(*m*-tolyloxy)acetonitrile (**20**) (9 mg, 0.061 mmol, 20%) as a yellow oil. ^{1}H NMR (400 MHz, $CDCl_3$) δ 7.23 (t, *J* = 7.9 Hz, 1H, ArH), 6.94 – 6.87 (m, 1H, ArH), 6.83 – 6.76 (m, 2H, 2 x ArH), 4.75 (s, 2H, CH_2), 2.36 (s, 3H, CH_3); ^{13}C NMR (101 MHz, $CDCl_3$) δ 156.1, 139.7, 129.1, 123.5, 115.4, 114.7, 111.3, 53.1, 21.0; IR ν_{max}/cm^{-1} (neat): 2919 (w), 2850 (w), 2163 (vw), 1688 (w), 1703 (w), 1590 (m), 1492 (m), 1156 (s); *m/z* (EI): 147.1 ($[M]^+$,85), 107.1 (89), 77.1 (100). The NMR spectral data are in agreement with literature.[15]

<u>390 nm LEDs</u>

Synthesised according to **General procedure B** using purple LEDs (390 nm), tosyl cyanide (163 mg, 0.900 mmol) and 3-methylanisole (**S28**) (38 µL, 0.300 mmol). NMR yield of 50% was calculated as shown in Appendix 5.

2-(3-Methoxyphenoxy)acetonitrile (**21**)

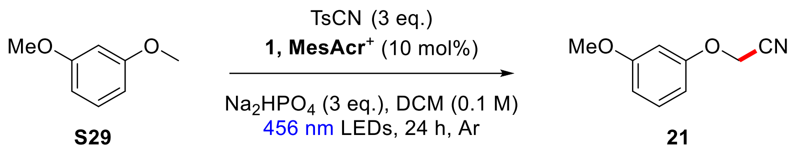

Synthesised according to **General procedure A** using tosyl cyanide (163 mg, 0.900 mmol) and 1,3-dimethoxybenzene (**S29**) (40 µL, 0.300 mmol). Purified by column chromatography using 0 – 50% Et_2O in hexane as eluent which gave 2-(3-methoxyphenoxy)acetonitrile (**21**) (37 mg, 0.227 mmol, 76%) as a pale yellow oil. ^{1}H NMR (400 MHz, $CDCl_3$) δ 7.25 (t, J = 8.2 Hz, 1H, ArH), 6.64 (ddd, J = 8.3, 2.3, 0.6 Hz, 1H, ArH), 6.57 (ddd, J = 8.3, 2.4, 0.8 Hz, 1H, ArH), 6.54 (t, J = 2.4 Hz, 1H, ArH), 4.75 (s, 2H, CH_2), 3.81 (s, 3H, CH_3); ^{13}C NMR (101 MHz, $CDCl_3$) δ 160.6, 157.3, 129.9, 114.6, 108.2, 106.1, 101.3, 54.9, 53.1; IR ν_{max}/cm^{-1} (neat): 3007 (w), 2966 (w), 2943 (w), 2839 (w), 2163 (vw), 1711 (w), 1593 (s), 1493 (s), 1150 (vs); HRMS (ESI) *m/z*: $[M+H]^+$ calcd for $C_9H_{10}NO_2^+$: 164.0706; found: 164.0705.

2-(4-Methoxyphenoxy)acetonitrile (**22**)[15]

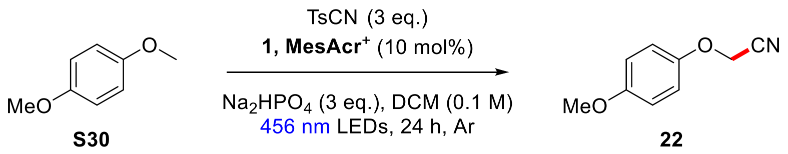

Synthesised according to **General procedure A** using tosyl cyanide (163 mg, 0.900 mmol) and 1,4-dimethoxybenzene (**S30**) (40 µL, 0.300 mmol). Purified by column chromatography using 0 – 50% Et_2O in hexane as eluent which gave 2-(4-methoxyphenoxy)acetonitrile (**22**) (11 mg, 0.067 mmol, 22%) as a pale yellow oil. ^{1}H NMR (400 MHz, $CDCl_3$) δ 7.01 – 6.94 (m, 2H, 2 x ArH), 6.93 – 6.87 (m, 2H, 2 x ArH), 4.73 (s, 2H, CH_2), 3.81 (s, 3H, CH_3); ^{13}C NMR (101 MHz, $CDCl_3$) δ 155.2, 150.2, 116.3, 114.8, 114.5, 55.2, 54.5; IR ν_{max}/cm^{-1} (neat): 3005 (w), 2936 (w), 2839 (w), 2154 (vw), 1597 (w), 1508 (vs), 1204 (s); *m/z* (EI): 163.1 ($[M]^+$, 33), 123.1 (100), 95.1 (33). The NMR spectral data are in agreement with literature.[15]

2-(4-Fluorophenoxy)acetonitrile (**23**)[15]

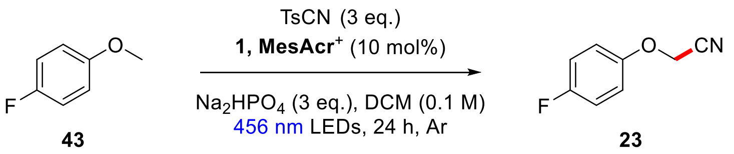

Synthesised according to **General procedure A** using tosyl cyanide (163 mg, 0.900 mmol) and 4-fluoroanisole (**43**) (34 µL, 0.300 mmol). Purified by column chromatography using 0 – 40% Et_2O in pentane as eluent which gave 2-(4-fluorophenoxy)acetonitrile (**23**) (31 mg, 0.205 mmol, 68%) as an orange oil. ^{1}H NMR (400 MHz, $CDCl_3$) δ 7.09 – 7.01 (m, 2H, 2 x ArH), 7.00 – 6.92 (m, 2H, 2 x ArH), 4.74 (s, 2H, CH_2); ^{13}C NMR (101 MHz, $CDCl_3$) δ 158.2 (d, $^1J_{C–F}$ = 241 Hz), 152.2, 116.2 (d, $^3J_{C–F}$ = 9.2 Hz), 115.9 (d, $^2J_{C–F}$ = 23.0 Hz), 114.4, 54.1; IR ν_{max}/cm^{-1} (neat): 3081 (w), 2919 (w), 2181 (vw), 1506 (vs), 1448 (m), 1199 (s), 1050 (m); *m/z* (EI): 151.1 ([M]$^+$,50), 111.0 (92), 83.0 (100). The NMR spectral data are in agreement with literature.[15]

2-(4-Chlorophenoxy)acetonitrile (**24**)[15]

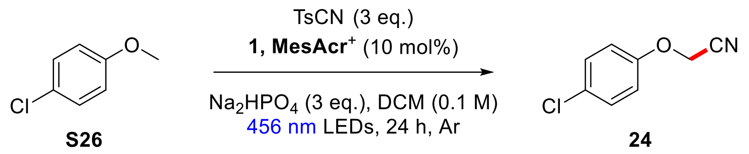

Synthesised according to **General procedure A** using tosyl cyanide (163 mg, 0.900 mmol) and 4-chloroanisole (**S26**) (37 µL, 0.300 mmol). Purified by column chromatography using 0 – 30% Et_2O in pentane as eluent which gave 2-(4-chlorophenoxy)acetonitrile (**24**) (44 mg, 0.263 mmol, 88%) as a pale orange solid. M.pt: 42 – 44 °C (lit: 46 – 47 °C)[15]; ^{1}H NMR (400 MHz, $CDCl_3$) δ 7.34 – 7.29 (m, 2H, 2 x ArH), 6.96 – 6.90 (m, 2H, 2 x ArH), 4.75 (s, 2H, CH_2); ^{13}C NMR (101 MHz, $CDCl_3$) δ 154.6, 129.4, 127.9, 115.9, 114.2, 53.4; IR ν_{max}/cm^{-1} (neat): 3100 (w), 3079 (w), 2927 (w), 2854 (w), 2256 (vw), 2091 (w), 1586 (m), 1493 (s), 1446 (m), 1208 (s), 830 (s); *m/z* (EI): 167.0 ([M]$^+$,50), 127.0 (100), 99.0 (92). The NMR spectral data are in agreement with literature.[15]

2-(4-Acetylphenoxy)acetonitrile (**25**)

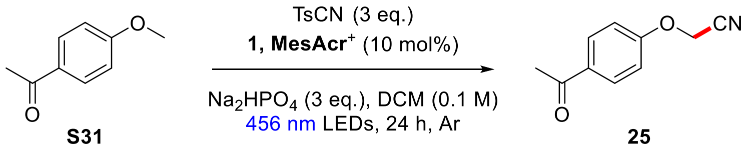

Synthesised according to **General procedure A** using tosyl cyanide (163 mg, 0.900 mmol) and 4-methoxyacetophenone (**S31**) (45 mg, 0.300 mmol). Purified by column chromatography using 0 – 100% Et_2O in hexane as eluent which gave 2-(4-acetylphenoxy)acetonitrile (**25**) (20 mg, 0.114 mmol, 38%) as an orange solid. M.pt: 79 – 81 °C; ^{1}H NMR (400 MHz, $CDCl_3$) δ 8.02 – 7.95 (m, 2H, 2 x ArH), 7.06 – 7.00 (m, 2H, 2 x ArH), 4.84 (s, 2H, CH_2), 2.58 (s, 3H, CH_3); ^{13}C NMR (101 MHz, $CDCl_3$) δ 196.0, 159.4, 131.9, 130.3, 114.0 (2 x C), 52.8, 25.9; IR ν_{max}/cm^{-1} (neat): 3075 (w), 3001 (w), 2956 (w), 2930 (w), 2262 (vw), 2074 (w), 1677 (s), 1603 (s), 1580 (s), 1275 (s), 1247 (vs), 1188 (s); HRMS (ESI) *m/z*: [*M*+H]$^+$ calcd for $C_{10}H_{10}NO_2^+$: 176.0706; found: 176.0708.

2-(3-Acetylphenoxy)acetonitrile (**26**)

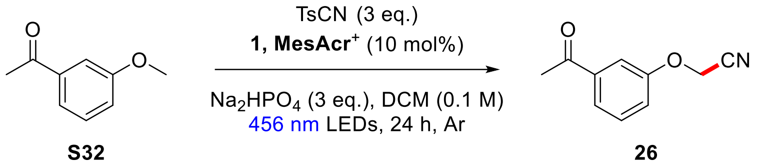

Synthesised according to **General procedure B** using tosyl cyanide (163 mg, 0.900 mmol) and 3-methoxyacetophenone (**S32**) (41 µL, 0.300 mmol). Purification was attempted. However, product material co-eluted with the inseparable by-product **A**. NMR yield of 42% was calculated as shown in Appendix 5. The ^{1}H NMR of the crude reaction product is shown in Appendix 4.

2-(Naphthalen-2-yloxy)acetonitrile (**27**)[16]

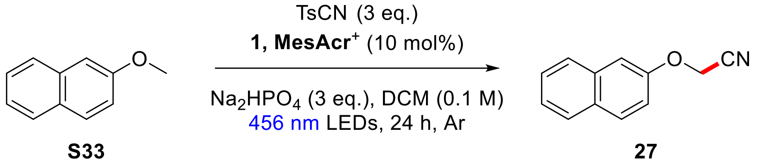

Synthesised according to **General procedure A** using tosyl cyanide (163 mg, 0.900 mmol) and 2-methoxynaphthalene (**S33**) (48 mg, 0.300 mmol). Purified by column chromatography using 0 – 50% Et_2O in hexane as eluent which gave 2-(naphthalen-2-yloxy)acetonitrile (**27**) (32 mg, 0.175 mmol, 58%) as a white solid. M.pt: 75 – 77 °C (lit: 74 – 76 °C)[16]; ^{1}H NMR (400 MHz, $CDCl_3$) δ 7.84 – 7.76 (m, 3H, 3 x ArH), 7.50 (ddd, *J* = 8.3, 7.0, 1.4 Hz, 1H, ArH), 7.42 (ddd, *J* = 8.3, 6.9, 1.3 Hz, 1H, ArH), 7.25 (d, *J* = 3.0 Hz, 1H, ArH), 7.19 (dd, *J* = 9.0, 2.5 Hz, 1H, ArH), 4.89 (s, 2H, CH_2); ^{13}C NMR (101 MHz, $CDCl_3$) δ 154.0, 133.5, 129.7, 129.4, 127.2, 126.6, 126.4, 124.3, 117.6, 114.5, 107.3, 53.0; IR ν_{max}/cm^{-1} (neat): 3061 (w), 2927 (w), 2852 (w), 2262 (vw), 2072 (w), 1633 (m), 1599 (m), 1446 (m), 1217 (s); *m/z* (EI): 183.1 ([M]$^{+}$,35), 143.1 (21), 115.1 (100), 89.1 (9). The NMR spectral data are in agreement with literature.[16]

Allylation Reactions and Products

((4-Phenoxybut-1-en-2-yl)sulfonyl)benzene (**28**)

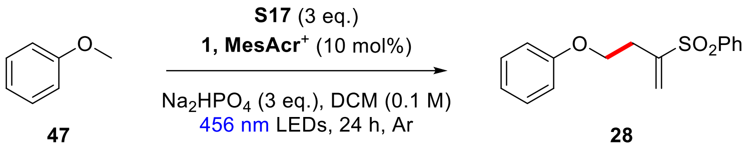

Synthesised according to **General procedure A** using (prop-2-ene-1,2-diyldisulfonyl)dibenzene (**S17**) (290 mg, 0.900 mmol) and anisole (**47**) (33 µL, 0.300 mmol). Purified by column chromatography using 0 – 30% EtOAc in hexane as eluent which gave ((4-phenoxybut-1-en-2-yl)sulfonyl)benzene (**28**) (29 mg, 0.101 mmol, 34%) as a brown oil. ^{1}H NMR (400 MHz, $CDCl_3$) δ 7.93 – 7.86 (m, 2H, 2 x ArH), 7.65 – 7.60 (m, 1H, ArH), 7.57 – 7.50 (m, 2H, 2 x ArH), 7.26 – 7.21 (m, 2H, 2 x ArH), 6.97 – 6.90 (m, 1H, ArH), 6.83 – 6.76 (m, 2H, 2

x ArH), 6.49 (d, J = 0.5 Hz, 1H, CH_2), 5.96 (d, J = 0.5 Hz, 1H, CH_2), 4.06 (t, J = 6.4 Hz, 2H, CH_2), 2.73 (td, J = 6.4, 0.8 Hz, 2H, CH_2); ^{13}C NMR (101 MHz, $CDCl_3$) δ 157.7, 146.4, 138.2, 133.1, 129.0, 128.8, 127.8, 125.3, 120.6, 114.0, 64.7, 29.0; IR ν_{max}/cm^{-1} (neat): 3062 (w), 2934 (w), 2882 (w), 1601 (m), 1498 (m), 1305 (s), 1243 (s), 1137 (s); HRMS (ESI) *m/z*: [*M*+H]$^+$ calcd for $C_{16}H_{17}O_3S^+$: 289.0893; found: 289.0894.

((4-Phenoxypent-1-en-2-yl)sulfonyl)benzene (**29**)[17]

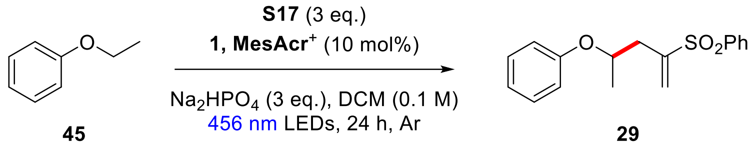

Synthesised according to **General procedure A** using (prop-2-ene-1,2-diyldisulfonyl)dibenzene (**S17**) (290 mg, 0.900 mmol) and phenetole (**45**) (38 µL, 0.300 mmol). Purified by column chromatography using 0 – 30% EtOAc in hexane as eluent which gave ((4-phenoxypent-1-en-2-yl)sulfonyl)benzene (**29**) (62 mg, 0.205 mmol, 68%) as a pale yellow oil. ^{1}H NMR (400 MHz, $CDCl_3$) δ 7.91 – 7.83 (m, 2H, 2 x ArH), 7.66 – 7.57 (m, 1H, ArH), 7.56 – 7.47 (m, 2H, 2 x ArH), 7.27 – 7.20 (m, 2H, 2 x ArH), 6.97 – 6.90 (m, 1H, ArH), 6.83 – 6.75 (m, 2H, 2 x ArH), 6.44 (s, 1H, CH_2), 5.93 (s, 1H, CH_2), 4.60 – 4.49 (m, 1H, CH), 2.66 (ddd, J = 15.6, 7.4, 0.8 Hz, 1H, CH_2), 2.50 (ddd, J = 15.3, 5.3, 1.0 Hz, 1H, CH_2), 1.26 (d, J = 6.0 Hz, 3H, CH_3); ^{13}C NMR (101 MHz, $CDCl_3$) δ 156.9, 146.2, 138.2, 133.1, 129.0, 128.8, 127.8, 126.3, 120.6, 115.3, 70.9, 36.2, 19.0; IR ν_{max}/cm^{-1} (neat): 3062 (w), 2979 (w), 2919 (w), 2850 (w), 1601 (m), 1491 (m), 1307 (s), 1234 (s), 1141 (s); *m/z* (EI): 302.1 ([M]$^+$,7), 209.1 (89), 143.0 (100), 125.0 (58), 94.0 (80), 77.0 (96). The NMR spectral data are in agreement with literature.[17]

((2-Methyl-4-(phenylsulfonyl)pent-4-en-2-yl)oxy)benzene (**30**)

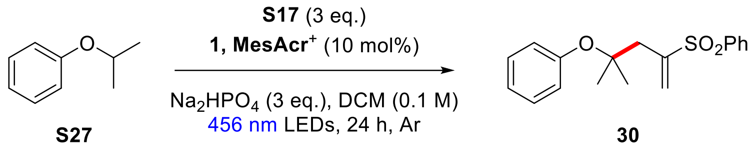

Synthesised according to **General procedure A** using (prop-2-ene-1,2-diyldisulfonyl)dibenzene (**S17**) (290 mg, 0.900 mmol) and isoproproxybenzene (**S27**) (44 µL, 0.300 mmol). Purified by column chromatography using 0 – 30% EtOAc in hexane as eluent which gave ((2-methyl-4-(phenylsulfonyl)pent-4-en-2-yl)oxy)benzene (**30**) (68 mg, 0.215 mmol, 72%) as a brown oil.

<u>1.00 mmol scale</u>

Synthesised according to **General procedure A** using a 20 ml microwave vial, (prop-2-ene-1,2-diyldisulfonyl)dibenzene (**S17**) (967 mg, 3.00 mmol), isoproproxybenzene (**S27**) (147 µL, 1.00 mmol), 3,6-di-*tert*-butyl-9-mesityl-*N*-phenylacridinium tetrafluoroborate (57 mg, 0.10 mmol), Na_2HPO_4 (426 mg, 3.00

mmol) and anhydrous DCM (10 mL). Purified by column chromatography using 0 – 30% EtOAc in hexane as eluent which gave ((2-methyl-4-(phenylsulfonyl)pent-4-en-2-yl)oxy)benzene (**30**) (267 mg, 0.87 mmol, 87%) as an orange oil.

^{1}H NMR (400 MHz, $CDCl_3$) δ 7.92 – 7.86 (m, 2H, 2 x ArH), 7.65 – 7.59 (m, 1H, ArH), 7.57 – 7.51 (m, 2H, 2 x ArH), 7.25 – 7.19 (m, 2H, 2 x ArH), 7.10 – 7.04 (m, 1H, ArH), 6.90 – 6.84 (m, 2H, 2 x ArH), 6.64 (s, 1H, CH_2), 6.47 (app. t, *J* = 1.4 Hz, 1H, CH_2), 2.63 (d, *J* = 0.5 Hz, 2H, CH_2), 1.21 (s, 6H, 2 x CH_3); ^{13}C NMR (101 MHz, $CDCl_3$) δ 154.0, 146.2, 138.5, 132.9, 128.7, 128.5, 127.9, 127.2, 123.29, 123.26, 79.1, 39.2, 25.8; IR ν_{max}/cm^{-1} (neat): 3062 (w), 2979 (w), 2936 (w), 1593 (m), 1489 (m), 1305 (s), 1227 (m), 1139 (s); HRMS (ESI) *m/z*: [*M*+H]$^+$ calcd for $C_{18}H_{21}O_3S^+$: 317.1206; found: 317.1206. Data for the 0.30 mmol and 1.00 mmol scale reactions are the same.

((3-(1-Phenoxycyclopentyl)prop-1-en-2-yl)sulfonyl)benzene (**31**)

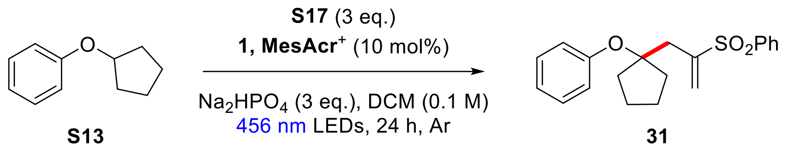

Synthesised according to **General procedure A** using (prop-2-ene-1,2-diyldisulfonyl)dibenzene (**S17**) (290 mg, 0.900 mmol) and (cyclopentyloxy)benzene (**S13**) (49 mg, 0.300 mmol). Purified by column chromatography using 0 – 30% EtOAc in hexane as eluent which gave ((3-(1-phenoxycyclopentyl)prop-1-en-2-yl)sulfonyl)benzene (**31**) (75 mg, 0.219 mmol, 73%) as an off-white solid. M.pt: 78 – 80 °C; ^{1}H NMR (400 MHz, $CDCl_3$) δ 7.87 – 7.80 (m, 2H, 2 x ArH), 7.66 – 7.59 (m, 1H, ArH), 7.56 – 7.49 (m, 2H, 2 x ArH), 7.19 – 7.11 (m, 2H, 2 x ArH), 6.99 – 6.92 (m, 1H, ArH), 6.79 – 6.73 (m, 2H, 2 x ArH), 6.58 (s, 1H, CH_2), 6.42 (app. t, *J* = 1.8 Hz, 1H, CH_2), 2.73 (app. t, *J* = 1.4 Hz, 2H, CH_2), 2.13 – 2.01 (m, 2H, CH_2), 1.82 – 1.68 (m, 2H, CH_2), 1.60 – 1.49 (m, 4H, 2 x CH_2); ^{13}C NMR (101 MHz, $CDCl_3$) δ 154.4, 146.1, 138.2, 132.9, 128.8, 128.7, 127.7, 126.3, 122.0, 120.3, 89.1, 37.4, 35.1, 23.4; IR ν_{max}/cm^{-1} (neat): 3111 (w), 2958 (w), 2930 (w), 2874 (w), 1588 (m), 1487 (m), 1303 (s), 1292 (s), 1163 (s); HRMS (ESI) *m/z*: [*M*+Na]$^+$ calcd for $C_{20}H_{22}NaO_3S^+$: 365.1182; found: 365.1182.

((4-Phenoxynon-1-en-2-yl)sulfonyl)benzene (**32**)

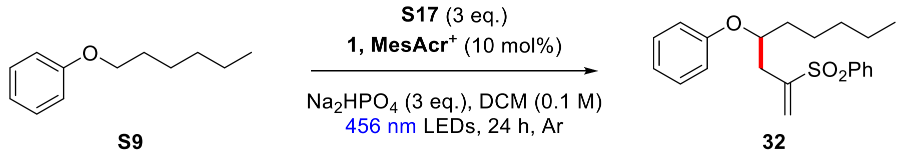

Synthesised according to **General procedure A** using (prop-2-ene-1,2-diyldisulfonyl)dibenzene (**S17**) (290 mg, 0.900 mmol) and (hexyloxy)benzene (**S9**) (54 mg, 0.300 mmol). Purified by column chromatography using 0 – 30% EtOAc in hexane as eluent which gave ((4-phenoxynon-1-en-2-yl)sulfonyl)benzene (**32**) (63 mg, 0.176 mmol, 59%) as an orange oil. ^{1}H NMR (400 MHz, $CDCl_3$) δ 7.88 – 7.82 (m, 2H, 2 x ArH), 7.65 –

7.58 (m, 1H, ArH), 7.55 – 7.48 (m, 2H, 2 x ArH), 7.26 – 7.21 (m, 2H, 2 x ArH), 6.96 – 6.89 (m, 1H, ArH), 6.84 – 6.77 (m, 2H, 2 x ArH), 6.41 (s, 1H, CH_2), 5.91 (s, 1H, CH_2), 4.44 – 4.34 (m, 1H, CH), 2.66 – 2.48 (m, 2H, CH_2), 1.64 – 1.48 (m, 2H, CH_2), 1.33 – 1.17 (m, 6H, 3 x CH_2), 0.86 (t, J = 7.0 Hz, 3H, CH_3); ^{13}C NMR (101 MHz, $CDCl_3$) δ 157.4, 146.2, 138.3, 133.0, 129.0, 128.7, 127.8, 126.5, 120.5, 115.3, 75.0, 34.0, 33.1, 31.2, 24.2, 22.0, 13.5; IR ν_{max}/cm^{-1} (neat): 2956 (w), 2923 (m), 2854 (w), 1601 (m), 1497 (m), 1307 (s), 1240 (s), 1141 (s); HRMS (ESI) *m/z*: [*M*+H]$^+$ calcd for $C_{21}H_{27}O_3S^+$: 359.1675; found: 359.1676.

(3-Phenoxy-5-(phenylsulfonyl)hex-5-en-1-yl)benzene (**33**)

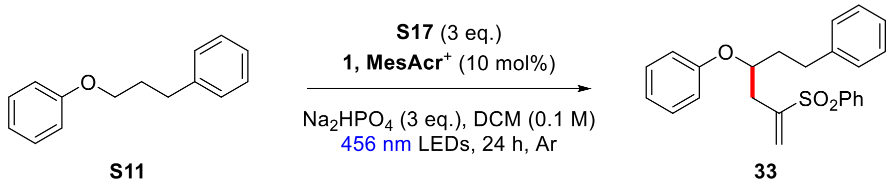

Synthesised according to **General procedure A** using (prop-2-ene-1,2-diyldisulfonyl)dibenzene (**S17**) (290 mg, 0.900 mmol) and 3-phenoxypropylbenzene (**S11**) (64 mg, 0.300 mmol). Purified by column chromatography using 0 – 30% EtOAc in hexane as eluent which gave (3-phenoxy-5-(phenylsulfonyl)hex-5-en-1-yl)benzene (**33**) (65 mg, 0.166 mmol, 55%) as an orange oil. ^{1}H NMR (400 MHz, $CDCl_3$) δ 7.86 – 7.80 (m, 2H, 2 x ArH), 7.64 – 7.57 (m, 1H, ArH), 7.53 – 7.46 (m, 2H, 2 x ArH), 7.27 – 7.17 (m, 5H, 5 x ArH), 7.13 – 7.07 (m, 2H, 2 x ArH), 6.97 – 6.89 (m, 1H, ArH), 6.81 – 6.74 (m, 2H, 2 x ArH), 6.41 (s, 1H, CH_2), 5.87 (s, 1H, CH_2), 4.50 – 4.39 (m, 1H, CH), 2.76 – 2.52 (m, 4H, 2 x CH_2), 1.98 – 1.82 (m, 2H, CH_2); ^{13}C NMR (101 MHz, $CDCl_3$) δ 157.2, 146.1, 140.8, 138.2, 133.1, 129.1, 128.8, 128.0, 127.9, 127.8, 126.6, 125.5, 120.6, 115.3, 74.1, 34.9, 33.9, 30.9; IR ν_{max}/cm^{-1} (neat): 3062 (w), 3029 (w), 2923 (w), 2854 (w), 1599 (m), 1497 (m), 1307 (s), 1234 (s), 1135 (s); HRMS (ESI) *m/z*: [*M*+H]$^+$ calcd for $C_{24}H_{25}O_3S^+$: 393.1519; found: 393.1520.

(1-Phenoxy-3-(phenylsulfonyl)but-3-en-1-yl)benzene (**34**)

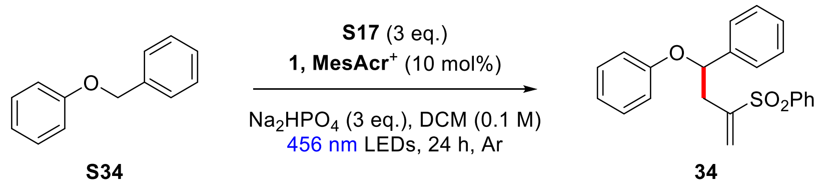

Synthesised according to **General procedure A** using (prop-2-ene-1,2-diyldisulfonyl)dibenzene (**S17**) (290 mg, 0.900 mmol) and benzyl phenyl ether (**S34**) (34 µL, 0.300 mmol). Purified by column chromatography using 0 – 30% EtOAc in hexane as eluent which gave (1-phenoxy-3-(phenylsulfonyl)but-3-en-1-yl)benzene (**34**) (71 mg, 0.195 mmol, 65%) as a yellow oil. ^{1}H NMR (400 MHz, $CDCl_3$) δ 7.91 – 7.85 (m, 2H, 2 x ArH), 7.64 – 7.58 (m, 1H, ArH), 7.54 – 7.48 (m, 2H, 2 x ArH), 7.33 – 7.22 (m, 5H, 5 x ArH), 7.18 – 7.12 (m, 2H, 2 x ArH), 6.90 – 6.84 (m, 1H, ArH), 6.75 – 6.69 (m, 2H, 2 x ArH), 6.43 (s, 1H, CH_2), 5.84 (s, 1H, CH_2), 5.21 (dd, J = 8.9, 4.4 Hz, 1H, CH), 2.89 (ddd, J = 15.6, 9.0, 0.8 Hz, 1H, CH_2), 2.71 (ddd, J = 15.6, 4.4, 1.0 Hz, 1H, CH_2); ^{13}C NMR (101 MHz, $CDCl_3$) δ 157.1, 145.5, 139.9, 138.3, 133.1, 128.83, 128.79, 128.2, 127.8, 127.5,

126.9, 125.5, 120.6, 115.3, 77.1, 38.4; IR ν_{max}/cm^{-1} (neat): 3066 (w), 3034 (w), 2941 (w), 1601 (m), 1495 (m), 1305 (s), 1240 (s), 1135 (s); HRMS (ESI) *m/z*: $[M+Na]^+$ calcd for $C_{22}H_{20}NaO_3S^+$: 387.1025; found: 387.1024.

2-(3-Phenoxy-5-(phenylsulfonyl)hex-5-en-1-yl)isoindoline-1,3-dione (**35**)

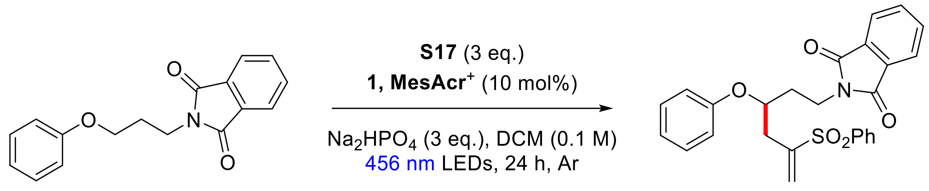

S15 **35**

Synthesised according to **General procedure A** using (prop-2-ene-1,2-diyldisulfonyl)dibenzene (**S17**) (290 mg, 0.900 mmol) and 2-(3-phenoxypropyl)isoindoline-1,3-dione (**S15**) (85 mg, 0.300 mmol). Purified by column chromatography using 0 – 50% EtOAc in hexane as eluent which gave 2-(3-phenoxy-5-(phenylsulfonyl)hex-5-en-1-yl)isoindoline-1,3-dione (**35**) (101 mg, 0.219 mmol, 73%) as a brown gum.

1.00 mmol scale

Synthesised according to **General procedure A** using a 20 ml microwave vial, (prop-2-ene-1,2-diyldisulfonyl)dibenzene (**S17**) (967 mg, 3.00 mmol), 2-(3-phenoxypropyl)isoindoline-1,3-dione (**S15**) (281 mg, 1.00 mmol), 3,6-di-*tert*-butyl-9-mesityl-*N*-phenylacridinium tetrafluoroborate (57 mg, 0.10 mmol), Na_2HPO_4 (426 mg, 3.00 mmol) and anhydrous DCM (10 mL). Purified by column chromatography using 0 – 50% EtOAc in hexane as eluent which gave 2-(3-phenoxy-5-(phenylsulfonyl)hex-5-en-1-yl)isoindoline-1,3-dione (**35**) (349 mg, 0.76 mmol, 76%) as an orange gum.

^{1}H NMR (400 MHz, $CDCl_3$) δ 7.86 – 7.78 (m, 4H, 4 x ArH), 7.73 – 7.66 (m, 2H, 2 x ArH), 7.62 – 7.54 (m, 1H, ArH), 7.53 – 7.45 (m, 2H, 2 x ArH), 7.25 – 7.16 (m, 2H, 2 x ArH), 6.95 – 6.88 (m, 1H, ArH), 6.84 – 6.76 (m, 2H, 2 x ArH), 6.44 (s, 1H, CH_2), 5.94 (s, 1H, CH_2), 4.67 – 4.57 (m, 1H, CH), 3.86 – 3.71 (m, 2H, CH_2), 2.72 (ddd, *J* = 15.1, 6.1, 0.9 Hz, 1H, CH_2), 2.53 (ddd, *J* = 15.3, 6.7, 0.7 Hz, 1H, CH_2), 2.08 – 1.91 (m, 2H, CH_2); ^{13}C NMR (101 MHz, $CDCl_3$) δ 167.7, 156.7, 145.6, 138.0, 133.4, 133.1, 131.6, 129.1, 128.8, 127.8, 127.1, 122.7, 120.9, 115.5, 73.1, 33.9, 33.6, 31.5; IR ν_{max}/cm^{-1} (neat): 3102 (w), 3064 (w), 2927 (w), 1774 (m), 1709 (s), 1599 (m), 1305 (m), 1137 (s); HRMS (ESI) *m/z*: $[M+H]^+$ calcd for $C_{26}H_{24}NO_5S^+$: 462.1370; found: 462.1396. Data for the 0.30 mmol and 1.00 mmol scale reactions are the same.

1-Methyl-3-((3-(phenylsulfonyl)but-3-en-1-yl)oxy)benzene (**36**)

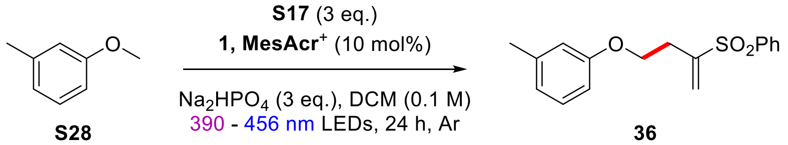

S28 **36**

Synthesised according to **General procedure A** using (prop-2-ene-1,2-diyldisulfonyl)dibenzene (**S17**) (290 mg, 0.900 mmol) and 3-methylanisole (**S28**) (38 µL, 0.300 mmol). Purified by column chromatography using 0 – 50% EtOAc in hexane as eluent which gave 1-methyl-3-((3-(phenylsulfonyl)but-3-en-1-yl)oxy)benzene (**36**) (20 mg, 0.066 mmol, 22%) as an orange gum. ^{1}H NMR (400 MHz, $CDCl_3$) δ 7.96 – 7.89 (m, 2H, 2 x ArH), 7.68 – 7.62 (m, 1H, ArH), 7.60 – 7.53 (m, 2H, 2 x ArH), 7.15 (t, *J* = 7.8 Hz, 1H, ArH), 6.81 – 6.76 (m, 1H, ArH), 6.67 – 6.58 (m, 2H, 2 x ArH), 6.51 (d, *J* = 0.5 Hz, 1H, CH_2), 5.99 (d, *J* = 0.5 Hz, 1H, CH_2), 4.07 (t, *J* = 6.4 Hz, 2H, CH_2), 2.74 (app. td, *J* = 6.3, 0.8 Hz, 2H, CH_2), 2.33 (s, 3H, CH_3); ^{13}C NMR (101 MHz, $CDCl_3$) δ 157.7, 146.5, 139.1, 138.2, 133.1, 128.8, 128.7, 127.8, 125.3, 121.4, 114.8, 110.8, 64.7, 29.0, 21.0; IR ν_{max}/cm^{-1} (neat): 3062 (w), 2921 (w), 1605 (m), 1493 (m), 1307 (s), 1260 (s), 1139 (s); HRMS (ESI) *m/z*: [*M*+H]$^+$ calcd for $C_{17}H_{19}O_3S^+$: 303.1049; found: 303.1050.

390 nm LEDs

Synthesised according to **General procedure B** using purple LEDs (390 nm), (prop-2-ene-1,2-diyldisulfonyl)dibenzene (**S17**) (290 mg, 0.900 mmol) and 3-methylanisole (**S28**) (38 µL, 0.300 mmol). NMR yield of 28% was calculated as shown in Appendix 5.

1-(*tert*-Butyl)-4-((3-(phenylsulfonyl)but-3-en-1-yl)oxy)benzene (**37**) and (4-(4-(*tert*-butyl)phenoxy)hepta-1,6-diene-2,6-diyldisulfonyl)dibenzene (**38**)

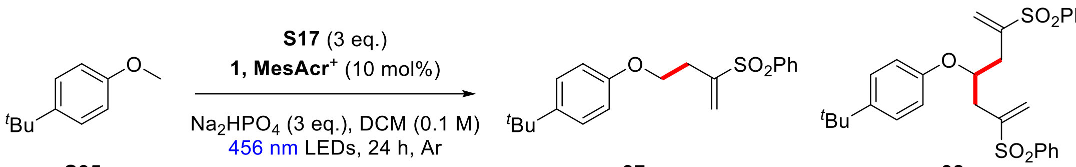

Synthesised according to **General procedure A** using (prop-2-ene-1,2-diyldisulfonyl)dibenzene (**S17**) (290 mg, 0.900 mmol) and 4-*tert*-butylanisole (**S35**) (53 µL, 0.300 mmol). Purified by column chromatography using 0 – 50% EtOAc in hexane as eluent which gave 1-(*tert*-butyl)-4-((3-(phenylsulfonyl)but-3-en-1-yl)oxy)benzene (**37**) (11 mg, 0.032 mmol, 11%) as a brown gum as well as the diallylated product, (4-(4-(*tert*-butyl)phenoxy)hepta-1,6-diene-2,6-diyldisulfonyl)dibenzene (**38**) (24 mg, 0.046 mmol, 15%) as an orange gum.

1-(*tert*-Butyl)-4-((3-(phenylsulfonyl)but-3-en-1-yl)oxy)benzene (**37**) : ^{1}H NMR (400 MHz, $CDCl_3$) δ 7.92 – 7.87 (m, 2H, 2 x ArH), 7.65 – 7.60 (m, 1H, ArH), 7.56 – 7.51 (m, 2H, 2 x ArH), 7.29 – 7.24 (m, 2H, 2 x ArH), 6.75 – 6.71 (m, 2H, 2 x ArH), 6.48 (d, *J* = 0.5 Hz, 1H, CH_2), 5.96 (d, *J* = 0.8 Hz, 1H, CH_2), 4.05 (t, *J* = 6.3 Hz, 2H, CH_2), 2.71 (app. td, *J* = 6.3, 0.6 Hz, 2H, CH_2), 1.29 (s, 9H, 3 x CH_3); ^{13}C NMR (101 MHz, $CDCl_3$) δ 155.4, 146.5, 143.3, 138.2, 133.1, 128.8, 127.8, 125.8, 125.2, 113.5, 64.8, 33.6, 31.0, 29.0; IR ν_{max}/cm^{-1} (neat): 3062 (w), 2960 (m), 2871 (w), 1612 (w), 1515 (s), 1450 (m), 1307 (s), 1245 (s), 1139 (s); HRMS (ESI) *m/z*: [*M*+H]$^+$ calcd for $C_{20}H_{25}O_3S^+$: 345.1519; found: 345.1520.

(4-(4-(*tert*-Butyl)phenoxy)hepta-1,6-diene-2,6-diyldisulfonyl)dibenzene (**38**) : ^{1}H NMR (400 MHz, $CDCl_3$) δ 7.83 – 7.78 (m, 4H, 4 x ArH), 7.64 – 7.59 (m, 2H, 2 x ArH), 7.53 – 7.48 (m, 4H, 4 x ArH), 7.25 – 7.21 (m, 2H, 2 x ArH), 6.71 – 6.67 (m, 2H, 2 x ArH), 6.42 (s, 2H, CH_2), 5.91 (d, J = 0.5 Hz, 2H, CH_2), 4.72 – 4.62 (m, 1H, CH), 2.57 – 2.43 (m, 4H, 2 x CH_2), 1.29 (s, 9H, 3 x CH_3); ^{13}C NMR (101 MHz, $CDCl_3$) δ 154.2, 145.5, 144.0, 137.9, 133.2, 128.8, 127.8, 126.6, 125.9, 114.9, 73.2, 33.6, 33.3, 31.0; IR ν_{max}/cm^{-1} (neat): 3064 (w), 2962 (w), 2908 (w), 2869 (w), 1610 (w), 1513 (m), 1307 (s), 1240 (s), 1141 (s); HRMS (ESI) *m/z*: [*M*+H]$^+$ calcd for $C_{29}H_{33}O_5S_2^+$: 525.1764; found: 525.1765.

1-Fluoro-4-((3-(phenylsulfonyl)but-3-en-1-yl)oxy)benzene (**39**)

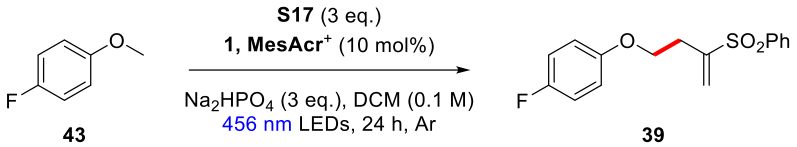

Synthesised according to **General procedure A** using (prop-2-ene-1,2-diyldisulfonyl)dibenzene (**S17**) (290 mg, 0.900 mmol) and 4-fluoroanisole (**43**) (34 µL, 0.300 mmol). Purified by column chromatography using 0 – 30% EtOAc in hexane as eluent which gave 1-fluoro-4-((3-(phenylsulfonyl)but-3-en-1-yl)oxy)benzene (**39**) (42 mg, 0.137 mmol, 46%) as an orange gum. ^{1}H NMR (400 MHz, $CDCl_3$) δ 7.93 – 7.86 (m, 2H, 2 x ArH), 7.66 – 7.59 (m, 1H, ArH), 7.57 – 7.50 (m, 2H, 2 x ArH), 6.99 – 6.88 (m, 2H, 2 x ArH), 6.78 – 6.69 (m, 2H, 2 x ArH), 6.48 (d, J = 0.5 Hz, 1H, CH_2), 5.95 (d, J = 0.5 Hz, 1H, CH_2), 4.03 (t, J = 6.4 Hz, 2H, CH_2), 2.71 (app. td, J = 6.4, 0.8 Hz, 2H, CH_2); ^{13}C NMR (101 MHz, $CDCl_3$) δ 156.9 (d, $^1J_{C\text{–}F}$ = 137 Hz), 153.8, 146.3, 138.1, 133.1, 128.8, 127.8, 125.4, 115.3 (d, $^2J_{C\text{–}F}$ = 23.0 Hz), 115.0 (d, $^3J_{C\text{–}F}$ = 7.7 Hz), 65.4, 29.1; IR ν_{max}/cm^{-1} (neat): 3100 (w), 3072 (w), 2947 (m), 1603 (w), 1508 (s), 1305 (s), 1204 (s), 1135 (s), 1061 (m); HRMS (ESI) *m/z*: [*M*+H]$^+$ calcd for $C_{16}H_{16}FO_3S^+$: 307.0799; found: 307.0799.

1-Chloro-4-((3-(phenylsulfonyl)but-3-en-1-yl)oxy)benzene (**40**)

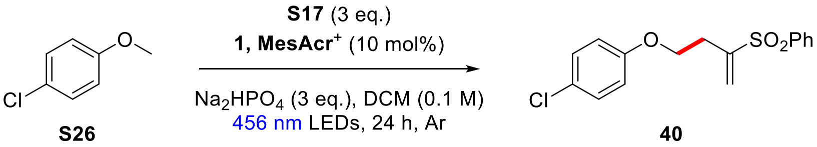

Synthesised according to **General procedure A** using (prop-2-ene-1,2-diyldisulfonyl)dibenzene (**S17**) (290 mg, 0.900 mmol) and 4-chloroanisole (**S26**) (37 µL, 0.300 mmol). Purified by column chromatography using 0 – 50% EtOAc in hexane as eluent which gave 1-chloro-4-((3-(phenylsulfonyl)but-3-en-1-yl)oxy)benzene (**40**) (34 mg, 0.105 mmol, 35%) as a pale yellow oil. ^{1}H NMR (400 MHz, $CDCl_3$) δ 7.93 – 7.85 (m, 2H, 2 x ArH), 7.66 – 7.59 (m, 1H, ArH), 7.57 – 7.50 (m, 2H, 2 x ArH), 7.22 – 7.15 (m, 2H, 2 x ArH), 6.74 – 6.68 (m, 2H, 2 x ArH), 6.47 (s, 1H, CH_2), 5.94 (d, J = 0.8 Hz, 1H, CH_2), 4.03 (t, J = 6.5 Hz, 2H, CH_2), 2.71 (app. td, J = 6.4, 0.8 Hz, 2H, CH_2); ^{13}C NMR (101 MHz, $CDCl_3$) δ 156.3, 146.2, 138.1, 133.2, 128.9, 128.8, 127.8,

125.55, 125.50, 115.3, 65.1, 29.0; IR ν_{max}/cm^{-1} (neat): 3068 (w), 2936 (w), 1599 (w), 1493 (s), 1307 (s), 1243 (s), 1137 (s), 826 (s); HRMS (ESI) *m/z*: [*M*+H]$^+$ calcd for $C_{16}H_{16}ClO_3S^+$: 323.0503; found: 323.0505.

Ethyl 4-methyl-2-methylene-4-phenoxypentanoate (**41**)

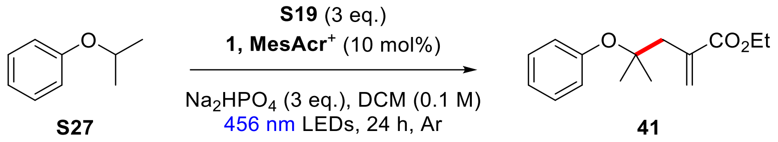

Synthesised according to **General procedure A** using ethyl 2-((phenylsulfonyl)methyl)acrylate (**S19**) (229 mg, 0.900 mmol) and isoproproxybenzene (**S27**) (44 µL, 0.300 mmol). Purified by column chromatography using 0 – 10% EtOAc in hexane as eluent which gave ethyl 4-methyl-2-methylene-4-phenoxypentanoate (**41**) (45 mg, 0.181 mmol, 60%) as a yellow oil. ^{1}H NMR (400 MHz, $CDCl_3$) δ 7.28 – 7.21 (m, 2H, 2 x ArH), 7.09 – 7.03 (m, 1H, ArH), 7.00 – 6.94 (m, 2H, 2 x ArH), 6.29 (d, *J* = 1.8 Hz, 1H, CH_2), 5.71 (m, 1H, CH_2), 4.21 (q, *J* = 7.3 Hz, 2H, CH_2), 2.77 (d, *J* = 0.5 Hz, 2H, CH_2), 1.29 (t, *J* = 7.0 Hz, 3H, CH_3), 1.26 (s, 6H, 2 x CH_3); ^{13}C NMR (101 MHz, $CDCl_3$) δ 167.5, 154.7, 137.1, 128.4, 127.6, 123.4, 122.8, 79.6, 60.3, 42.8, 25.7, 13.7; IR ν_{max}/cm^{-1} (neat): 3064 (w), 2980 (m), 2936 (w), 1716 (s), 1595 (m), 1491 (m), 1223 (s), 1154 (vs); HRMS (ESI) *m/z*: [*M*+H]$^+$ calcd for $C_{15}H_{21}O_3^+$: 249.1485; found: 249.1485.

4-Methyl-2-methylene-4-phenoxy-1-phenylpentan-1-one (**42**)

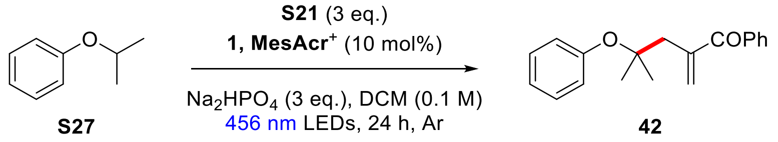

Synthesised according to **General procedure A** using 1-phenyl-2-((phenylsulfonyl)methyl)prop-2-en-1-one (**S21**) (258 mg, 0.900 mmol) and isoproproxybenzene (**S27**) (44 µL, 0.300 mmol). Purified by column chromatography using 0 – 25% EtOAc in hexane as eluent which gave 4-methyl-2-methylene-4-phenoxy-1-phenylpentan-1-one (**42**) (58 mg, 0.207 mmol, 69%) as a brown oil. ^{1}H NMR (400 MHz, $CDCl_3$) δ 7.87 – 7.83 (m, 2H, 2 x ArH), 7.56 – 7.51 (m, 1H, ArH), 7.47 – 7.42 (m, 2H, 2 x ArH), 7.25 – 7.20 (m, 2H, 2 x ArH), 7.07 – 7.00 (m, 1H, ArH), 6.96 – 6.89 (m, 2H, 2 x ArH), 5.96 (d, *J* = 1.0 Hz, 1H, CH_2), 5.73 (d, *J* = 1.3 Hz, 1H, CH_2), 2.96 (s, 2H, CH_2), 1.32 (s, 6H, 2 x CH_3); ^{13}C NMR (101 MHz, $CDCl_3$) δ 197.4, 154.6, 144.6, 136.9, 131.8, 129.3, 128.4, 127.9, 127.7, 123.2, 122.7, 79.5, 44.3, 26.0; IR ν_{max}/cm^{-1} (neat): 3062 (w), 2979 (w), 2934 (w), 1660 (s), 1595 (m), 1491 (m), 1219 (s), 1117 (s); HRMS (ESI) *m/z*: [*M*+H]$^+$ calcd for $C_{19}H_{21}O_2^+$: 281.1536; found: 281.1536.

Unsuccessful allylic sulfone substrates

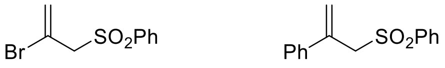

Further Functionalisation of Allylation Products 30 and 35

1-Fluoro-4-((5-methyl-5-phenoxy-3-(phenylsulfonyl)hexyl)oxy)benzene (**44**)

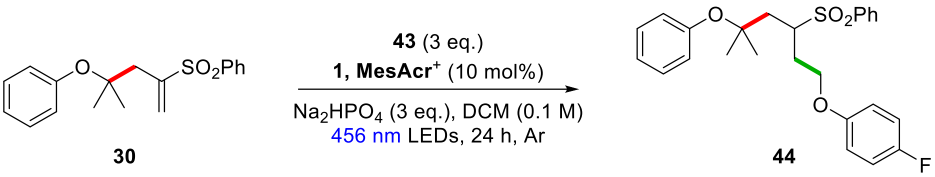

Synthesised according to **General procedure A** using ((2-methyl-4-(phenylsulfonyl)pent-4-en-2-yl)oxy)benzene (**30**) (95 mg, 0.300 mmol) and 4-fluoroanisole (101 µl, 0.900 mmol). Purified by column chromatography using 0 – 30% Et_2O in hexane as eluent which gave 1-fluoro-4-((5-methyl-5-phenoxy-3-(phenylsulfonyl)hexyl)oxy)benzene (**44**) (36 mg, 0.081 mmol, 27%) as an orange oil. ^{1}H NMR (400 MHz, $CDCl_3$) δ 7.96 – 7.90 (m, 2H, 2 x ArH), 7.68 – 7.61 (m, 1H, ArH), 7.59 – 7.53 (m, 2H, 2 x ArH), 7.24 – 7.17 (m, 2H, 2 x ArH), 7.09 – 7.03 (m, 1H, ArH), 6.92 – 6.80 (m, 4H, 4 x ArH), 6.76 – 6.69 (m, 2H, 2 x ArH), 4.22 – 4.08 (m, 2H, CH_2), 3.69 (m, 1H, CH), 2.50 – 2.37 (m, 2H, CH_2), 2.35 (dd, *J* = 14.8, 2.3 Hz, 1H, CH_2), 1.98 (dd, *J* = 14.8, 7.5 Hz, 1H, CH_2), 1.27 (s, 3H, CH_3), 1.17 (s, 3H, CH_3); ^{13}C NMR (101 MHz, $CDCl_3$) δ 156.7 (d, $^1J_{C–F}$ = 236 Hz), 154.2, 153.9, 137.3, 133.2, 128.7, 128.5, 128.4, 123.4, 123.2, 115.2 (d, $^2J_{C–F}$ = 23.0 Hz), 114.9 (d, $^3J_{C–F}$ = 7.7 Hz), 78.8, 65.2, 57.6, 41.1, 30.0, 27.1, 24.6; IR ν_{max}/cm^{-1} (neat): 3064 (w), 2979 (w), 2934 (w), 1593 (m), 1508 (vs), 1305 (s), 1206 (s), 1141 (s), 1087 (s); HRMS (ESI) *m/z*: $[M+H]^+$ calcd for $C_{34}H_{34}NO_6S^+$: 584.2101; found: 584.2104.

2-(3,7-Diphenoxy-5-(phenylsulfonyl)octyl)isoindoline-1,3-dione (**46**)*

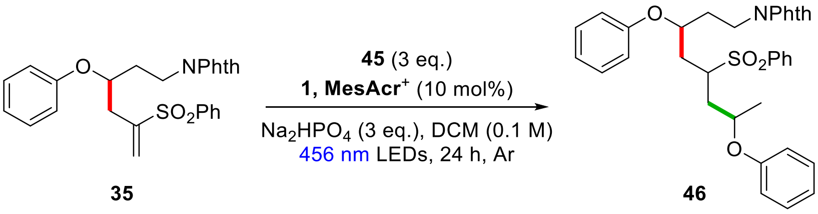

Synthesised according to **General procedure A** using 2-(3-phenoxy-5-(phenylsulfonyl)hex-5-en-1-yl)isoindoline-1,3-dione (**35**) (138 mg, 0.300 mmol) and phenetole (**45**) (114 µl, 0.900 mmol). Purified by column chromatography using 0 – 30% Et_2O in hexane as eluent which gave 40% EtOAc in hexane as eluent which gave a complex mixture of diastereomers of 2-(3,7-diphenoxy-5-(phenylsulfonyl)octyl)isoindoline-1,3-dione (**46**) (134 mg, 0.230 mmol, 77%) as a brown oil. ^{1}H NMR (400 MHz, $CDCl_3$) δ 7.87 – 7.78 (m, 4H, 4 x ArH), 7.74 – 7.68 (m, 2H, 2 x ArH), 7.65 – 7.58 (m, 1H, ArH), 7.54 – 7.46 (m, 2H, 2 x ArH), 7.27 – 7.18 (m, 4H, 4 x ArH), 6.98 – 6.90 (m, 2H, 2 x ArH), 6.85 – 6.77 (m, 4H, 4 x ArH), 4.75 – 4.65 (m, 1H, CH), 4.60

– 4.50 (m, 1H, CH), 3.83 – 3.68 (m, 2H, CH_2), 3.47 (app. q, J = 5.9 Hz, 1H, CH), 2.33 – 1.90 (m, 6H, 3 x CH_2), 1.20 (d, J = 6.0 Hz, 3H, CH_3); ^{13}C NMR (101 MHz, $CDCl_3$) δ 167.6, 157.00, 156.96, 137.1, 133.4, 133.3, 131.6, 129.1, 129.0, 128.8, 128.2, 122.7, 120.9, 120.5, 115.5 (2 x C), 73.3, 71.2, 57.7, 36.3, 33.6, 33.5, 31.6, 19.1; IR ν_{max}/cm^{-1} (neat): 3062 (w), 2975 (w), 2932 (w), 1774 (m), 1711 (s), 1599 (m), 1493 (m), 1305 (m), 1232 (m), 1143 (s); HRMS (ESI) *m/z*: $[M+H]^+$ calcd for $C_{25}H_{27}FO_4SNa^+$: 465.1506; found: 465.1506.

* During purification 3 separate samples of product material containing what appeared to be varying ratios of different diastereomers of **46** were recovered with a combined mass of 134 mg. For clarity, the data reported above is taken from the least complex sample, however, ^{1}H and ^{13}C NMR spectra (Appendix 2) and LCMS data (Appendix 3) for each of the other 2 samples is included.

Appendix 1: ^{1}H and ^{13}C NMR Spectra

Catalyst Synthesis

3,3'-Oxybis(*tert*-butylbenzene) (**S3**)

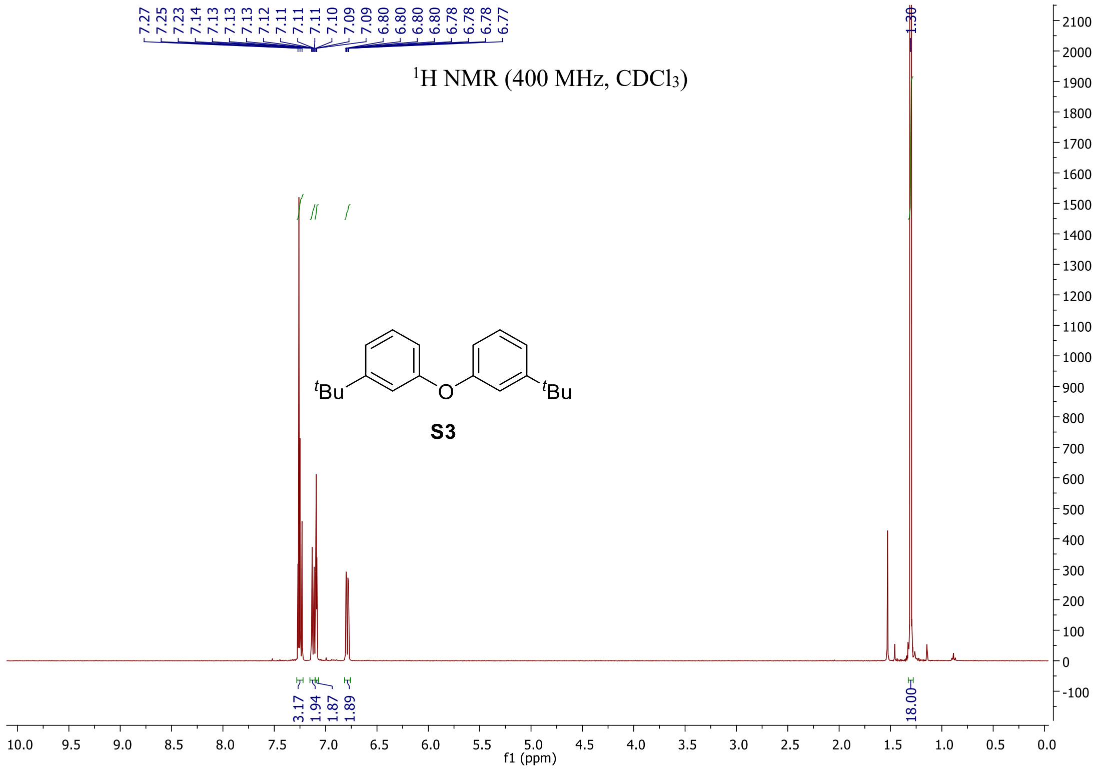

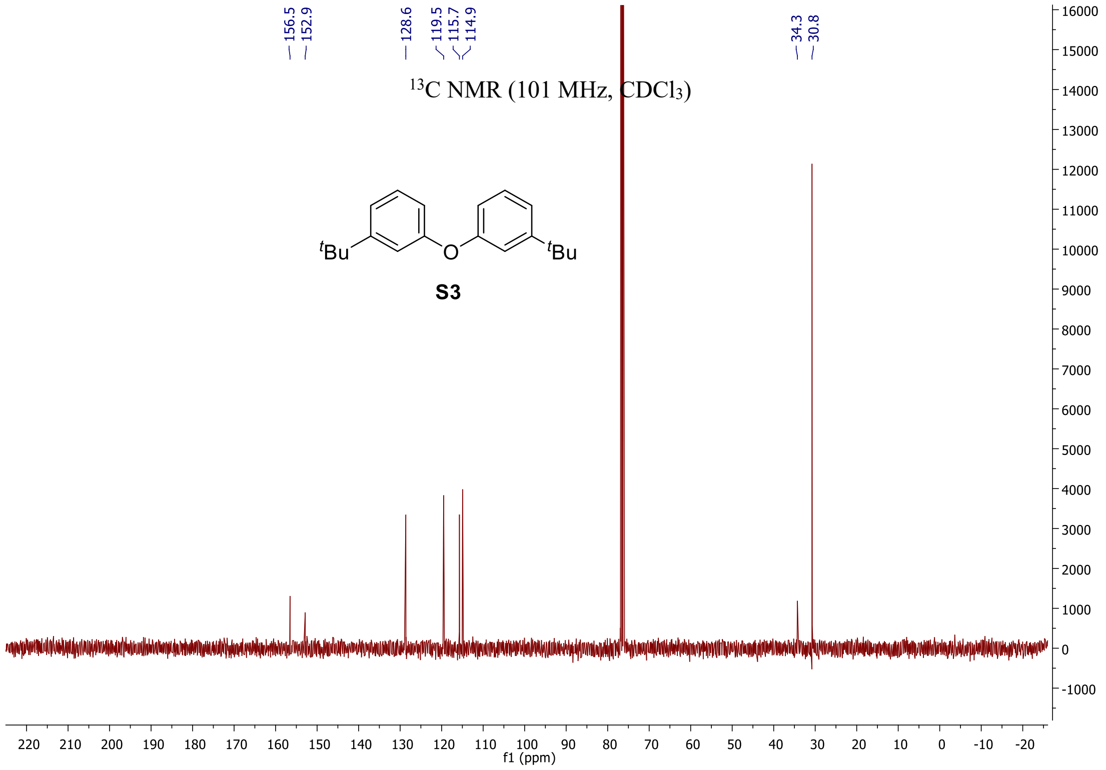

Methyl 2,4,6-trimethylbenzoate (**S5**)

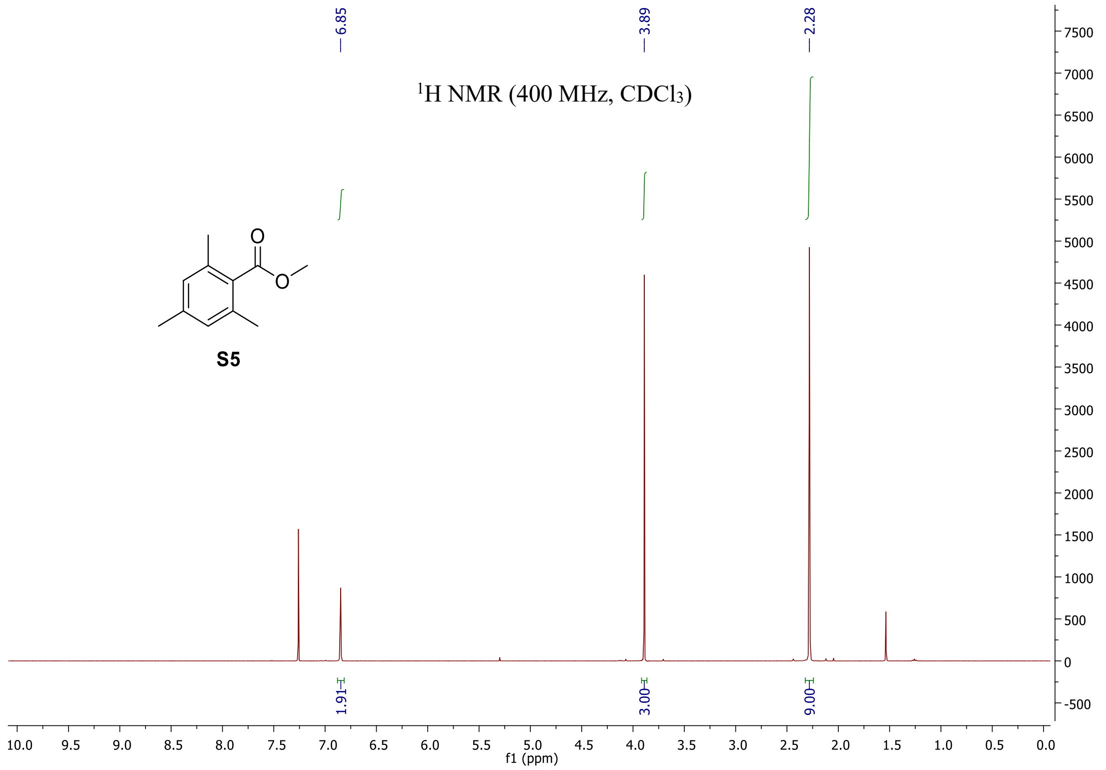

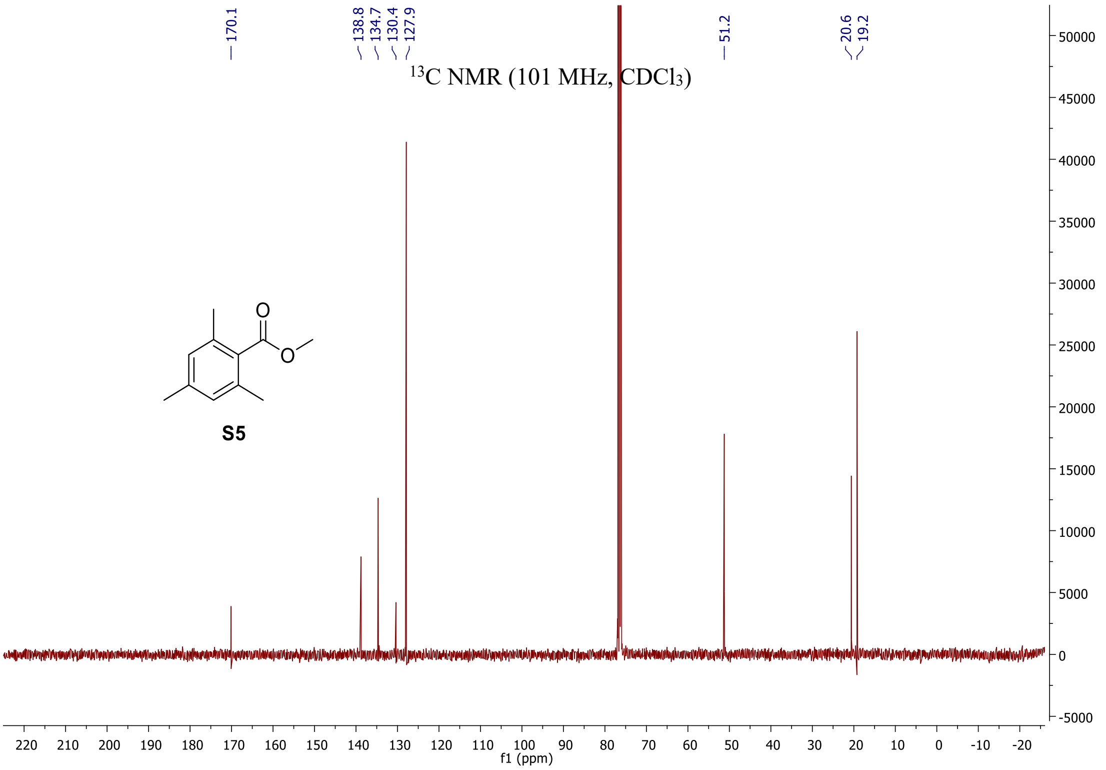

3,6-Di-*tert*-butyl-9-mesitylxanthylium tetrafluoroborate (**S6**)

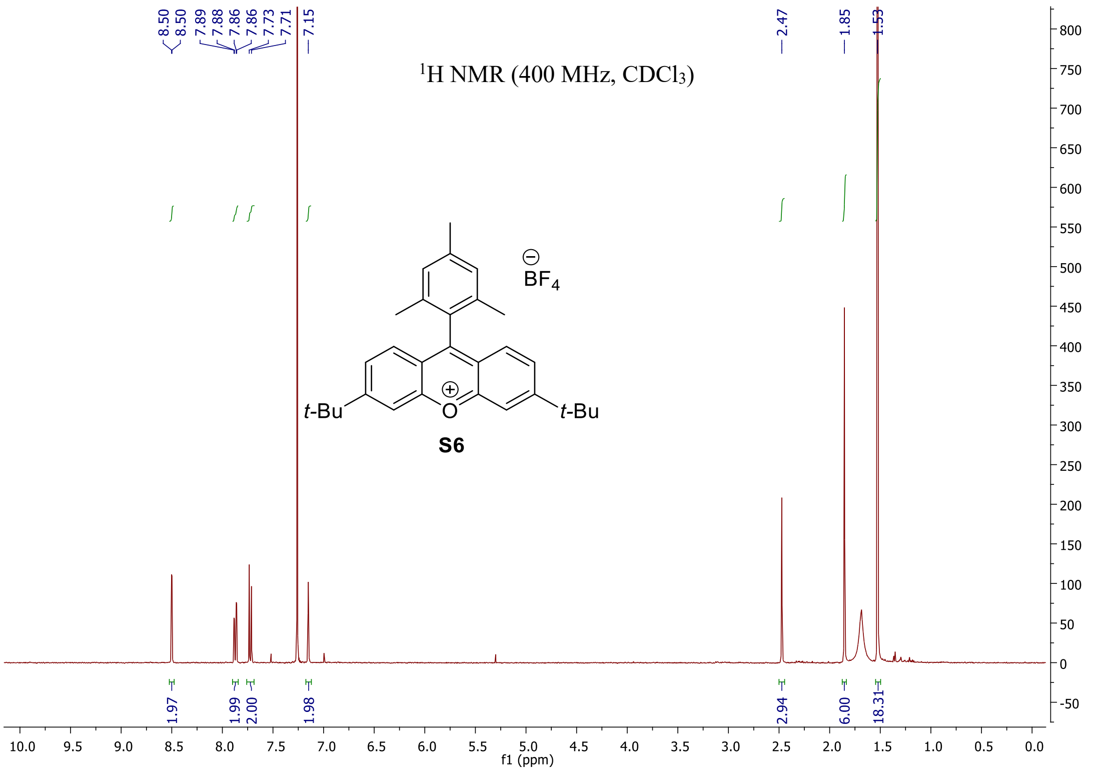

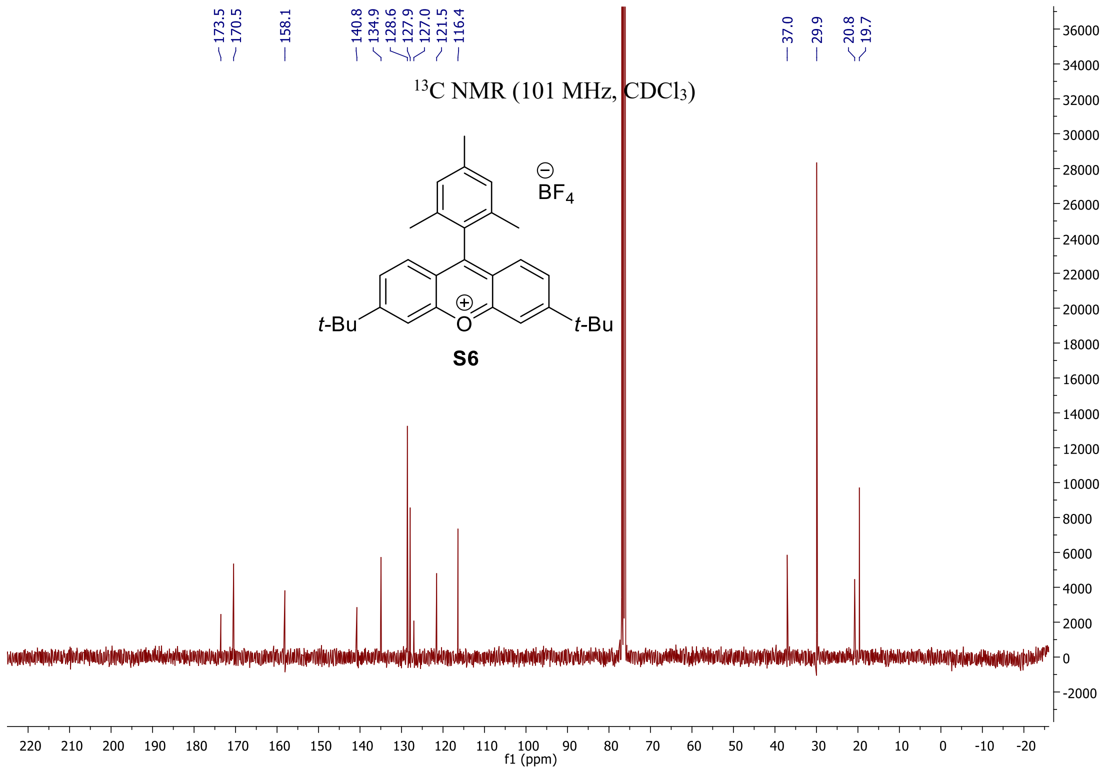

3,6-Di-*tert*-butyl-9-mesityl-*N*-phenylacridinium tetrafluoroborate (**1**, **MesAcr**$^+$)

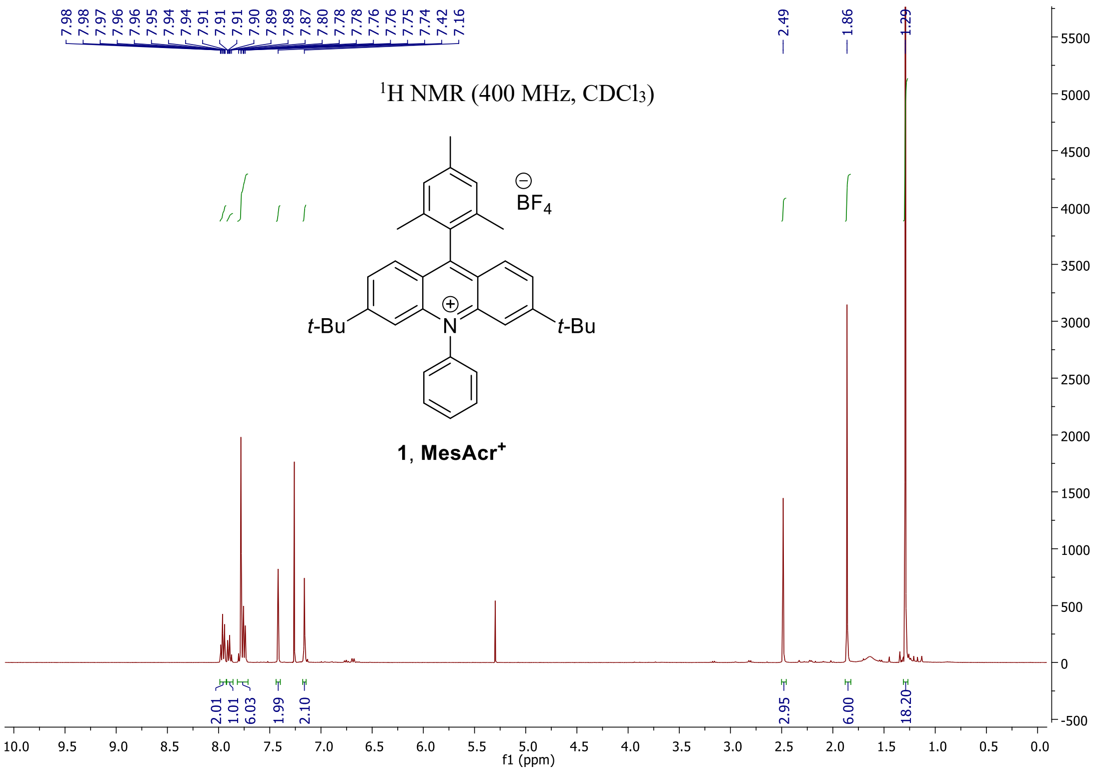

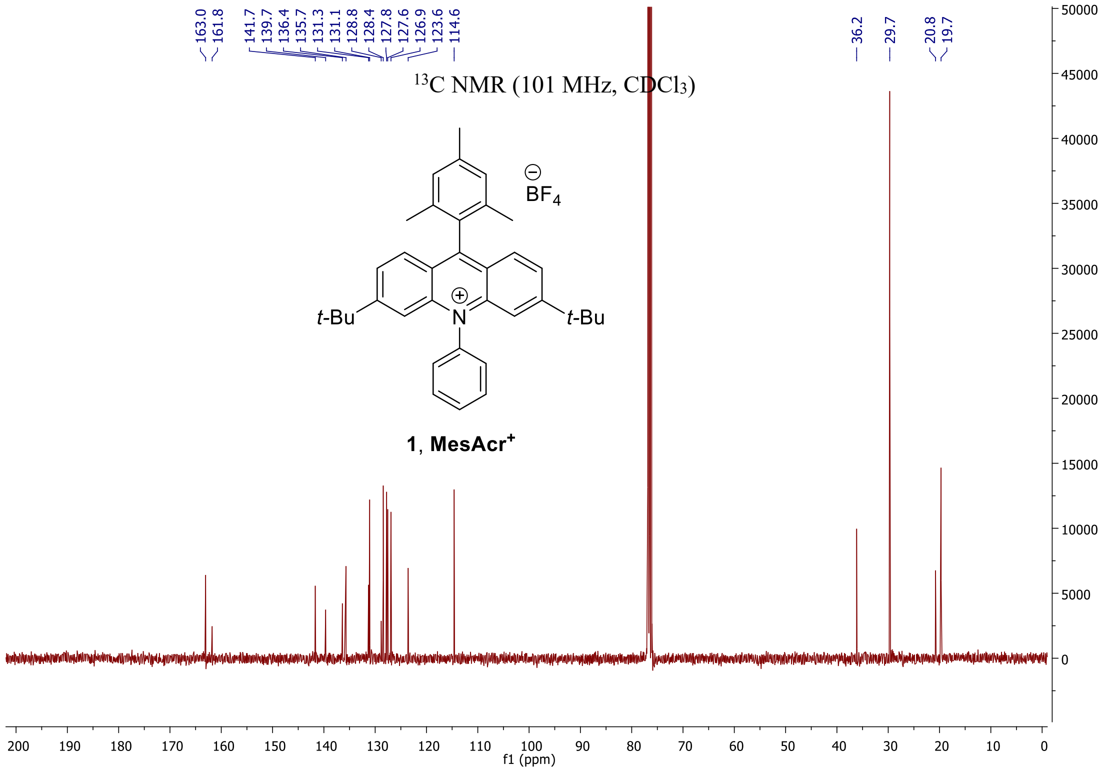

Aryl Alkyl Ether Substrates

(Hexyloxy)benzene (**S9**)

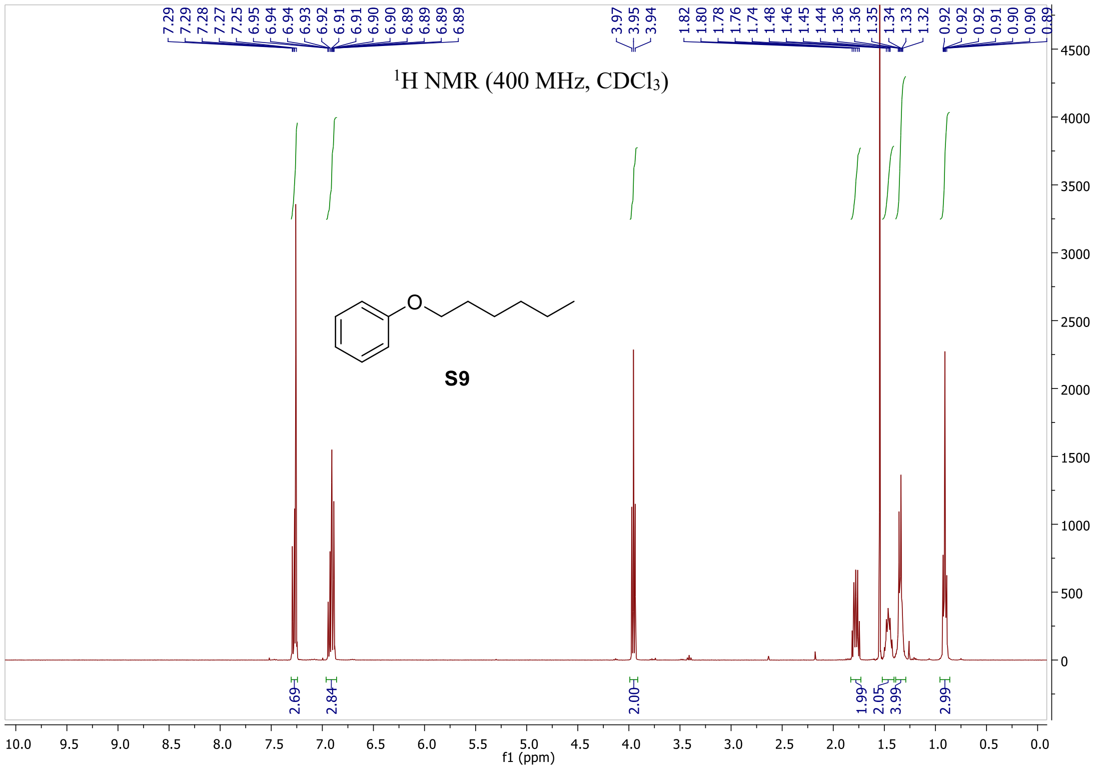

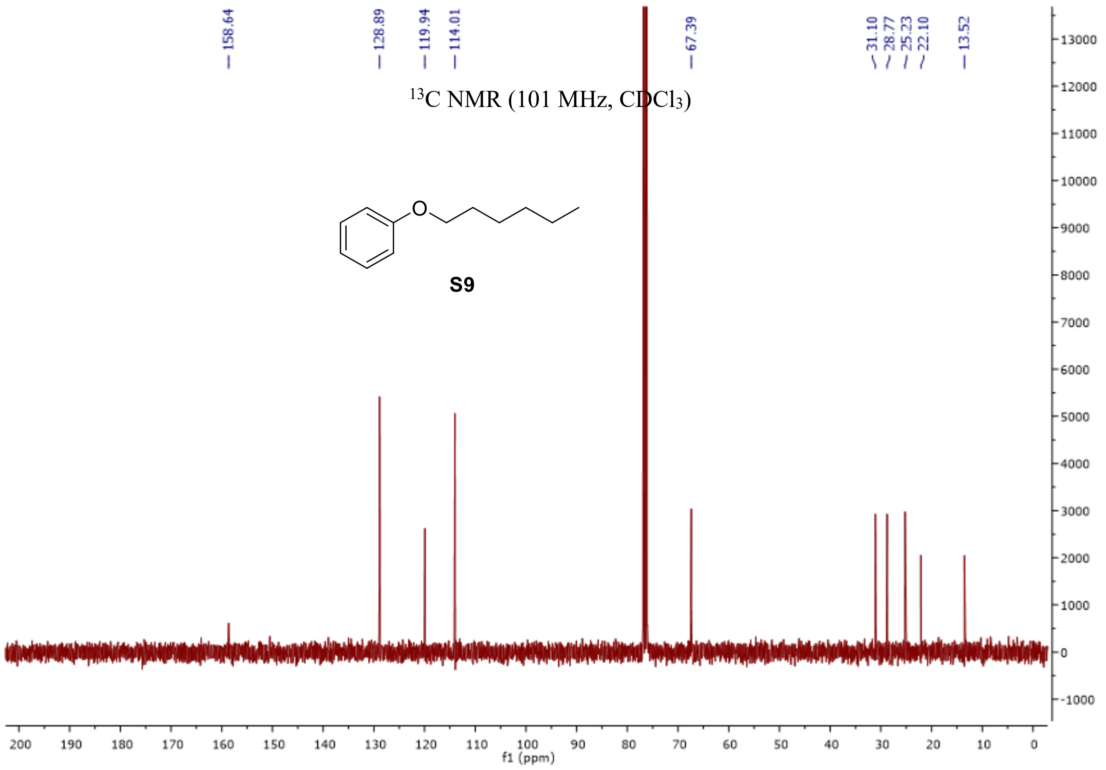

3-Phenoxypropylbenzene (**S11**)

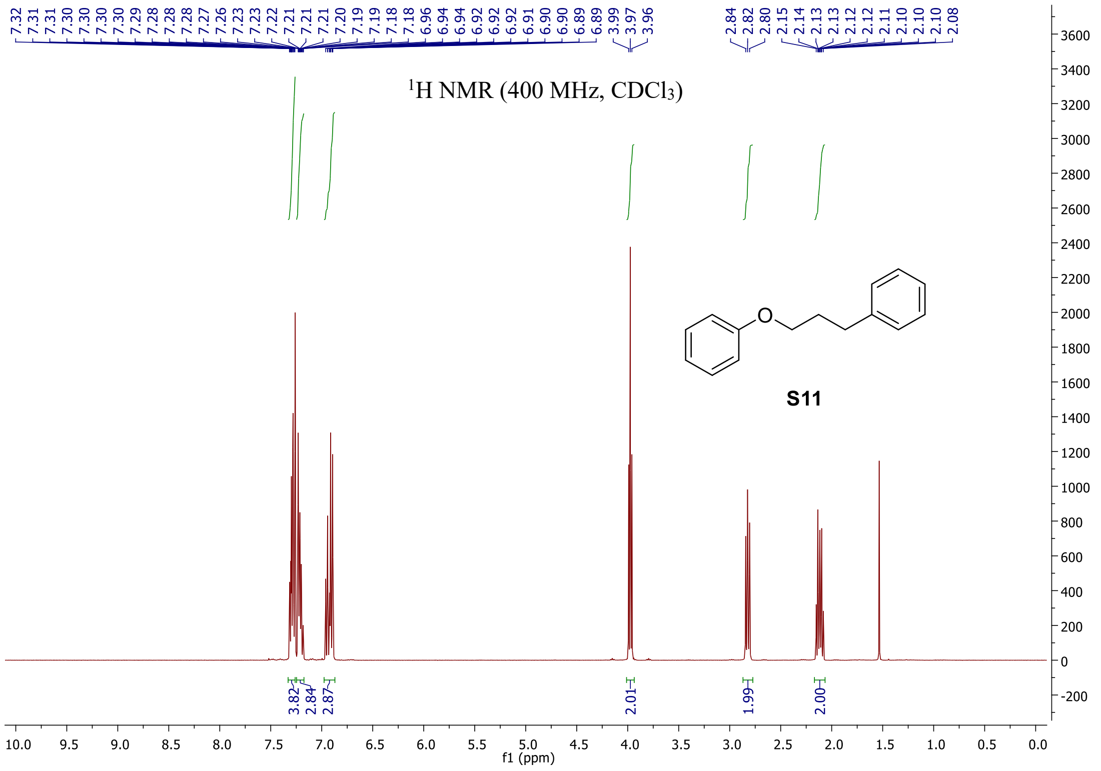

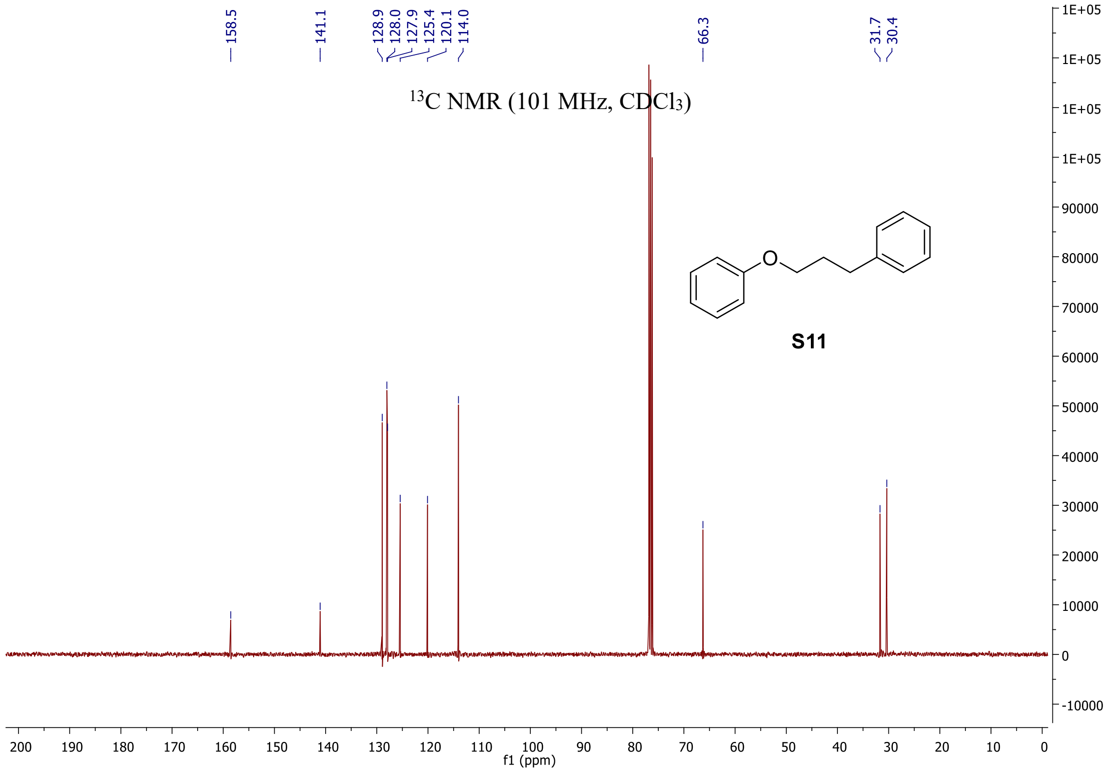

(Cyclopentyloxy)benzene (**S13**)

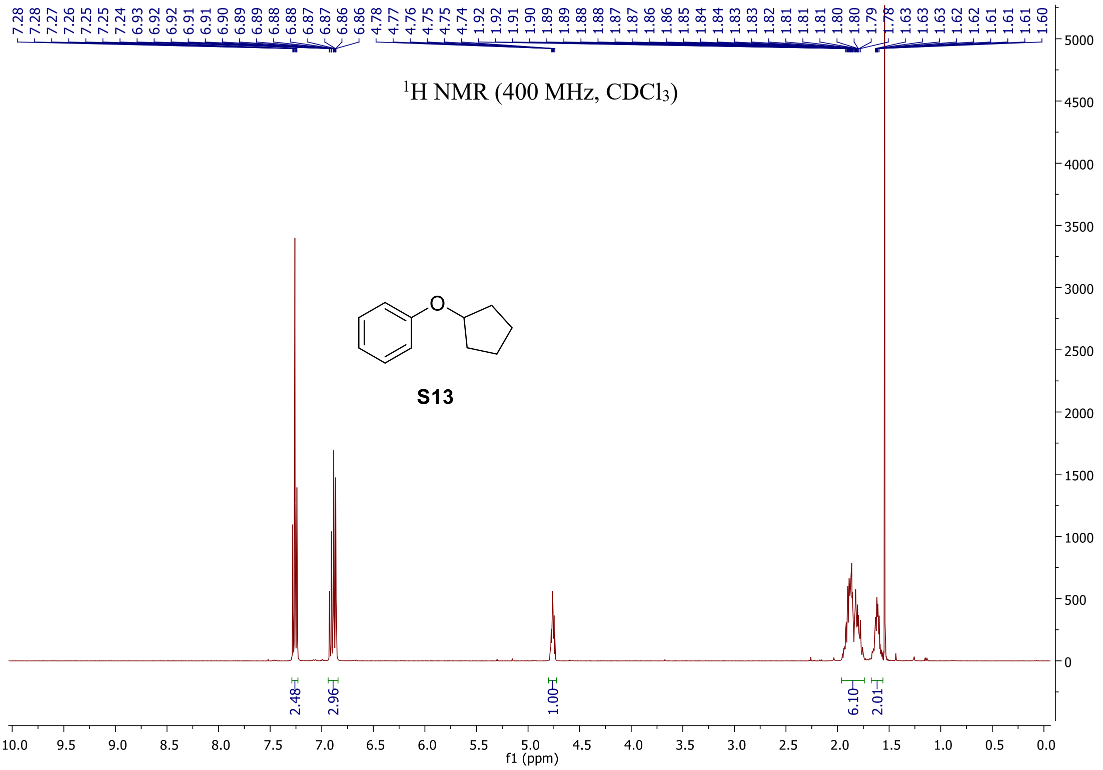

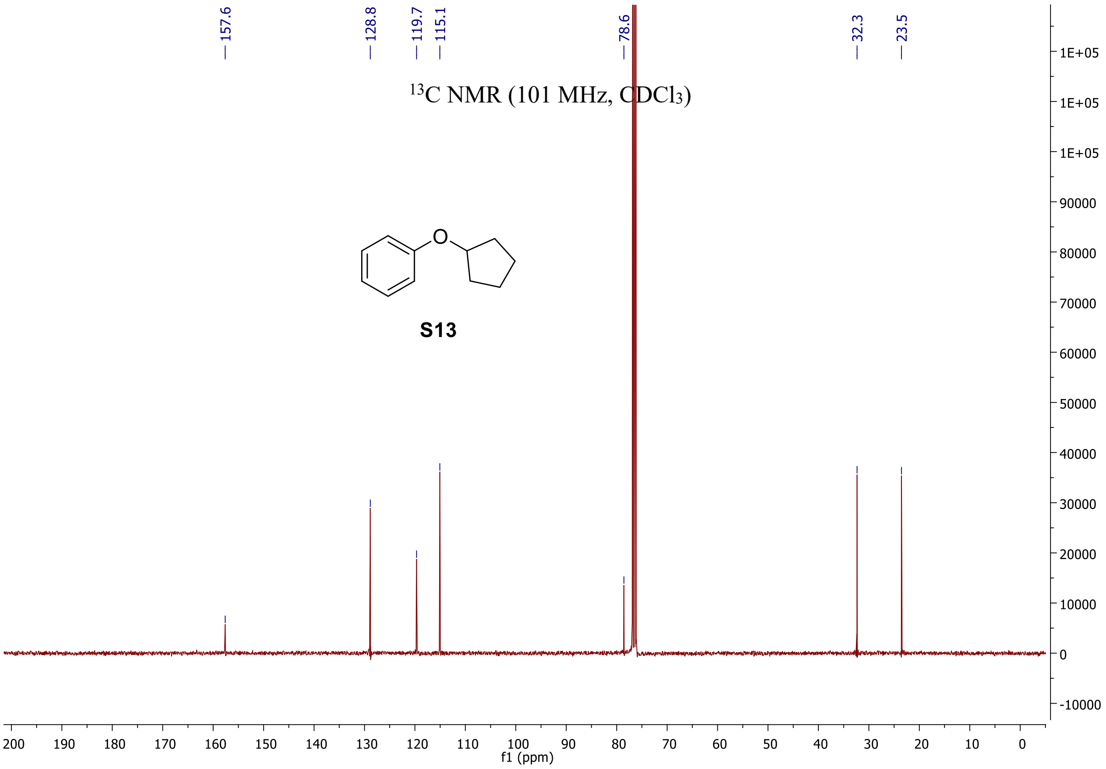

2-(3-Phenoxypropyl)isoindoline-1,3-dione (**S15**)

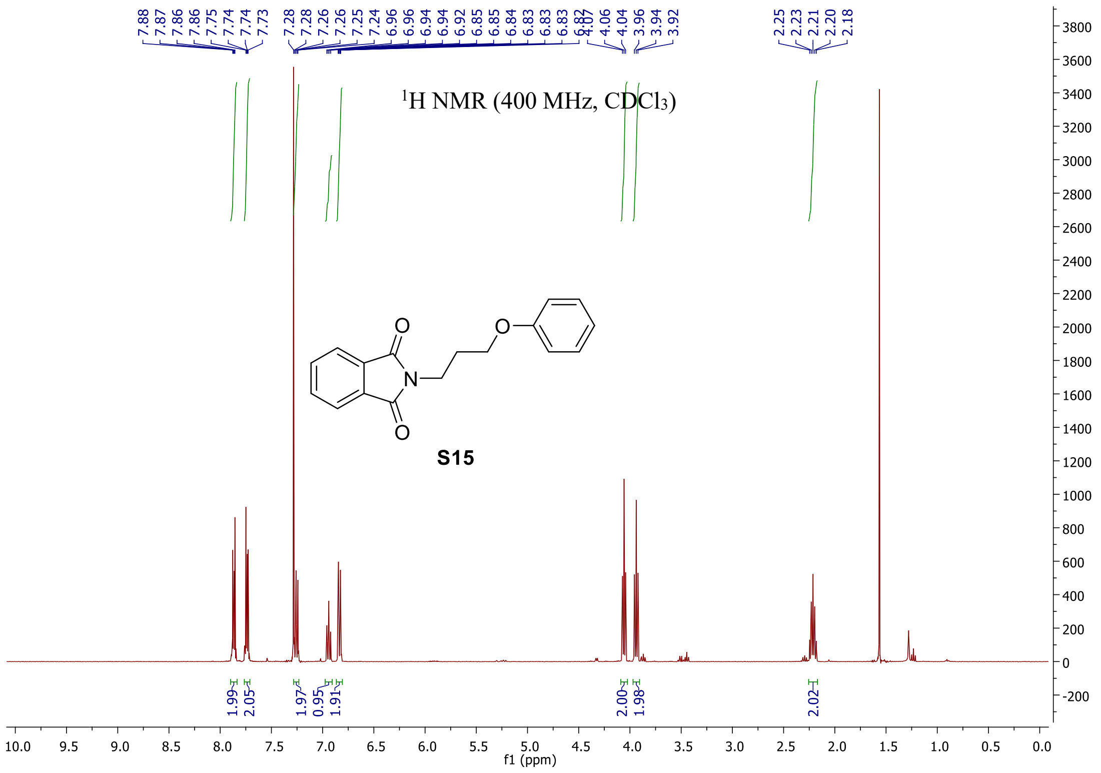

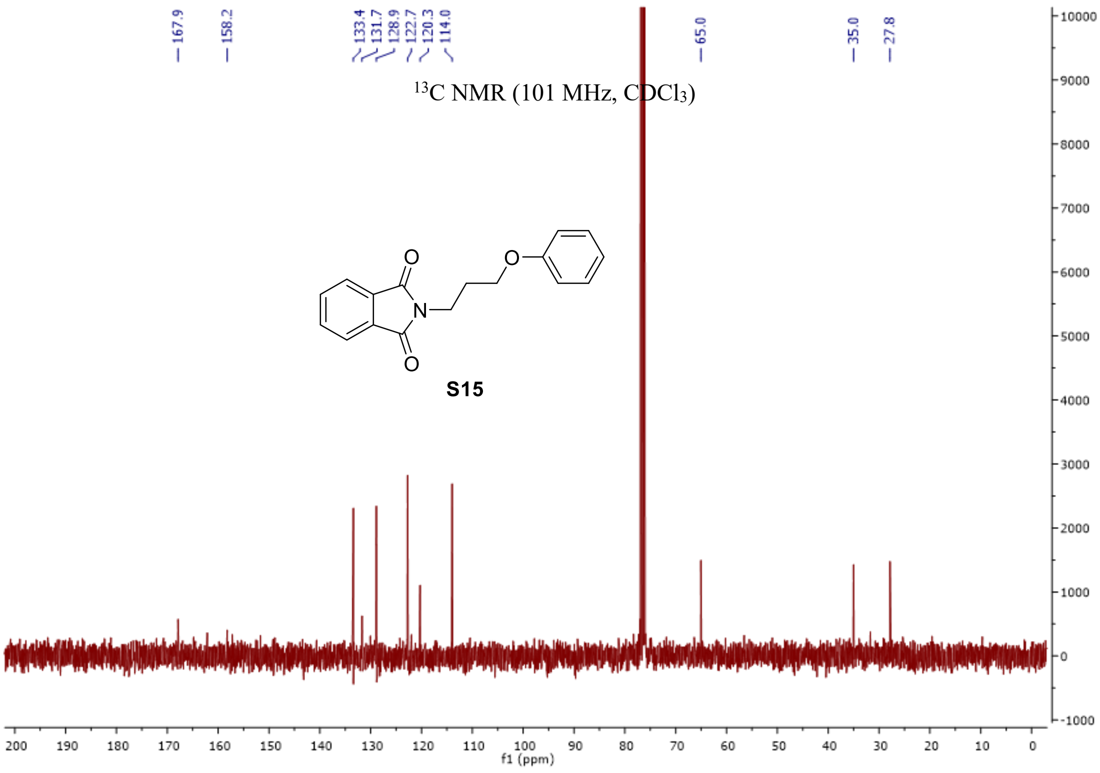

Allylic Sulfone Substrates

(Prop-2-ene-1,2-diyldisulfonyl)dibenzene (**S17**)

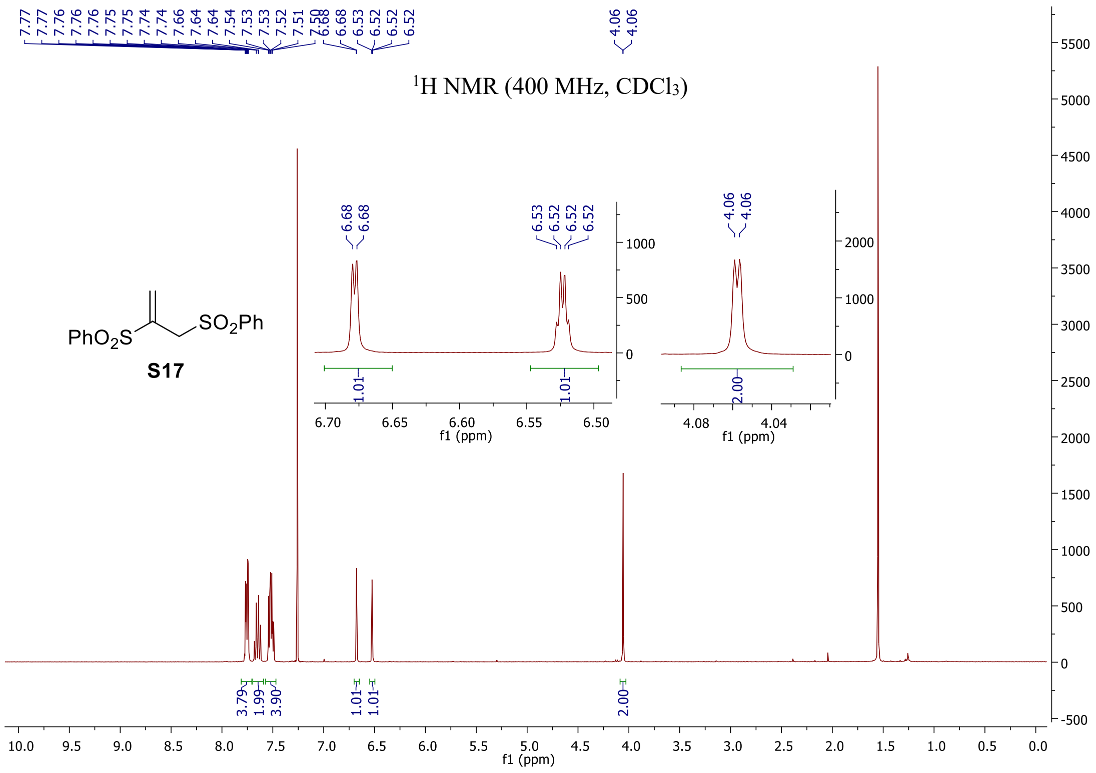

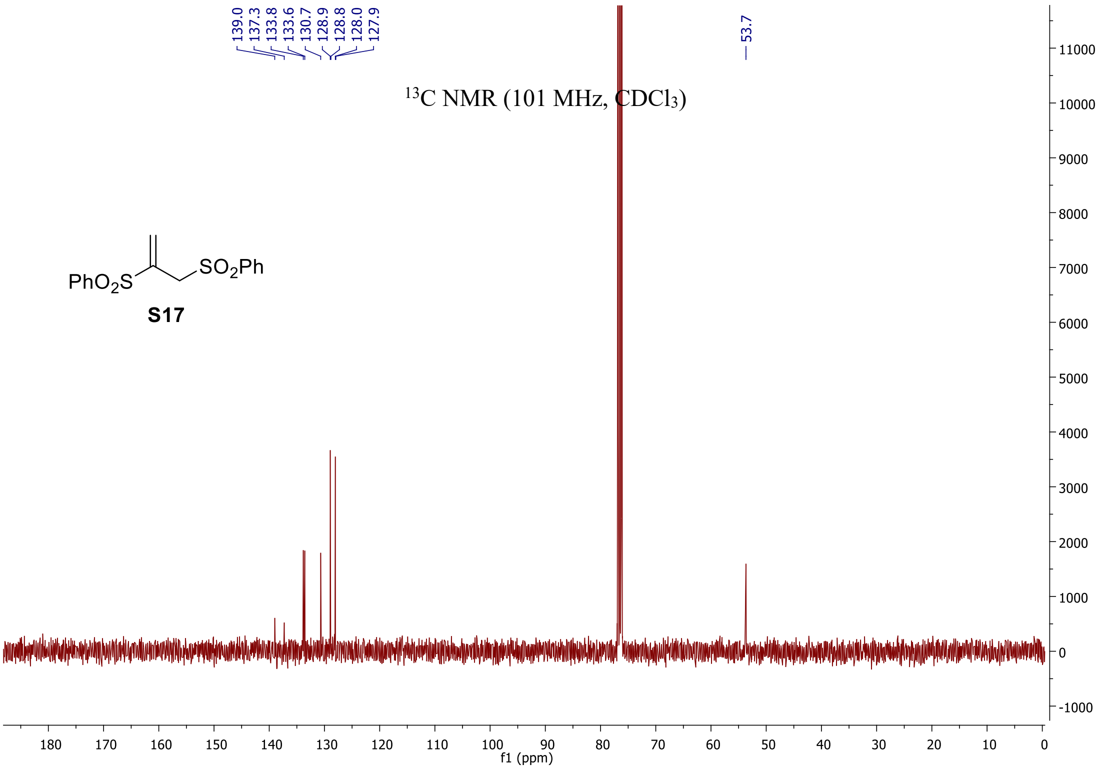

Ethyl 2-((phenylsulfonyl)methyl)acrylate (**S19**)

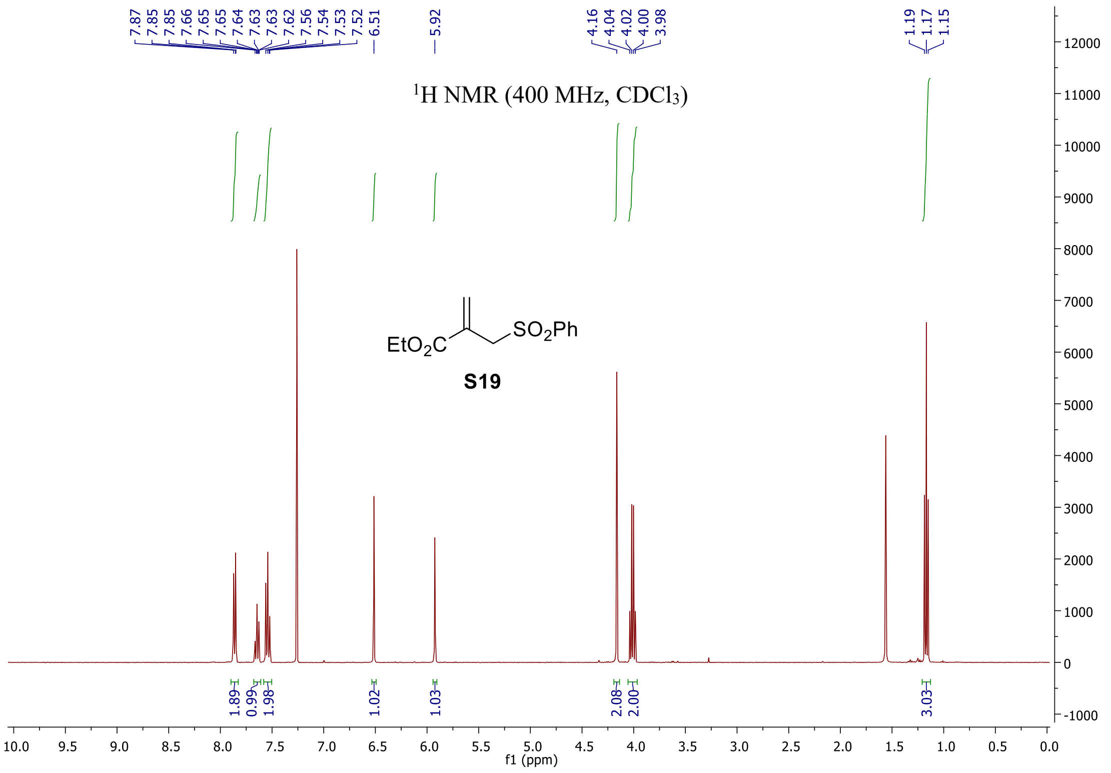

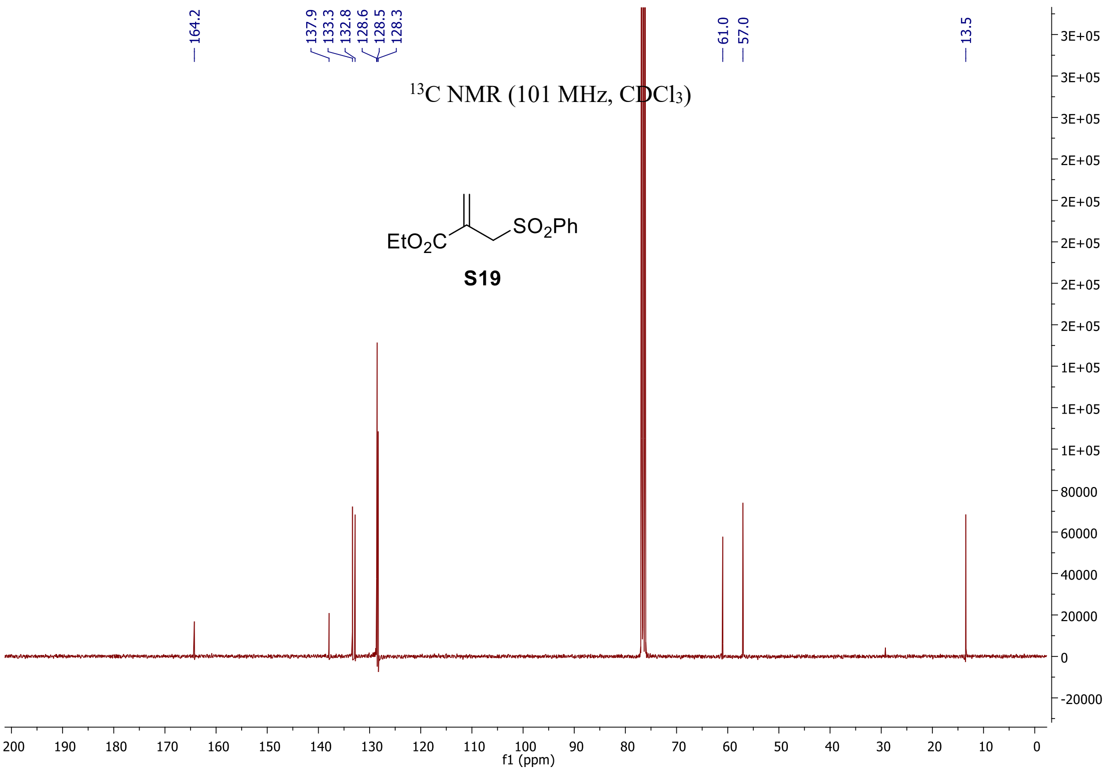

1-Phenyl-2-((phenylsulfonyl)methyl)prop-2-en-1-one (**S21**)

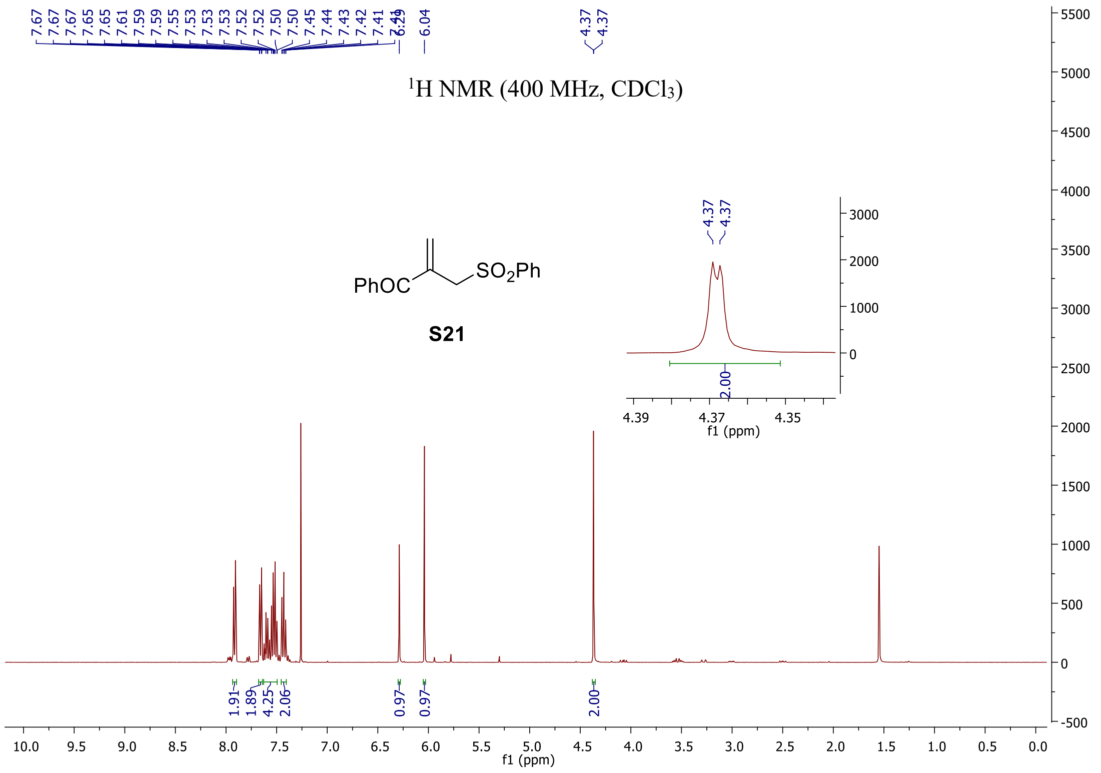

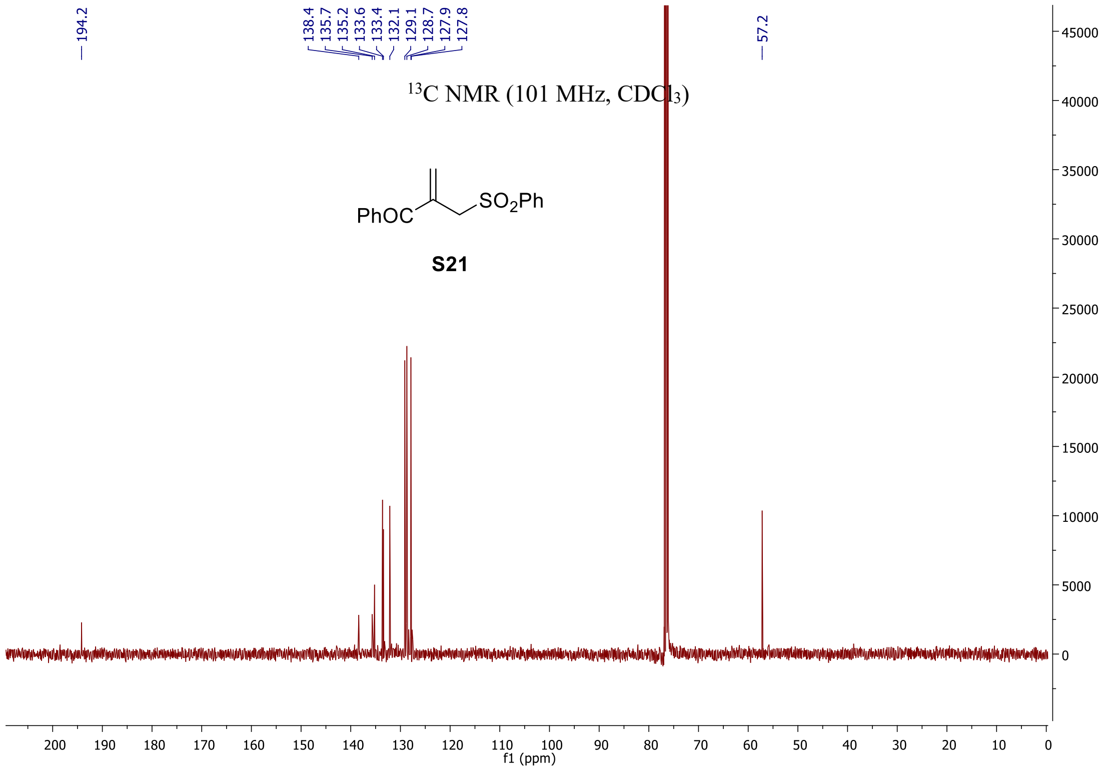

((2-Bromoallyl)sulfonyl)benzene (**S22**)

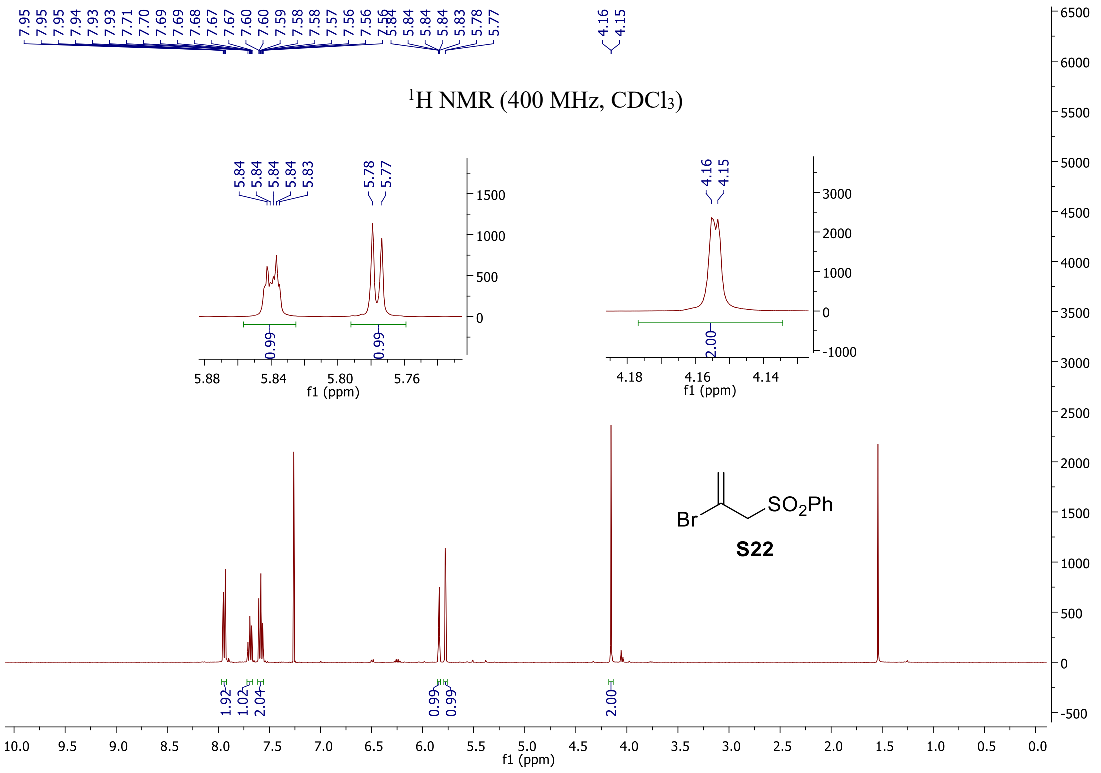

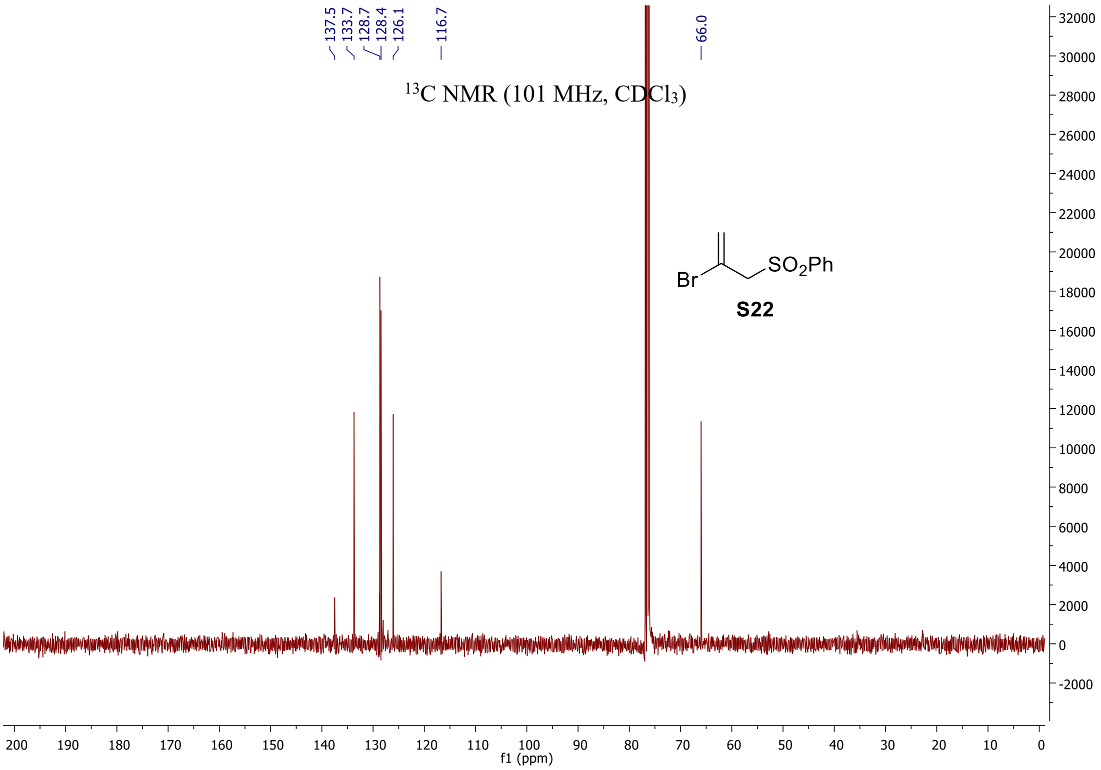

(3-Bromoprop-1-en-2-yl)benzene (**S24**)

Crude ^{1}H NMR Spectrum

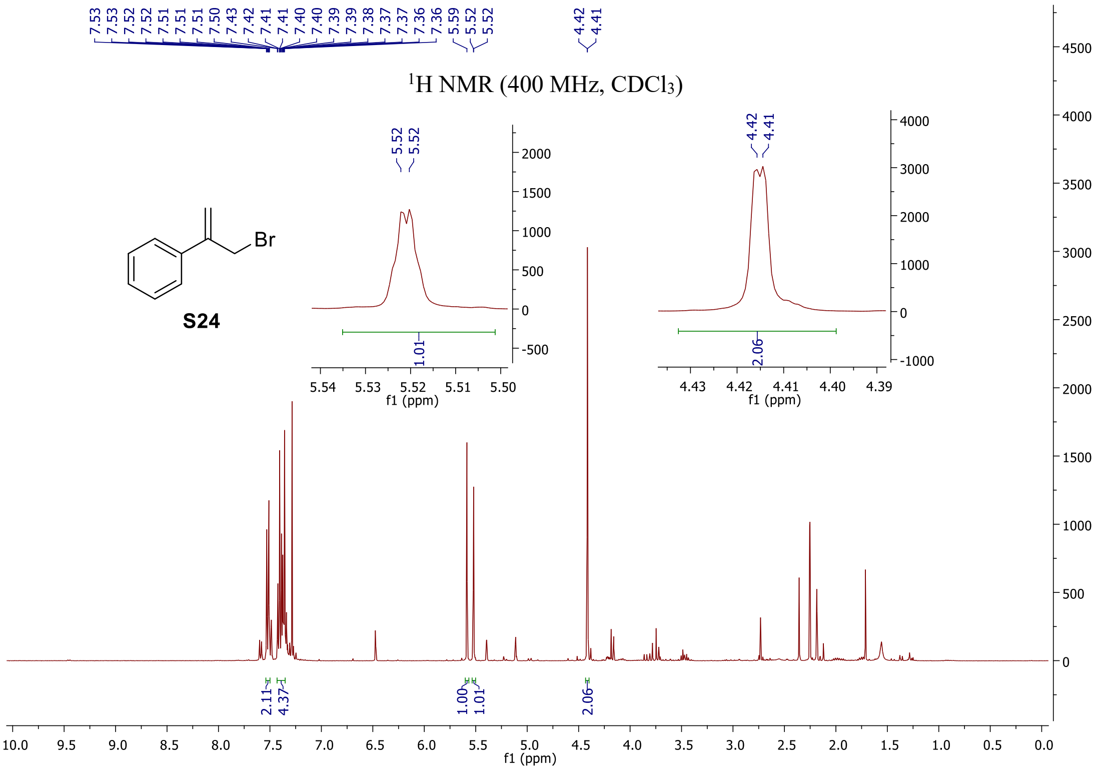

((2-Phenylallyl)sulfonyl)benzene (**S25**)

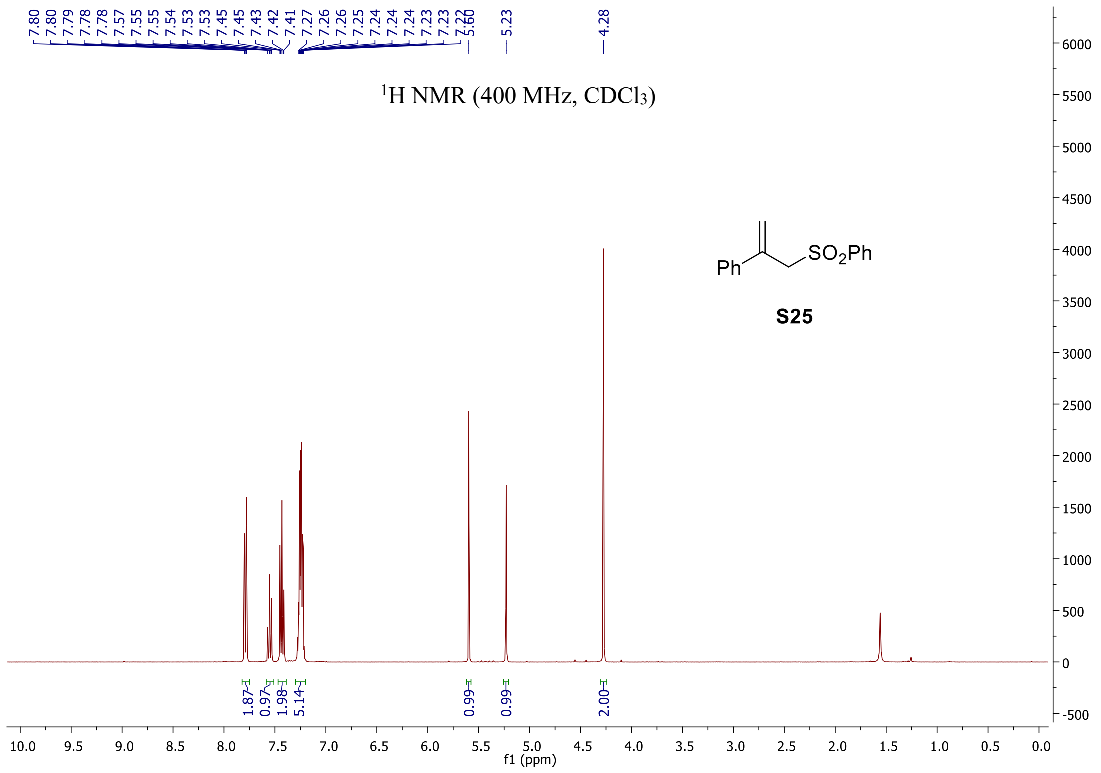

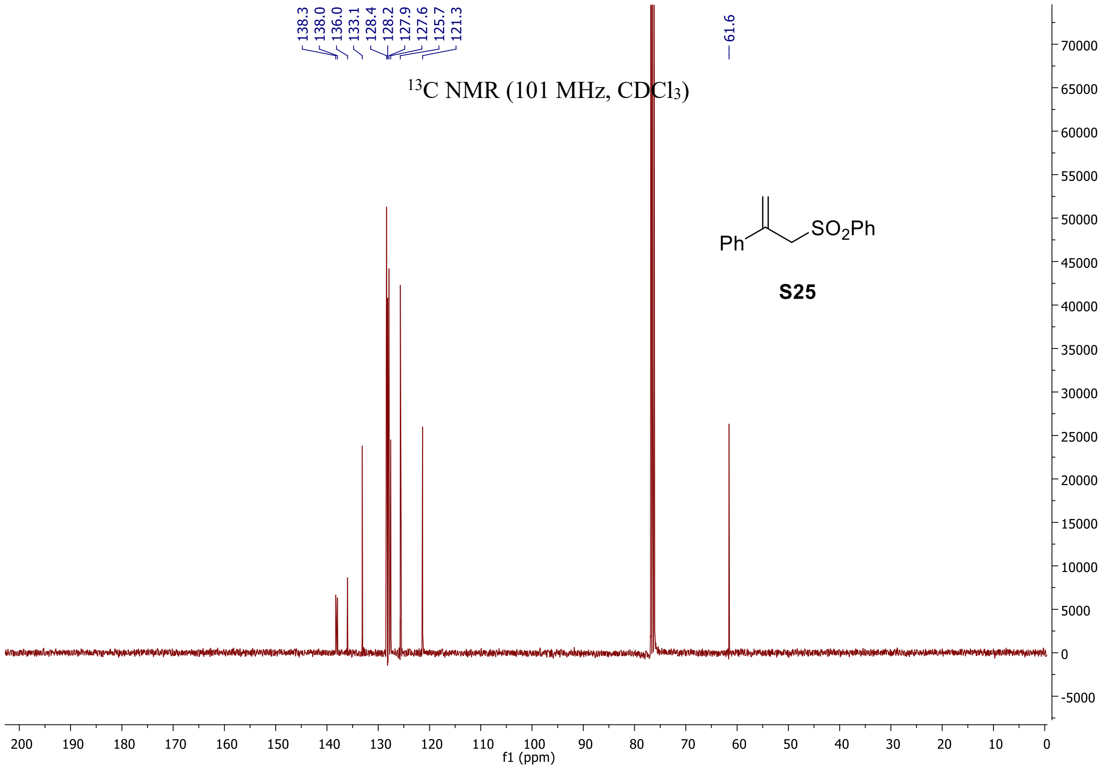

Cyanation Products

2-Phenoxyacetonitrile (**15**)

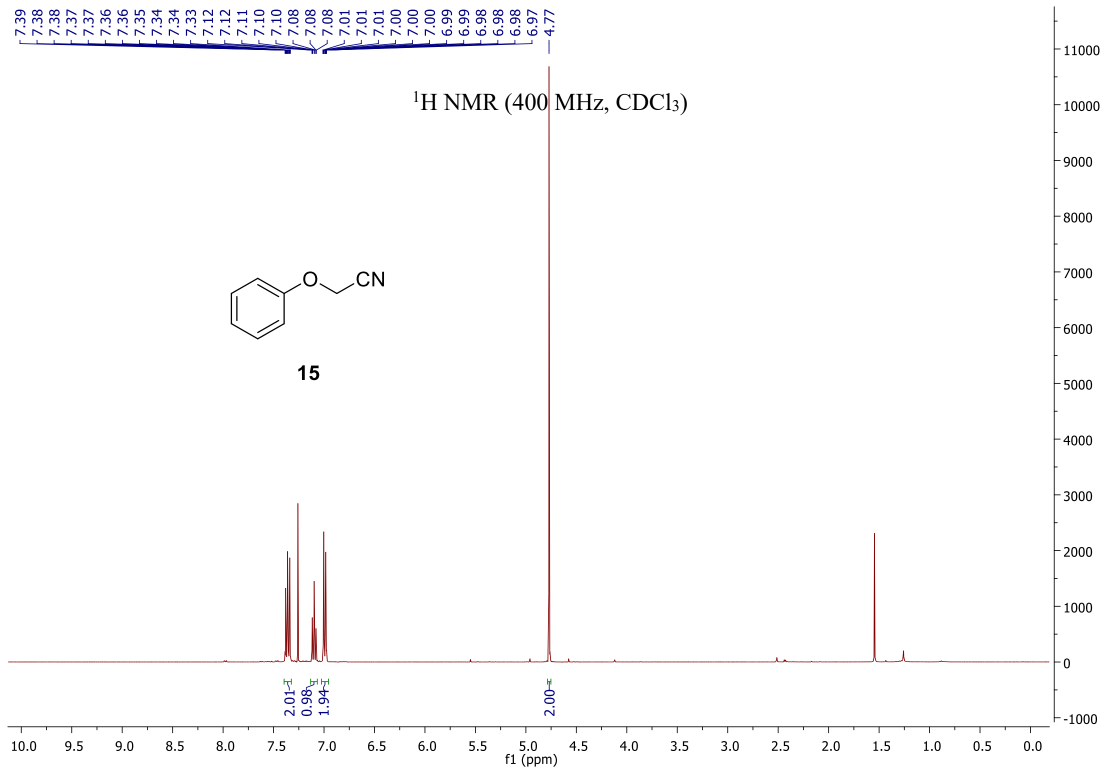

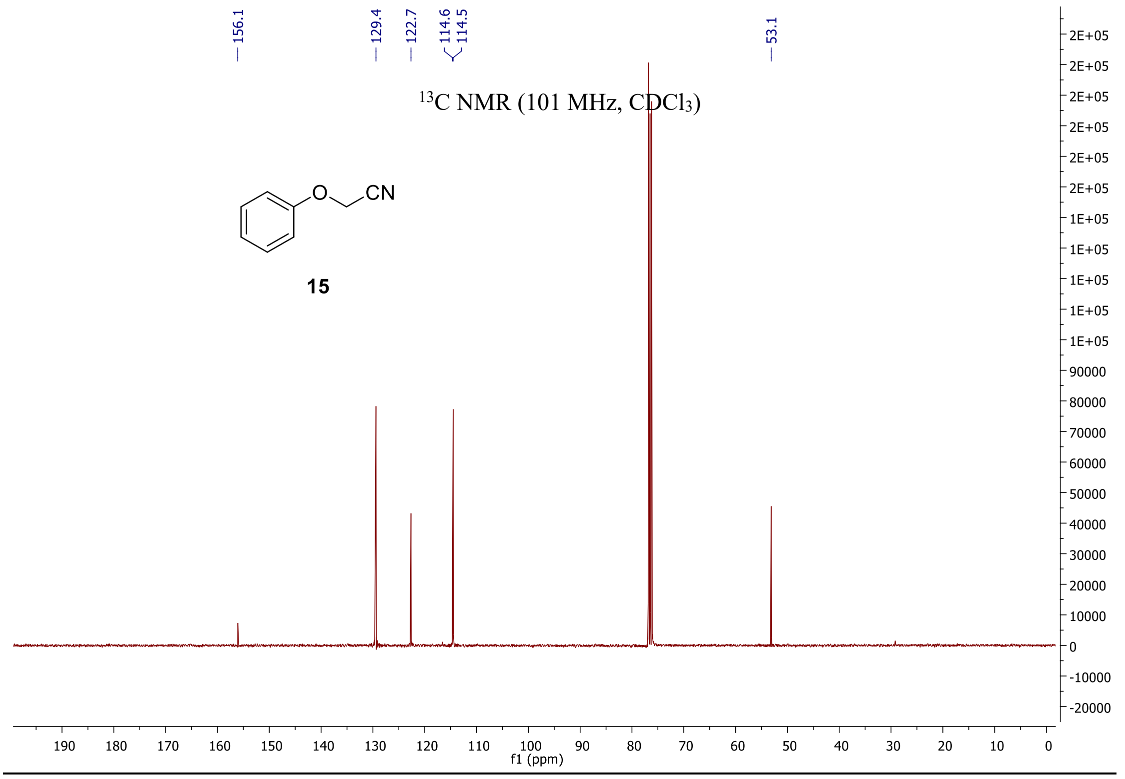

2-Methyl-2-phenoxypropanenitrile (**17**)

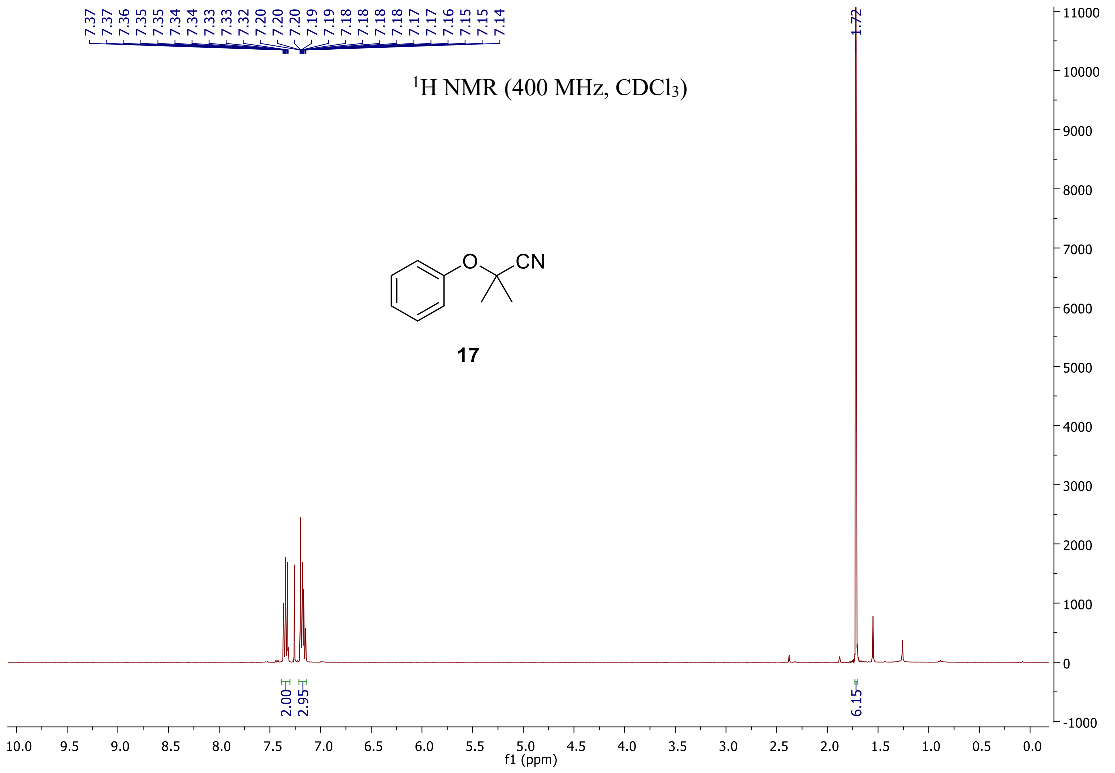

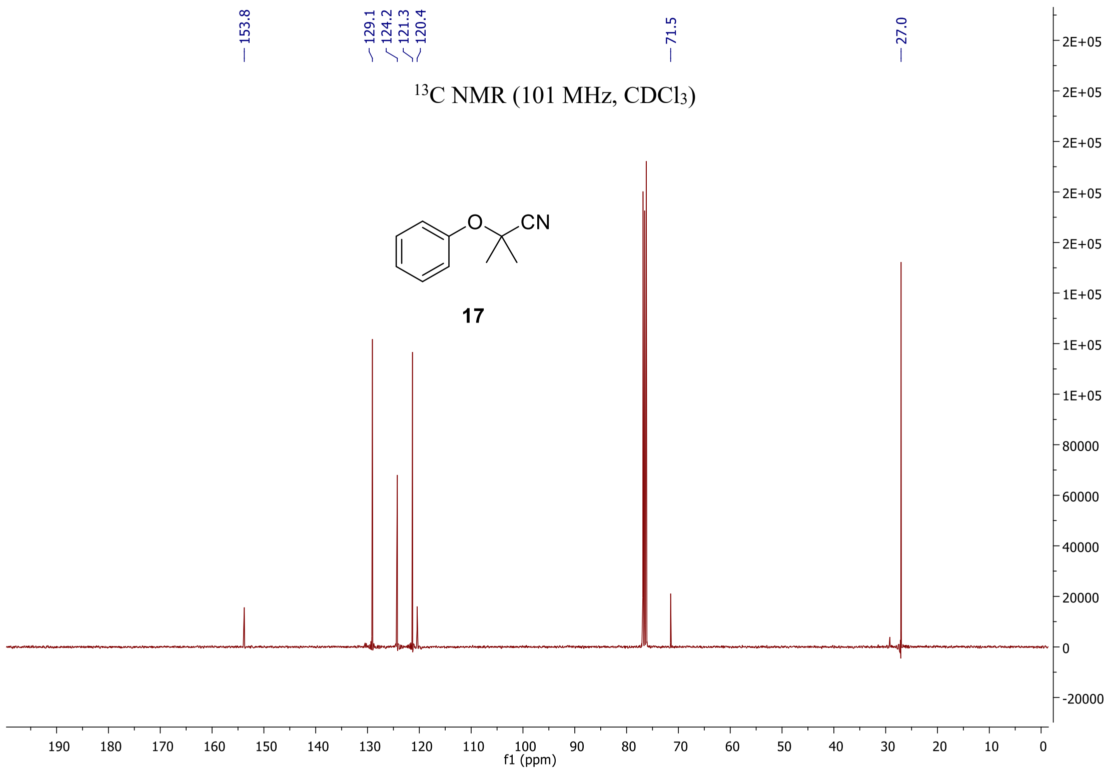

2-Phenoxyheptanenitrile (**18**)

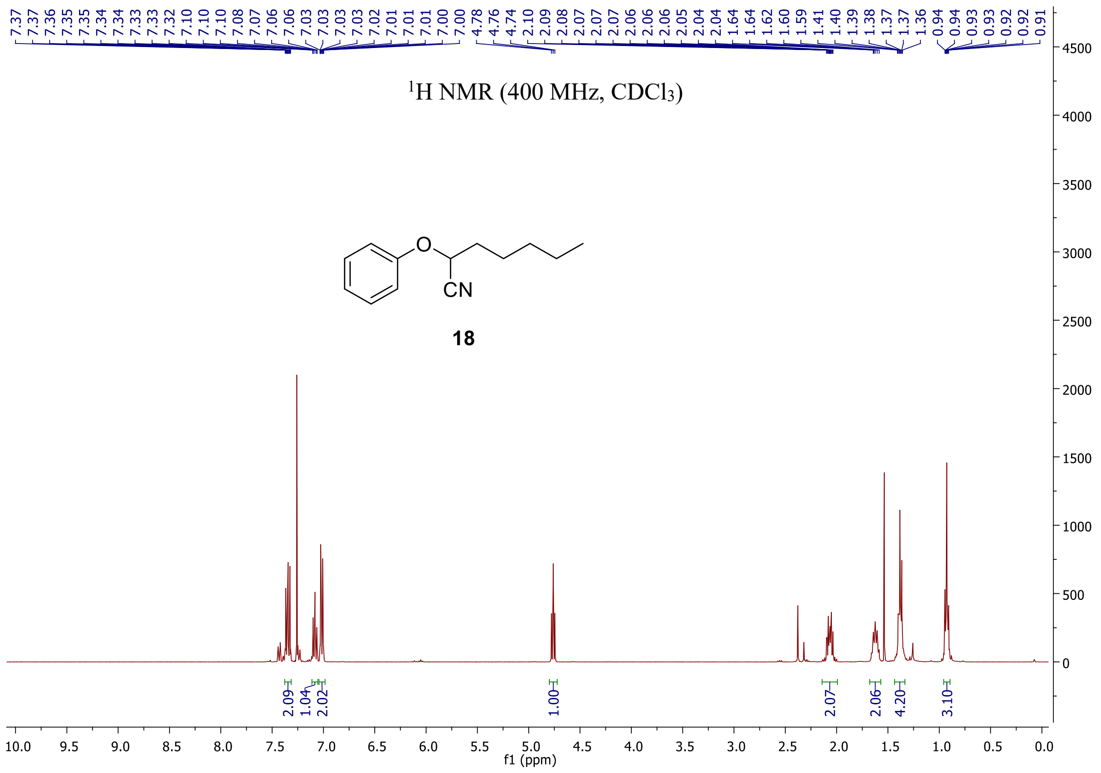

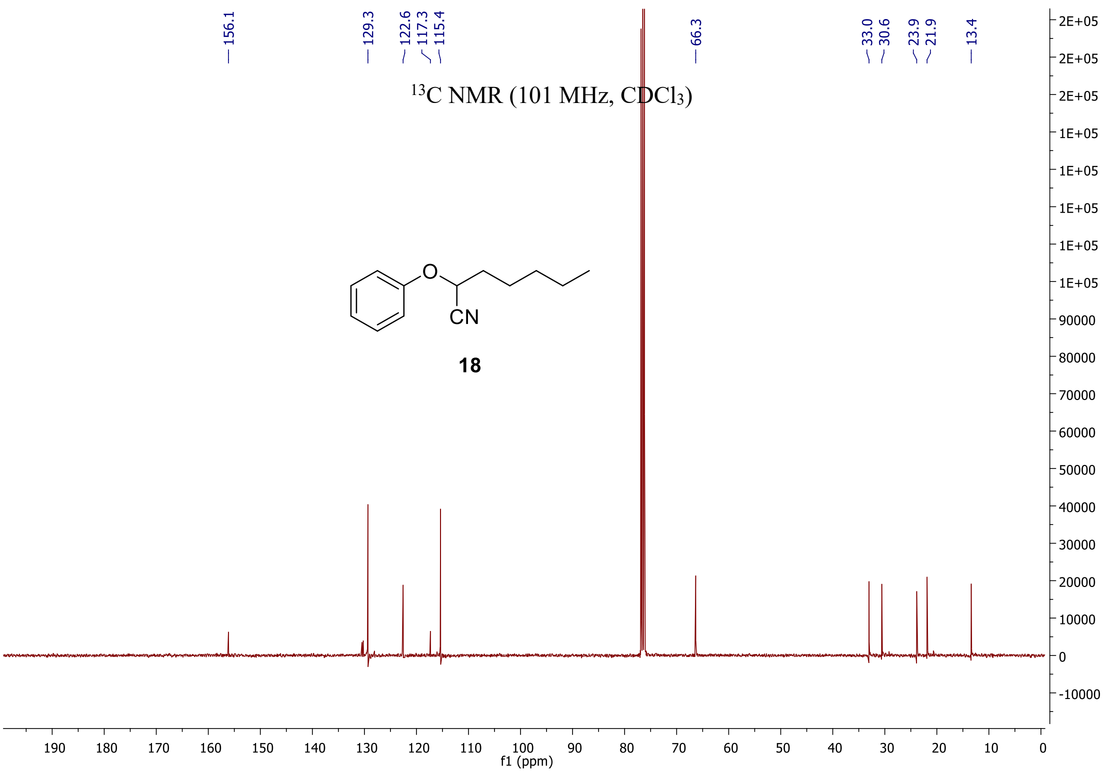

2-Phenoxy-4-phenylbutanenitrile (**19**)

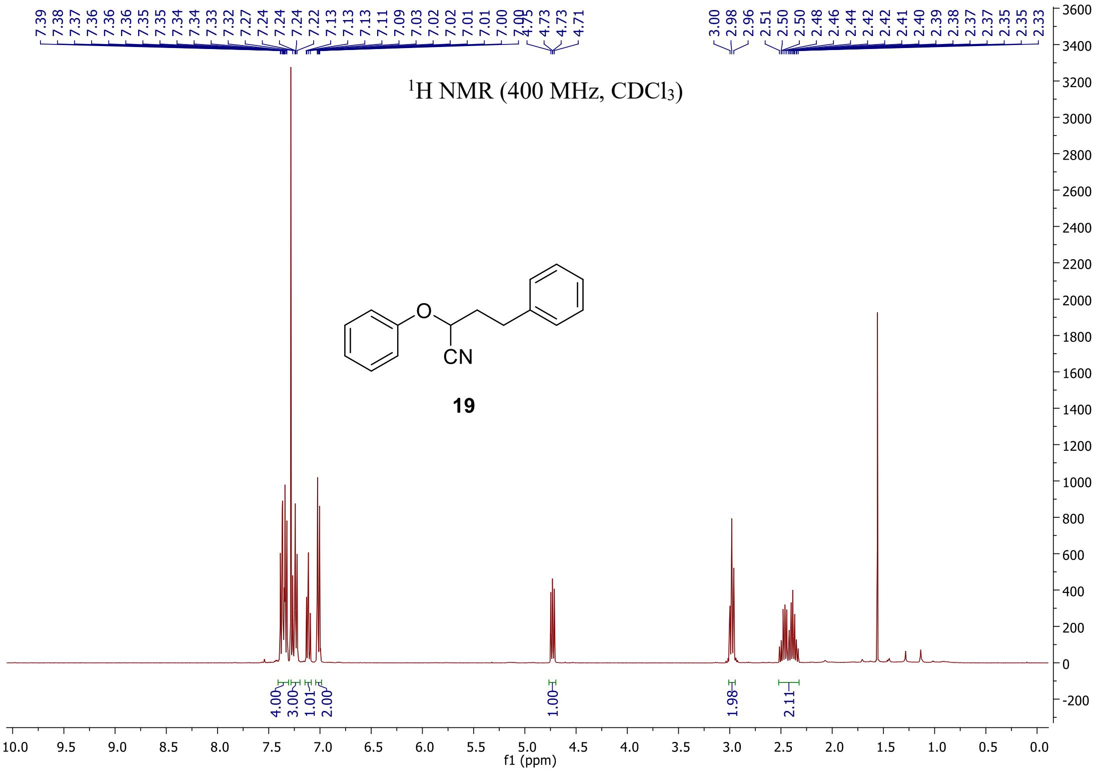

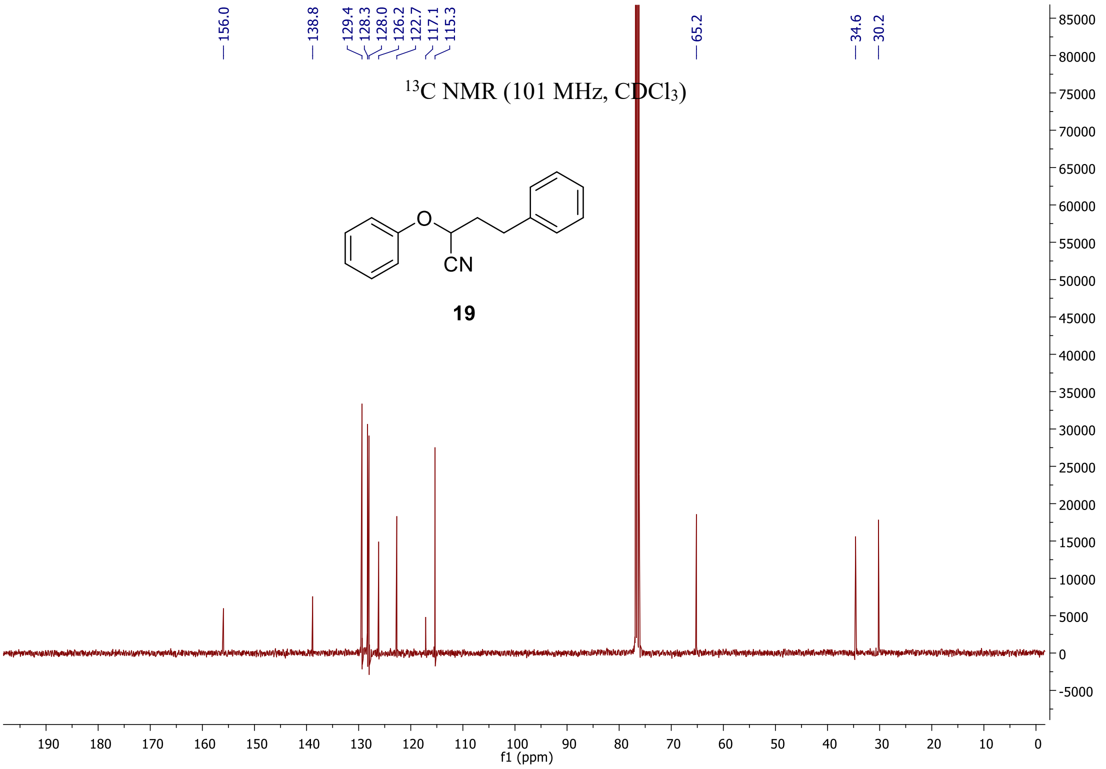

2-(*m*-Tolyloxy)acetonitrile (**20**)

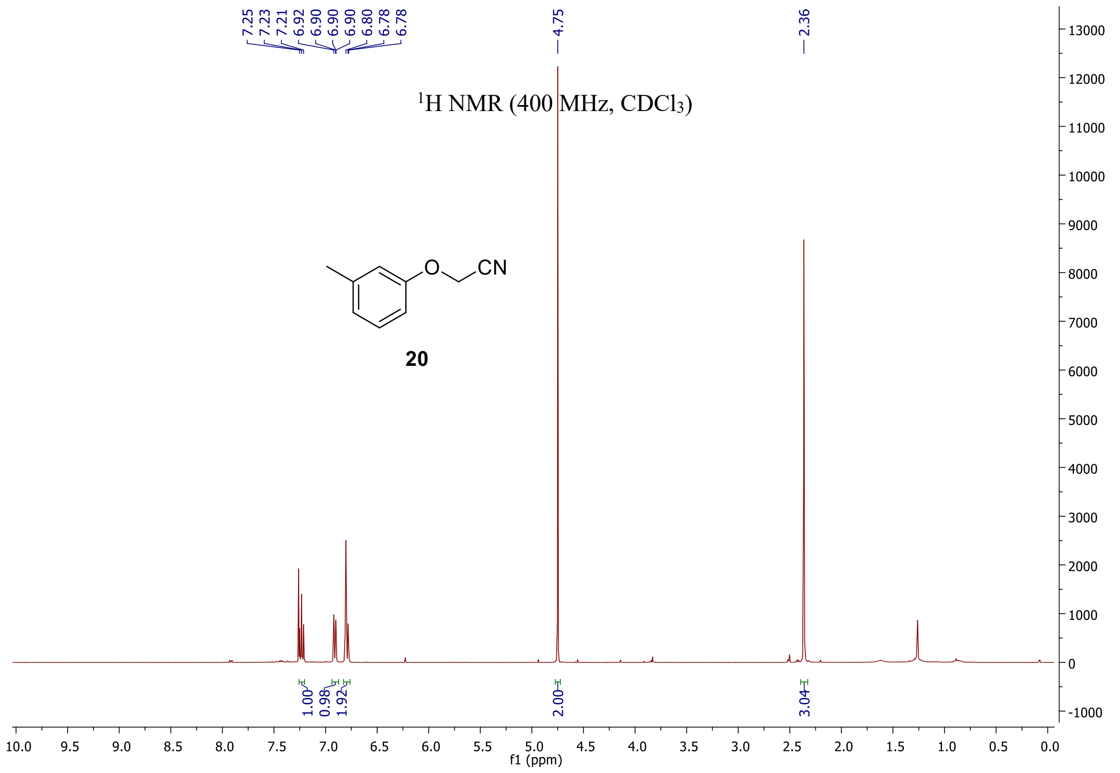

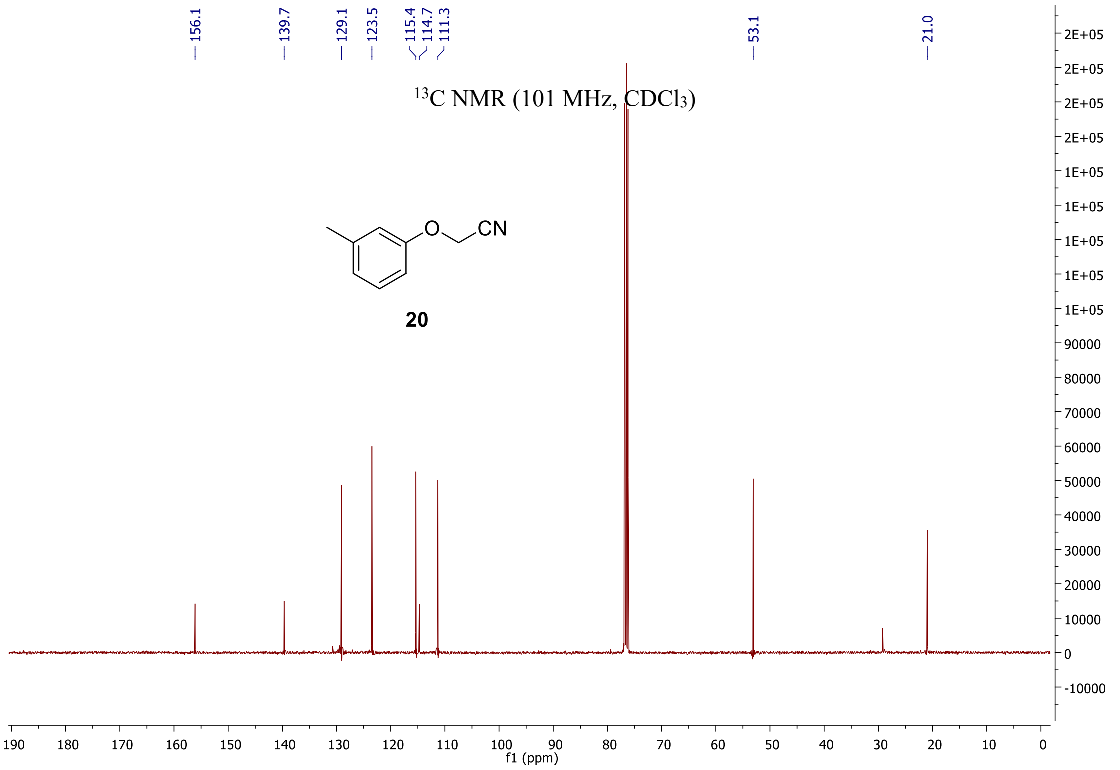

2-(3-Methoxyphenoxy)acetonitrile (**21**)

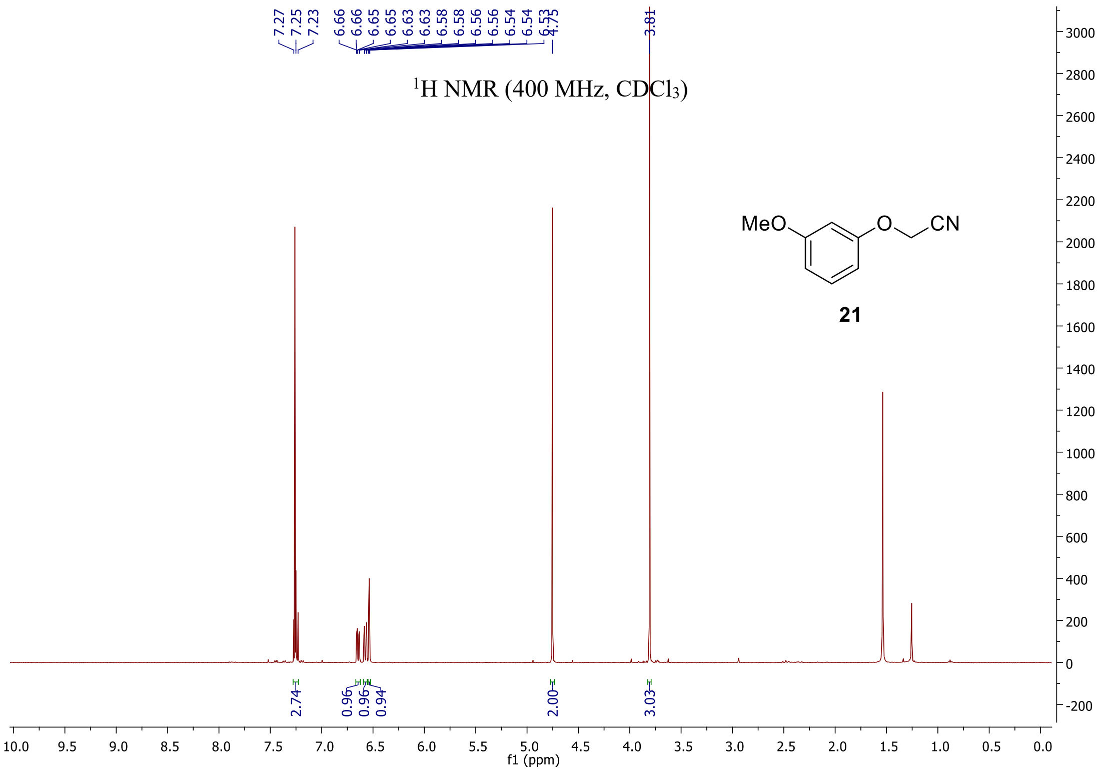

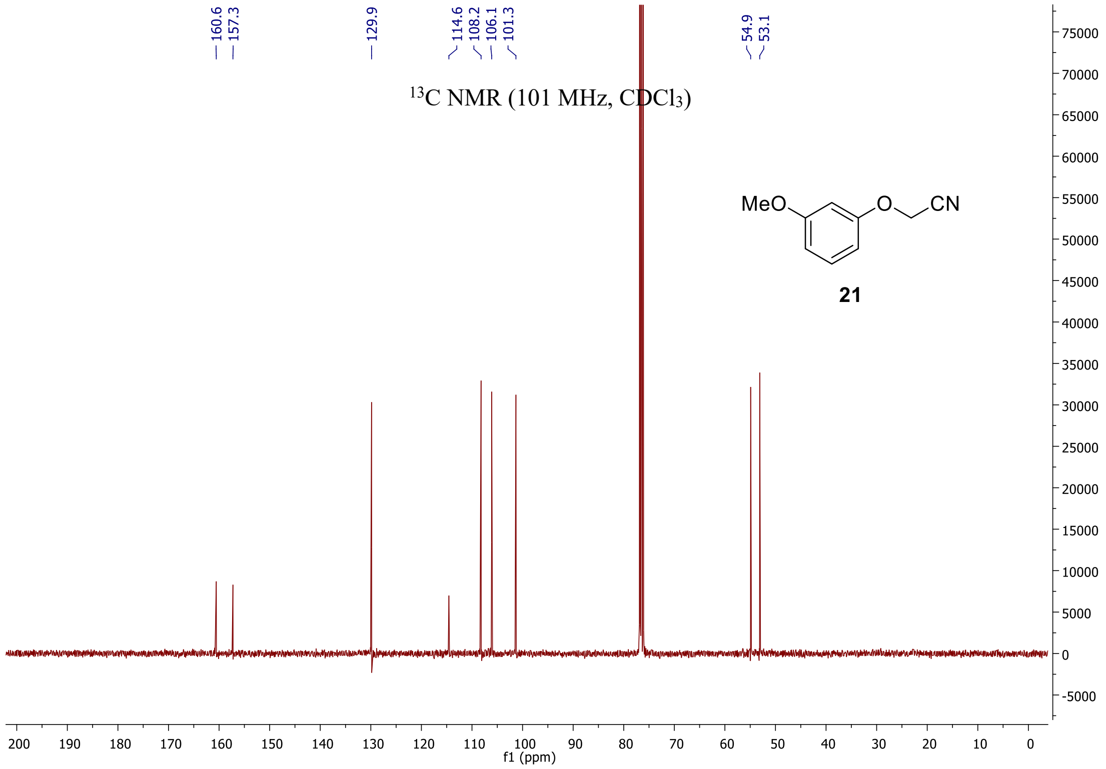

2-(4-Methoxyphenoxy)acetonitrile (**22**)

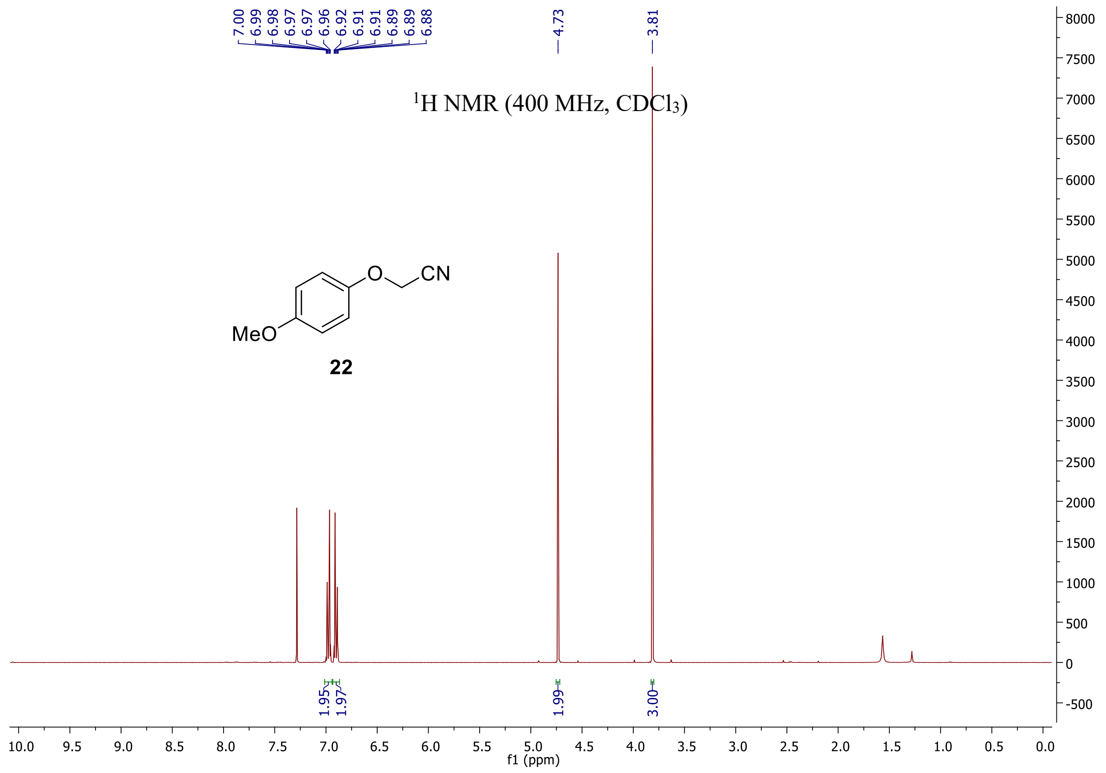

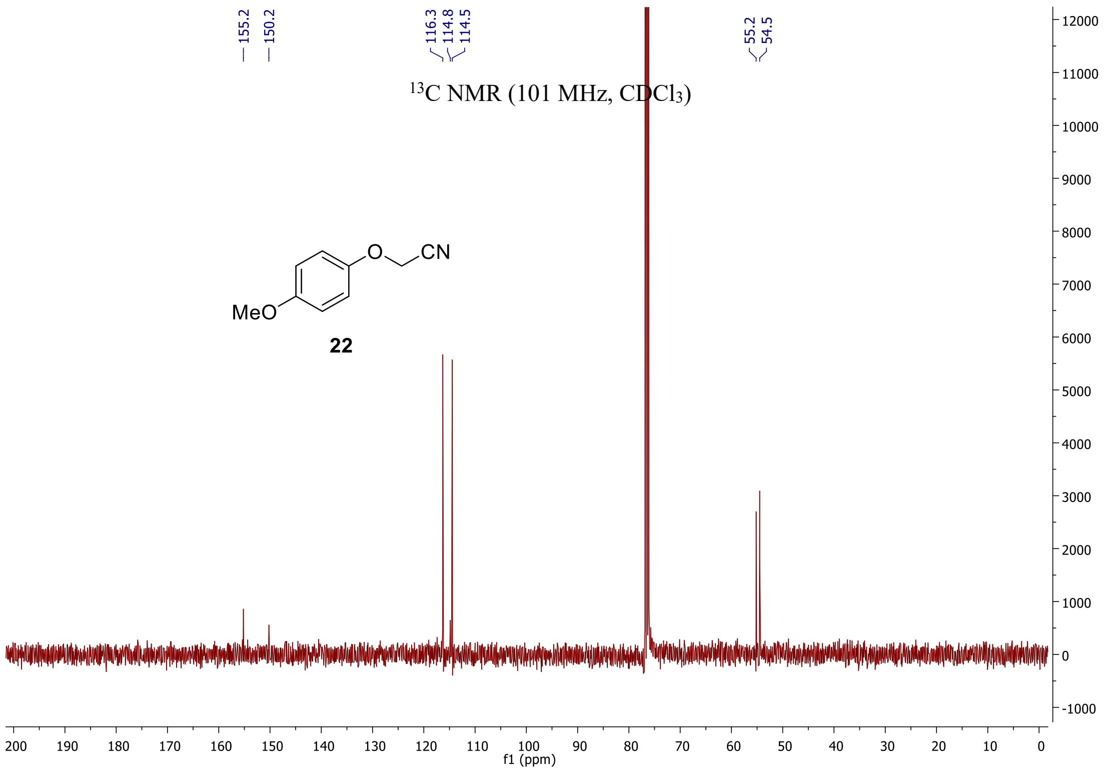

2-(4-Fluorophenoxy)acetonitrile (**23**)

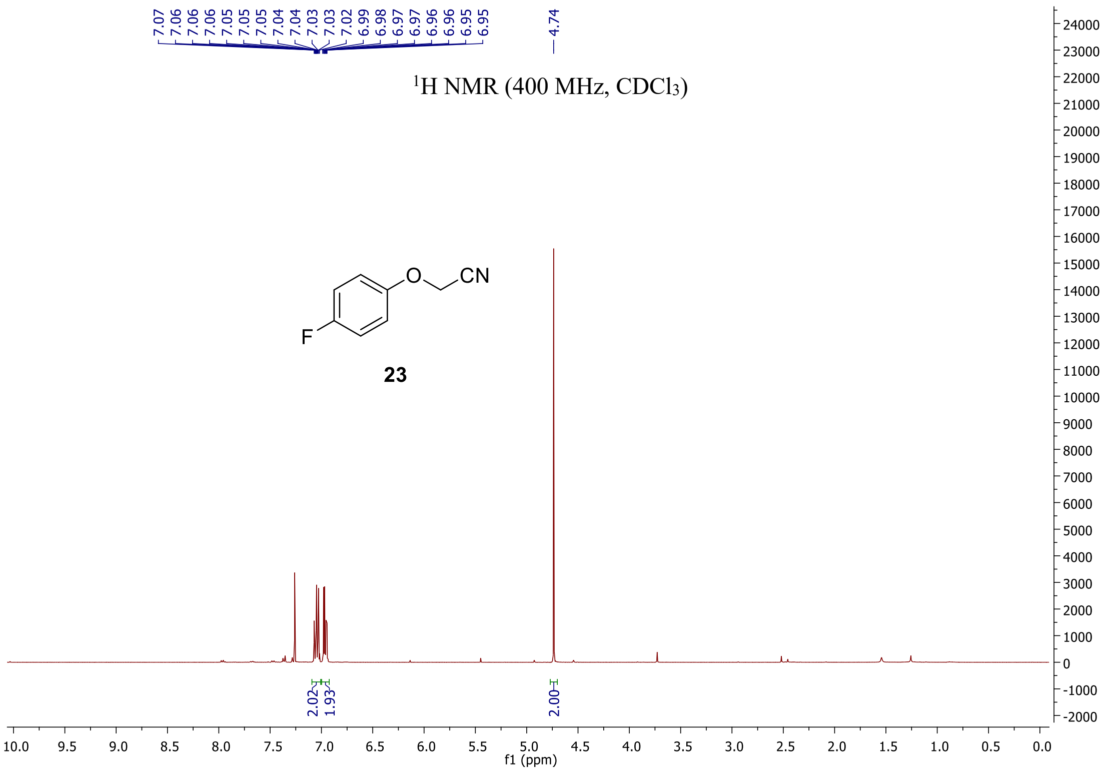

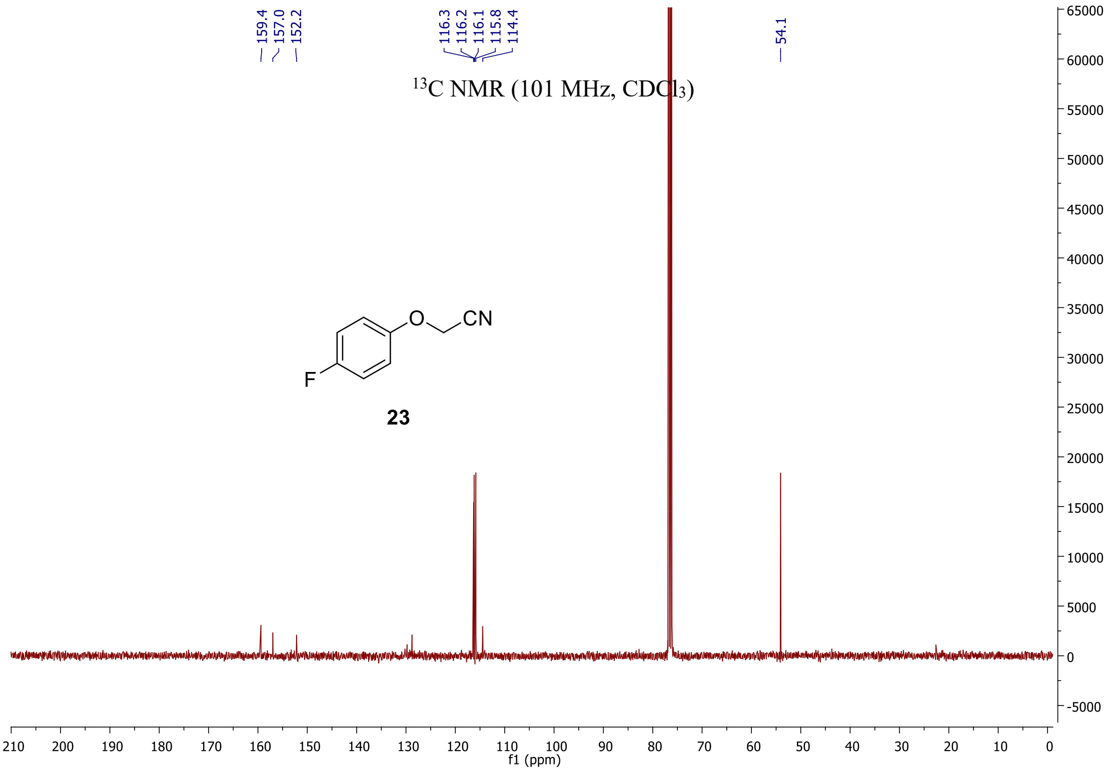

2-(4-Chlorophenoxy)acetonitrile (**24**)

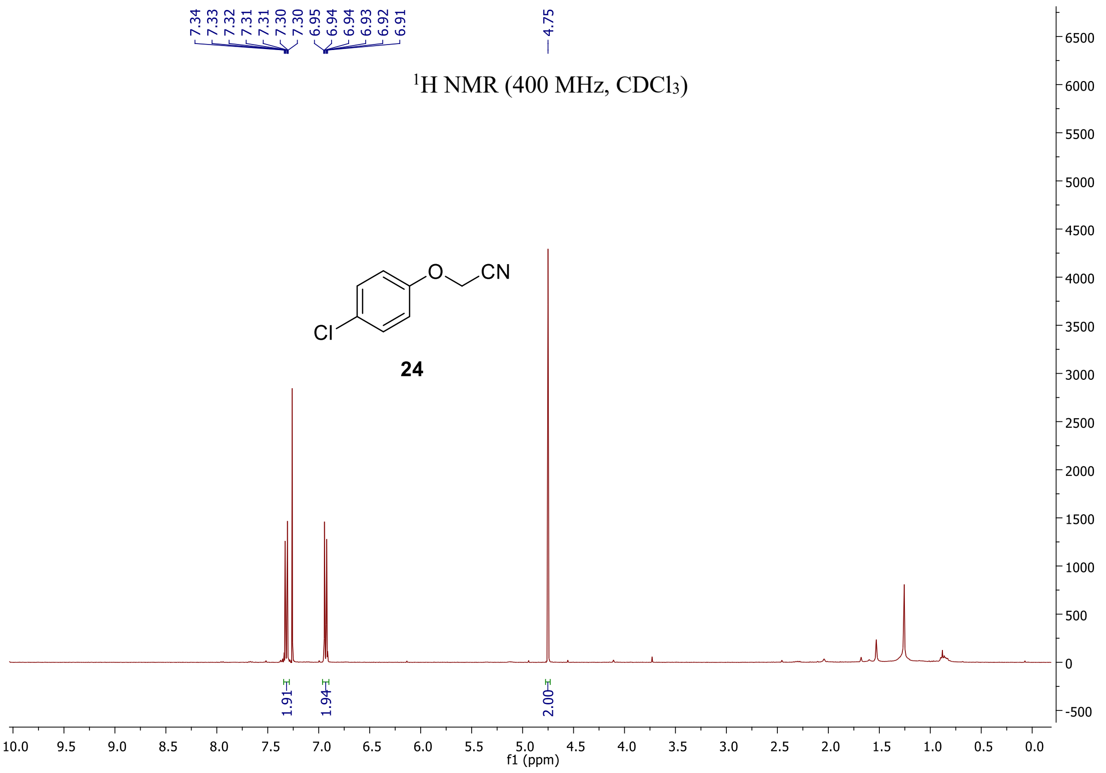

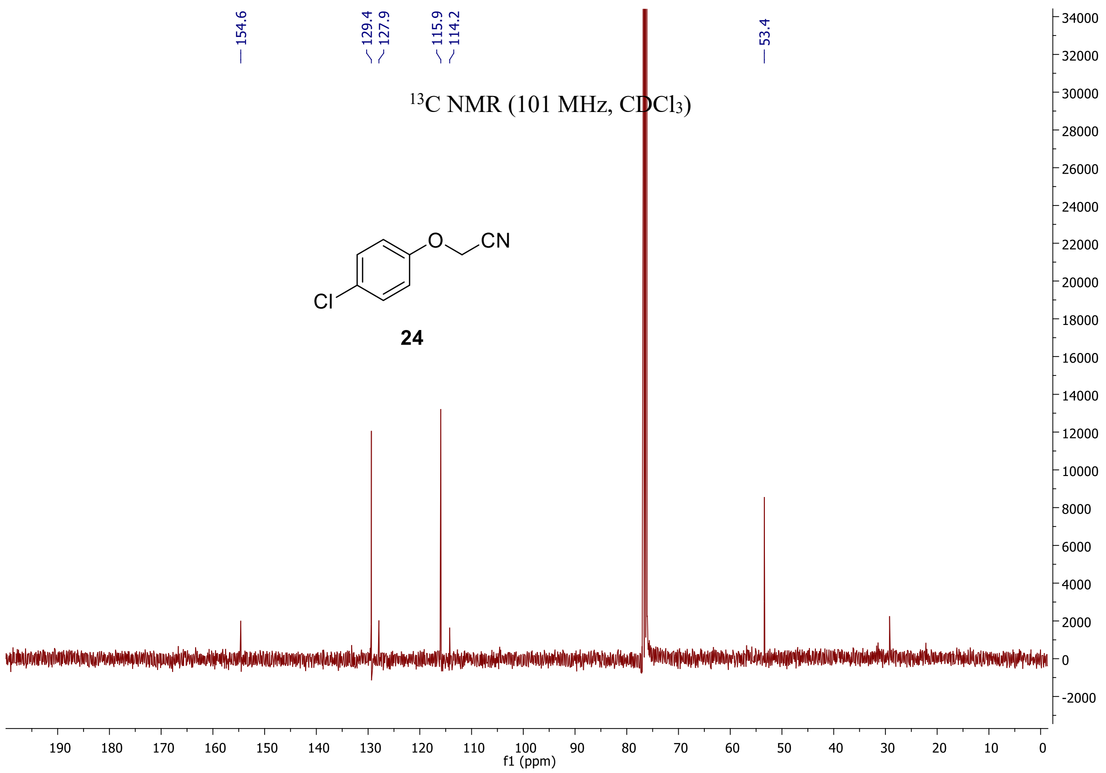

2-(4-Acetylphenoxy)acetonitrile (**25**)

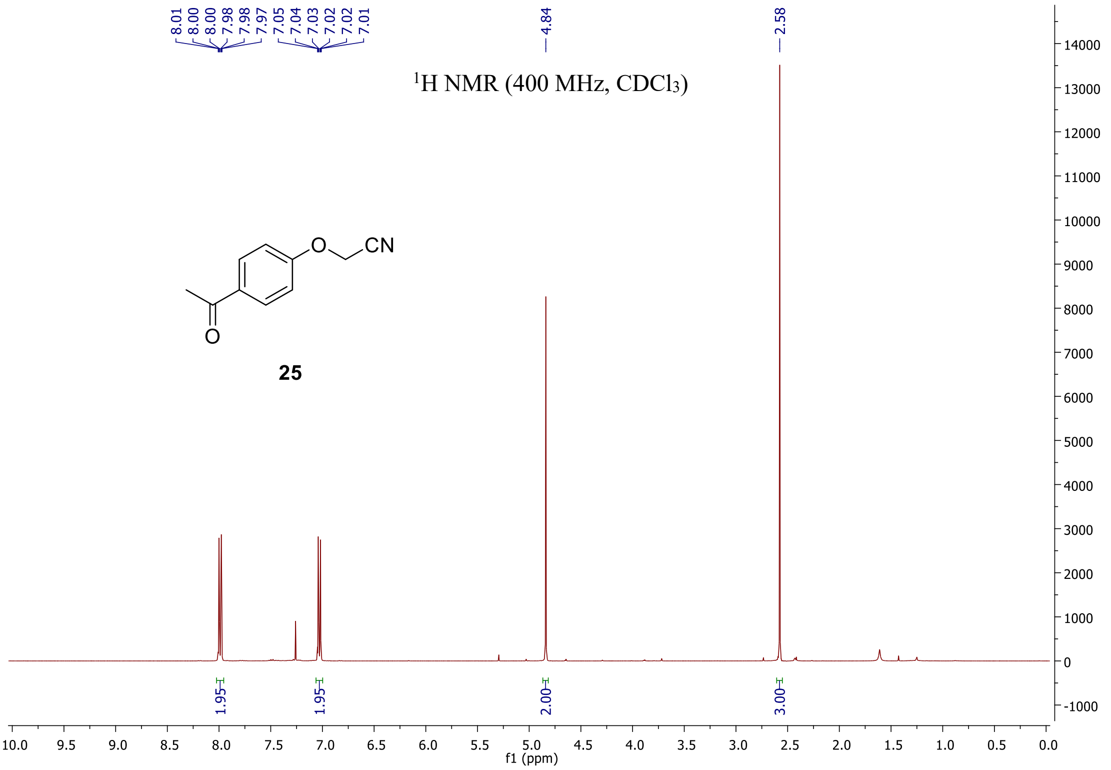

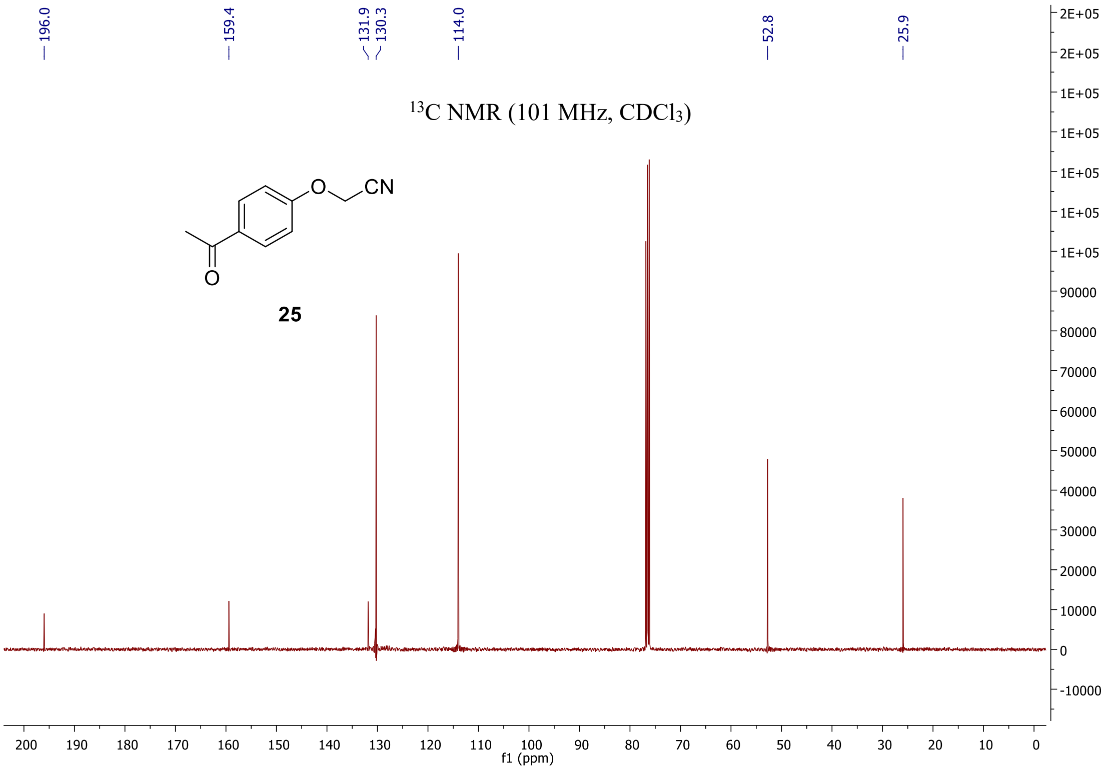

2-(Naphthalen-2-yloxy)acetonitrile (**27**)

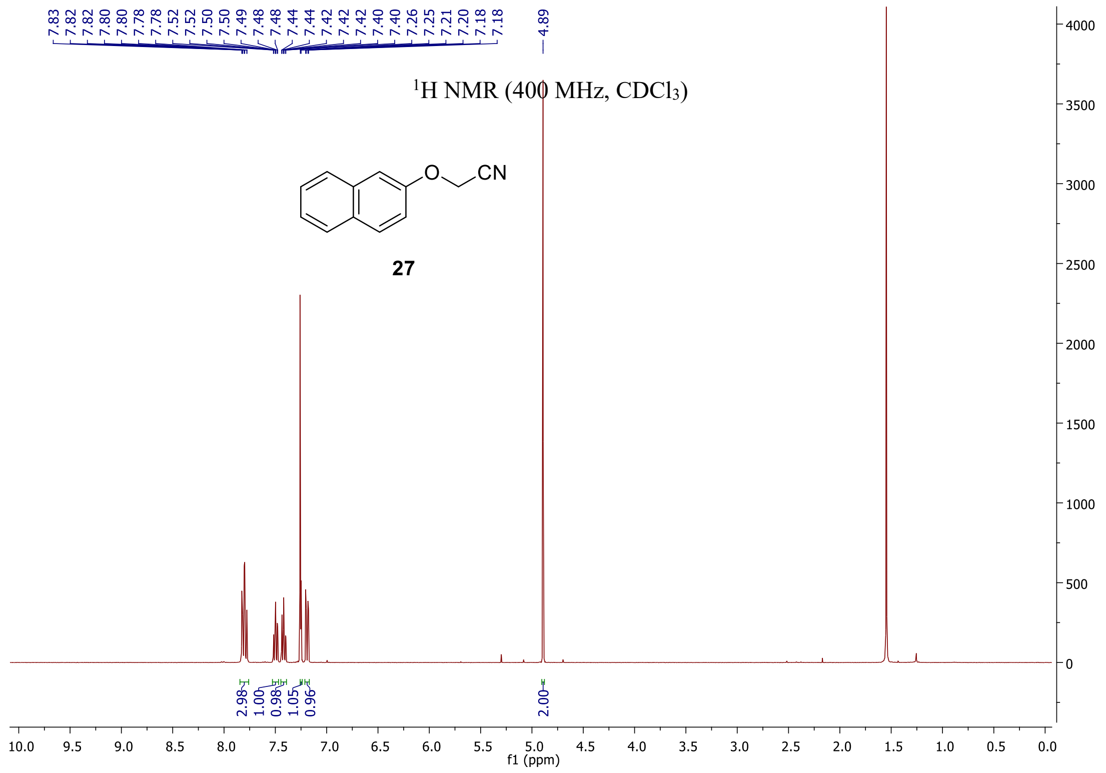

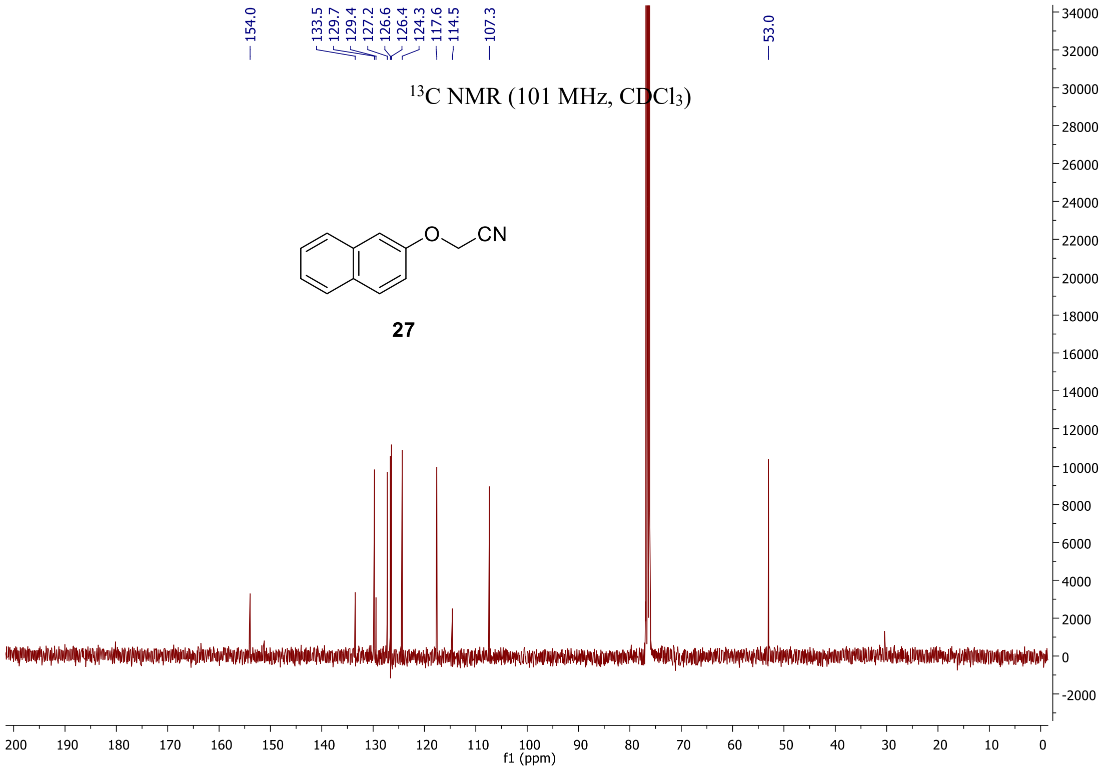

Allylation Products

((4-Phenoxybut-1-en-2-yl)sulfonyl)benzene (**28**)

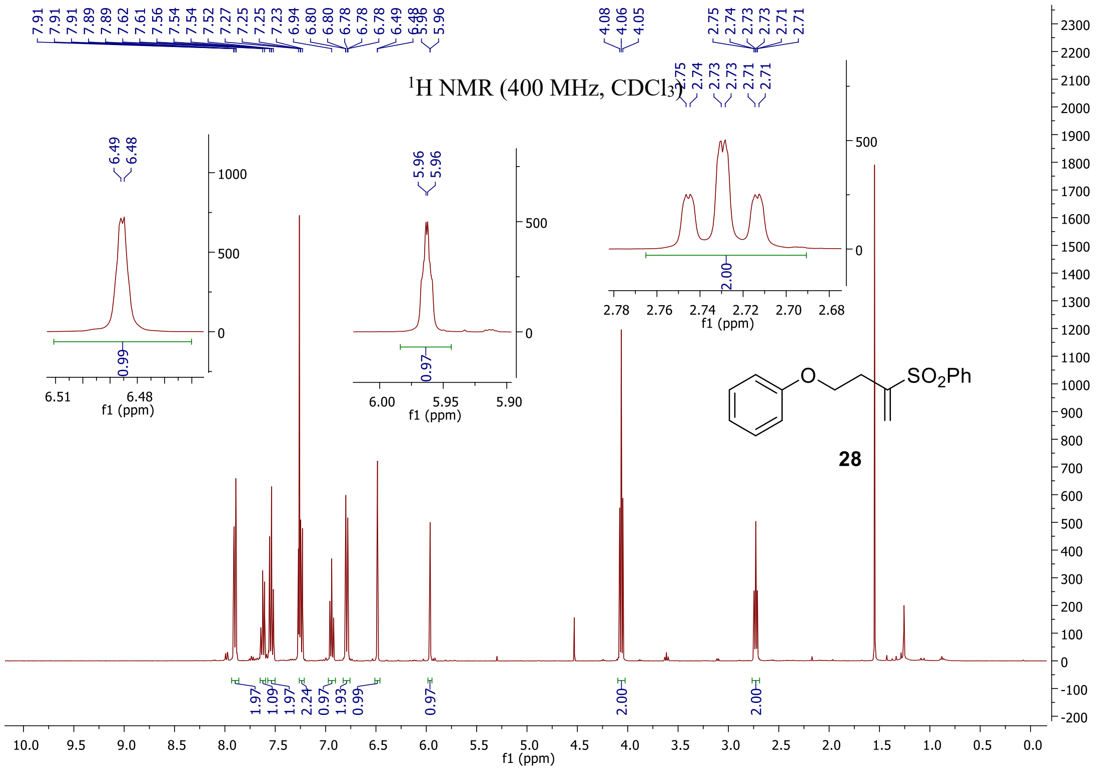

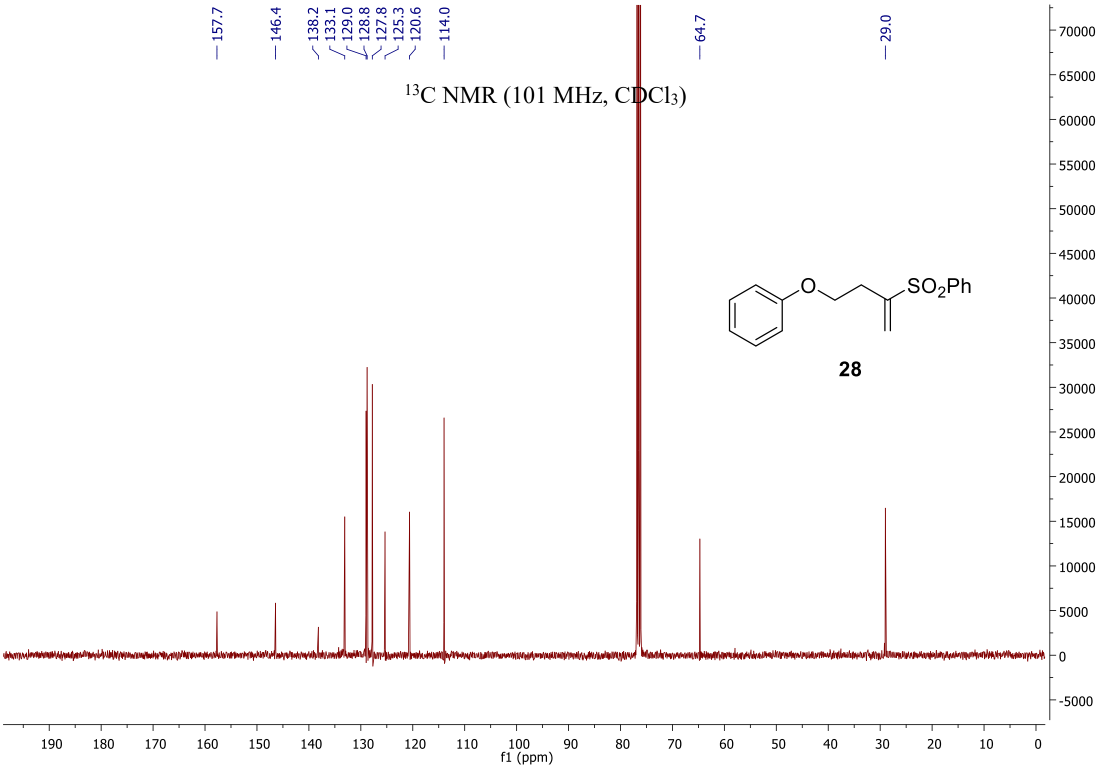

((4-Phenoxypent-1-en-2-yl)sulfonyl)benzene (**29**)

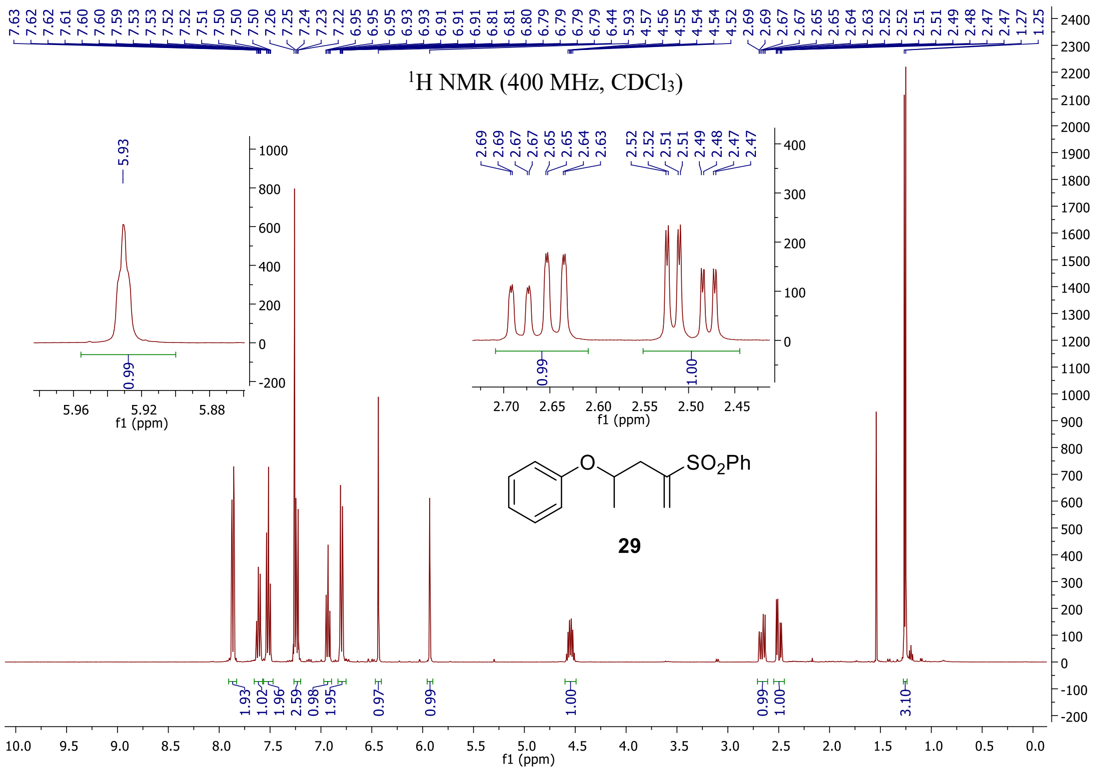

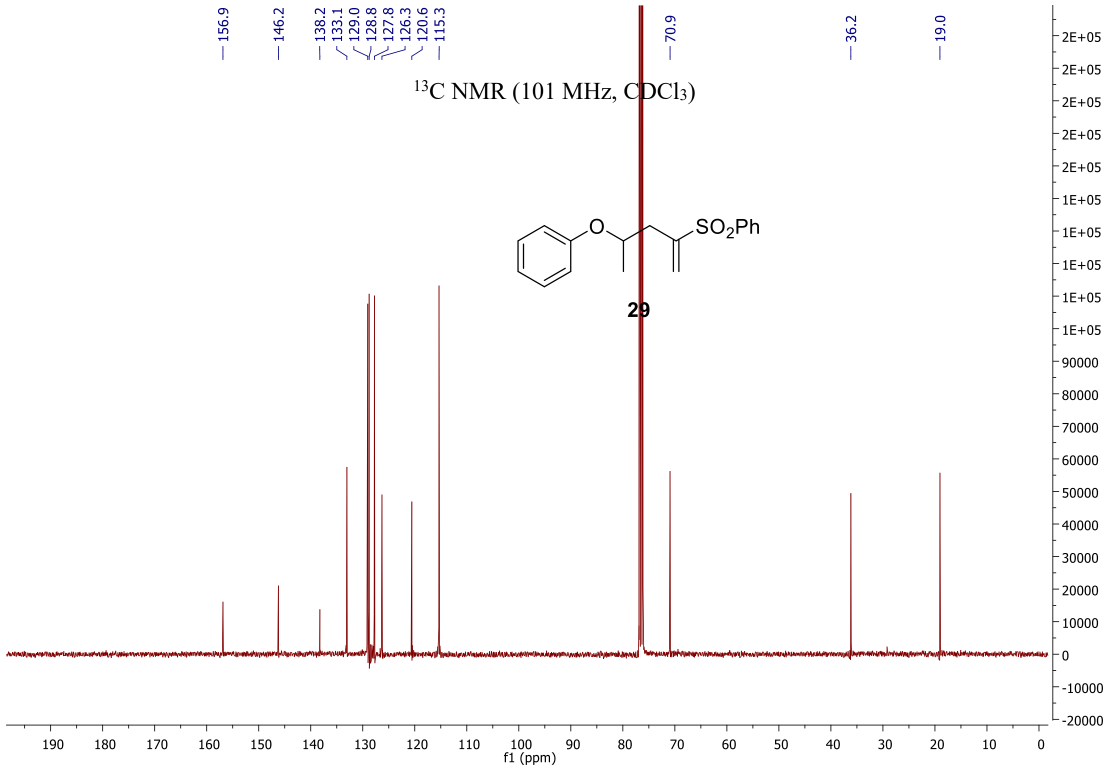

((2-Methyl-4-(phenylsulfonyl)pent-4-en-2-yl)oxy)benzene (**30**)

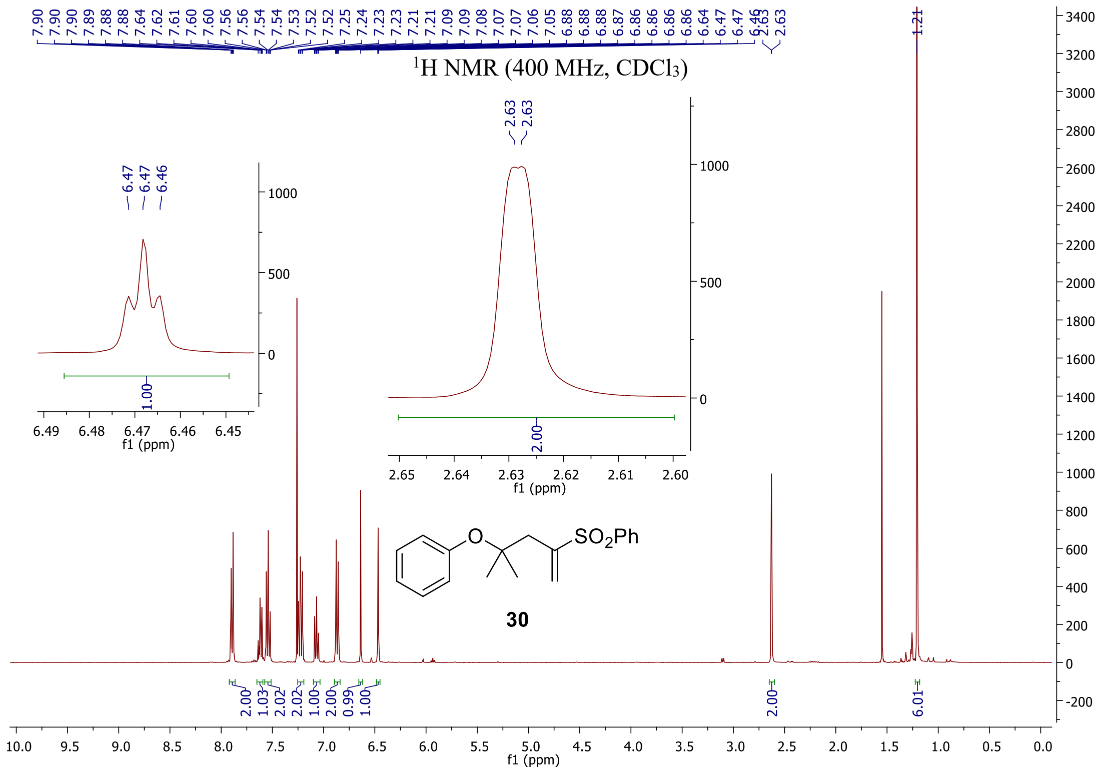

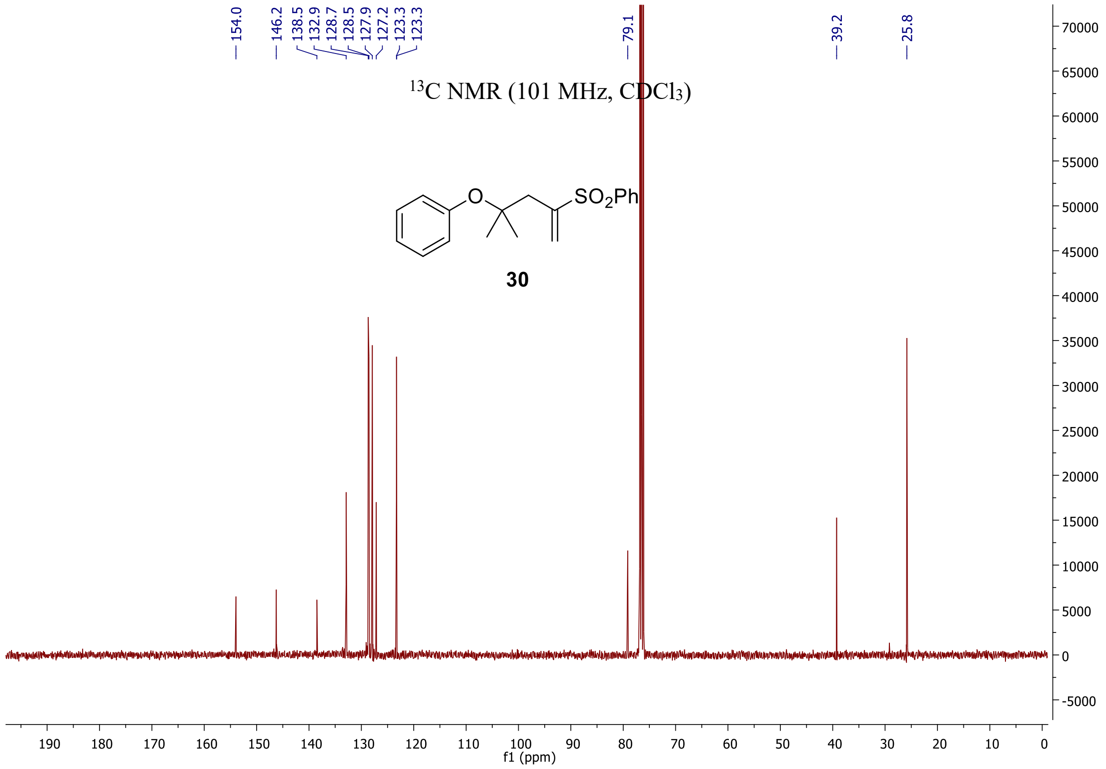

((3-(1-Phenoxycyclopentyl)prop-1-en-2-yl)sulfonyl)benzene (**31**)

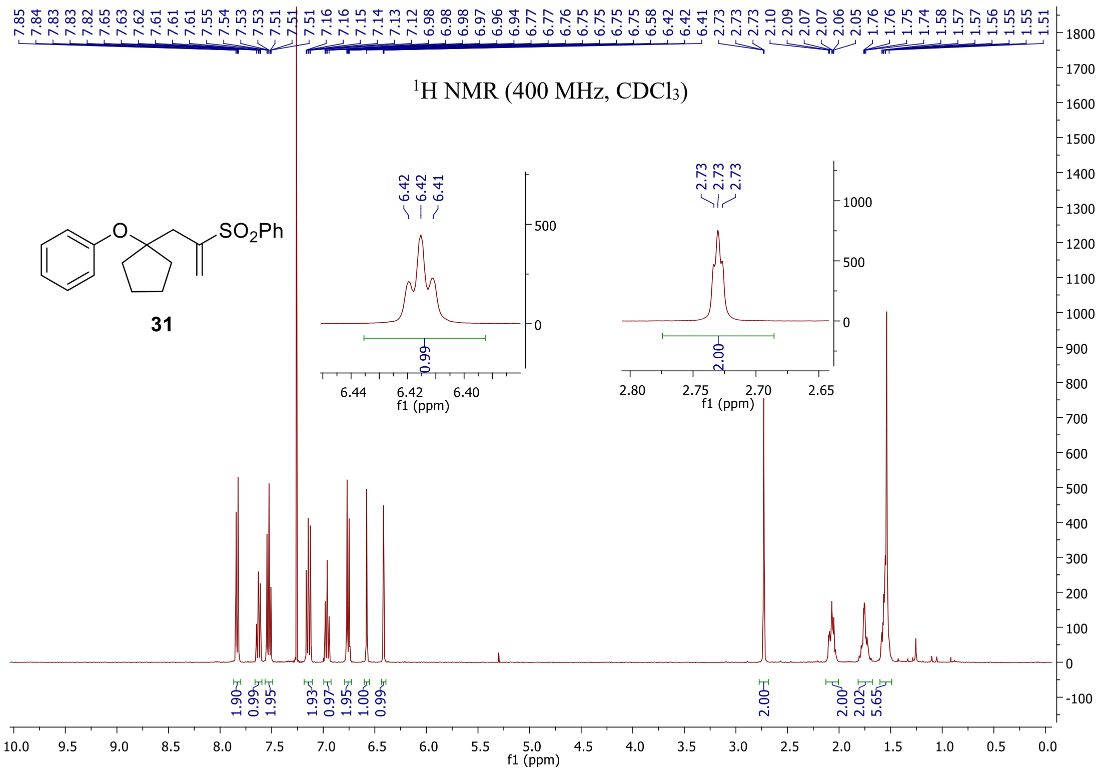

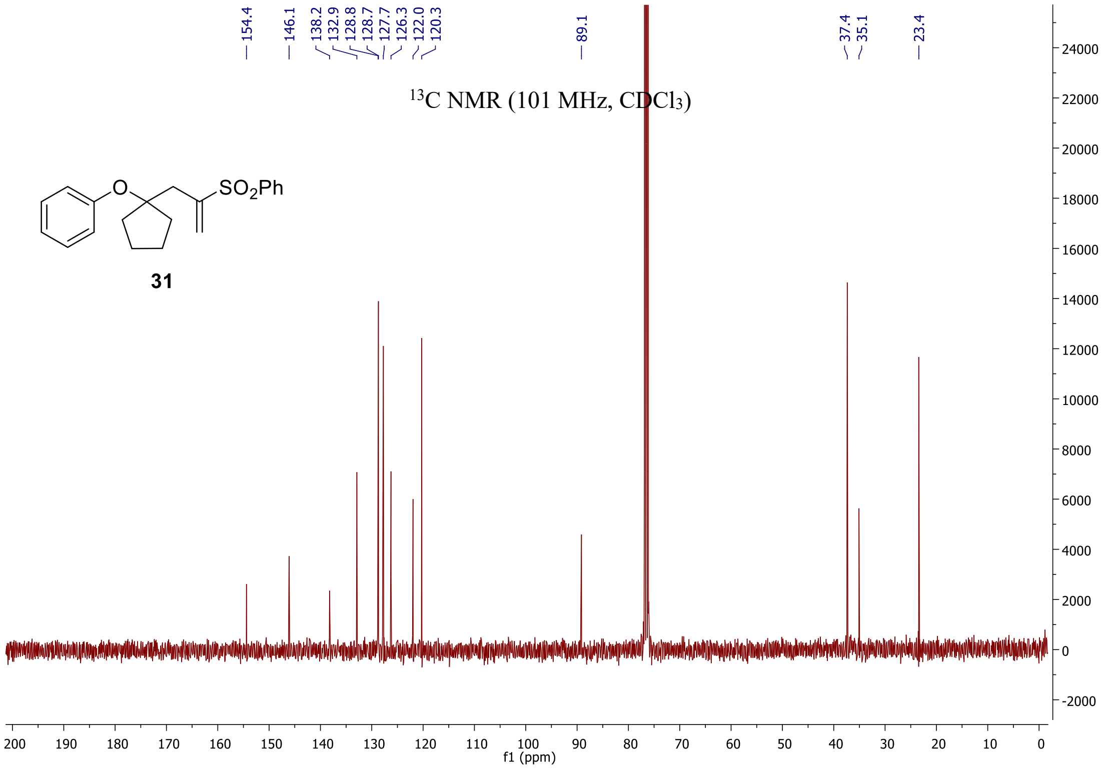

((4-Phenoxynon-1-en-2-yl)sulfonyl)benzene (**32**)

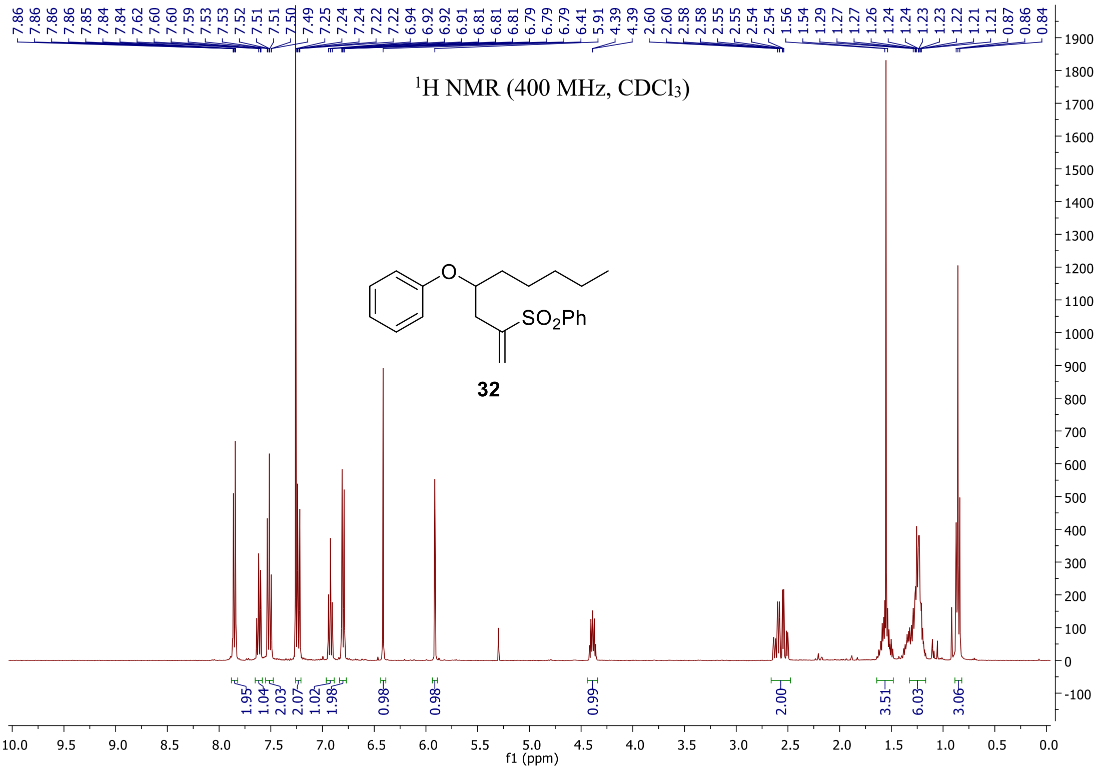

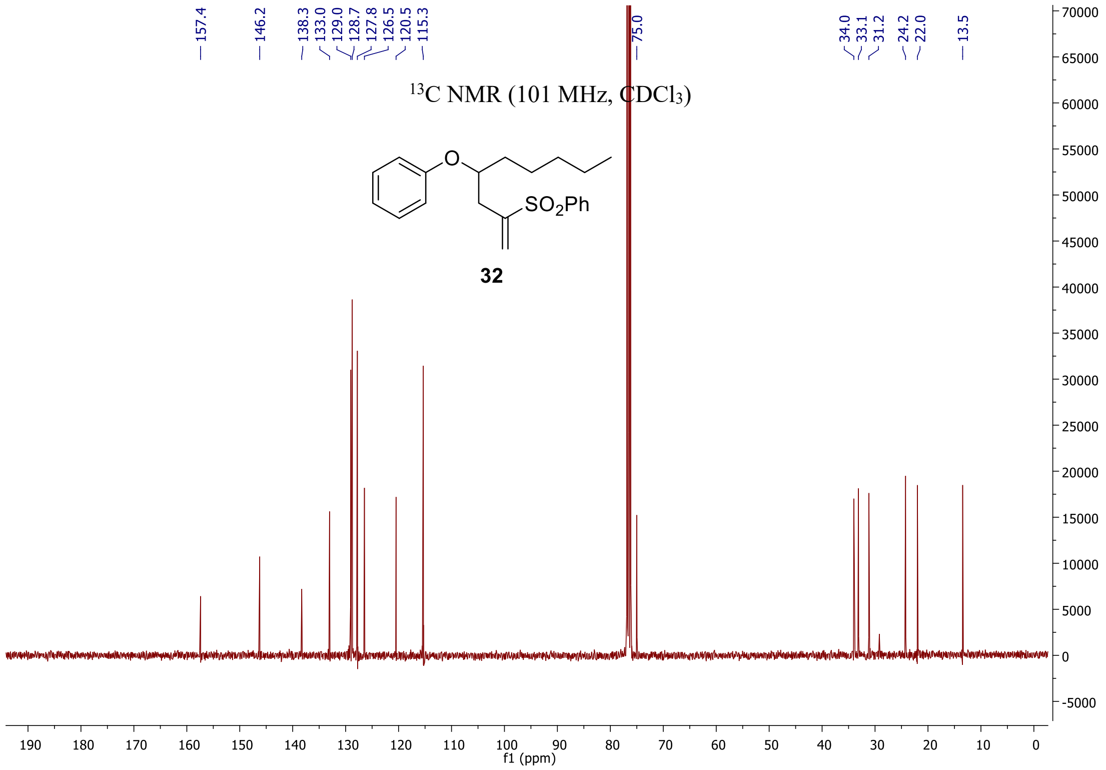

(3-Phenoxy-5-(phenylsulfonyl)hex-5-en-1-yl)benzene (**33**)

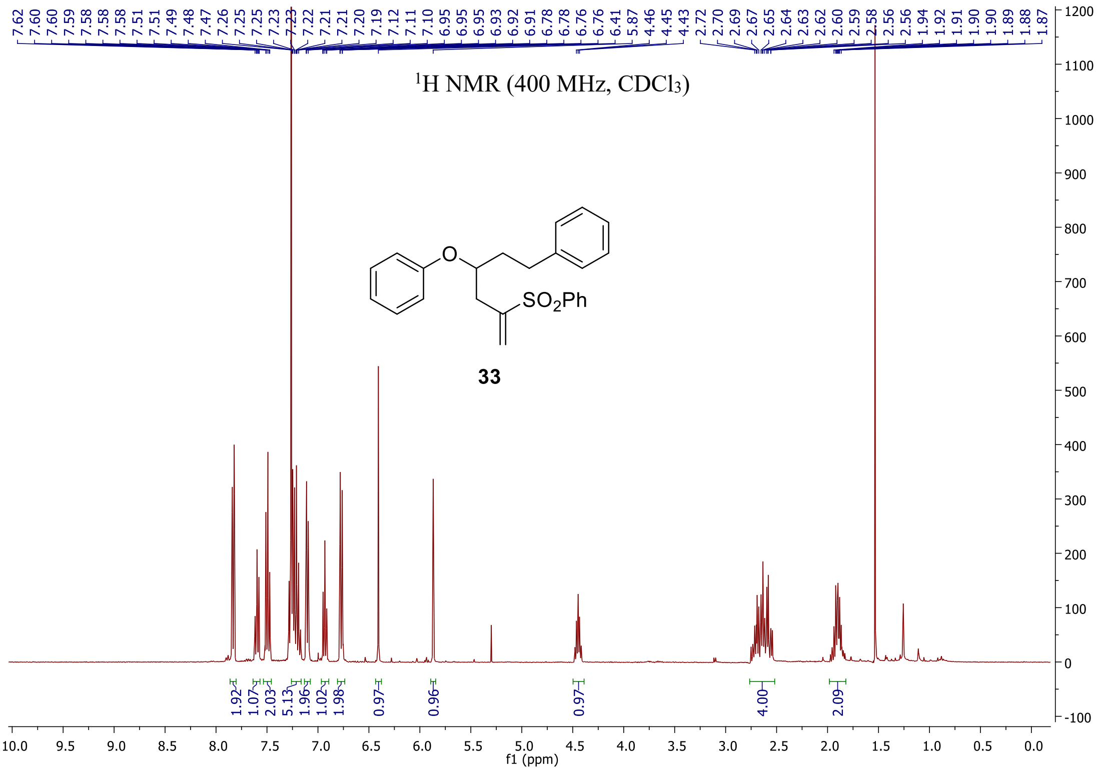

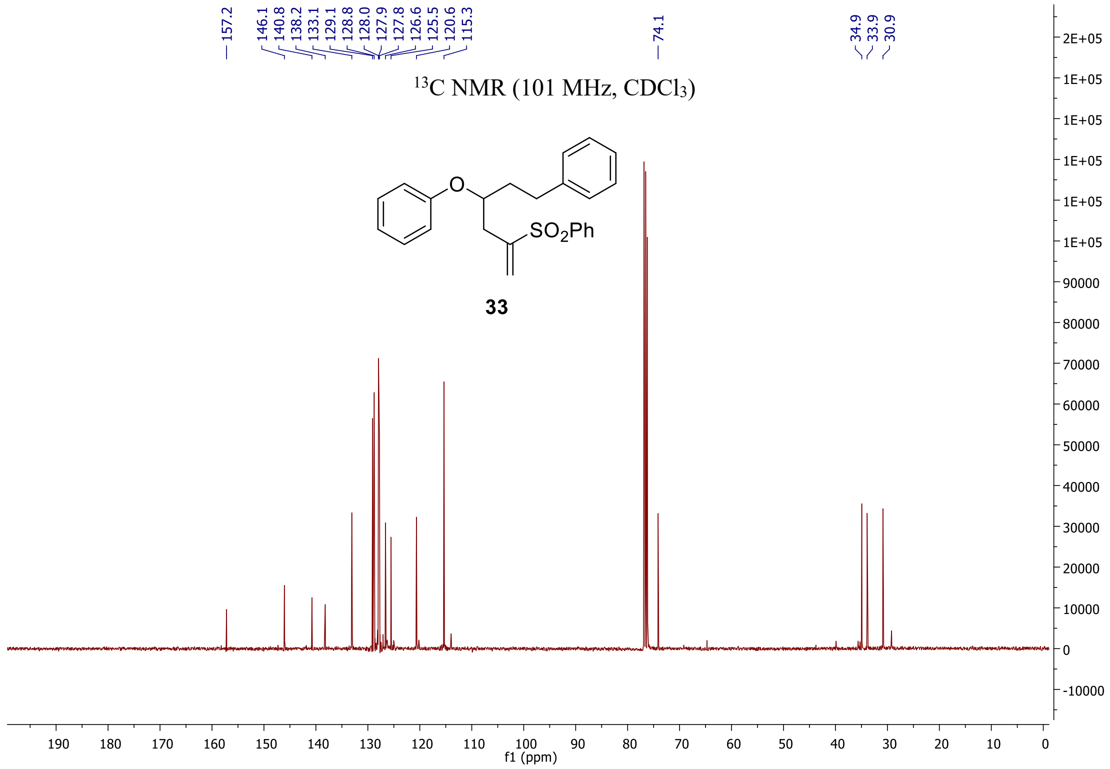

(1-Phenoxy-3-(phenylsulfonyl)but-3-en-1-yl)benzene (**34**)

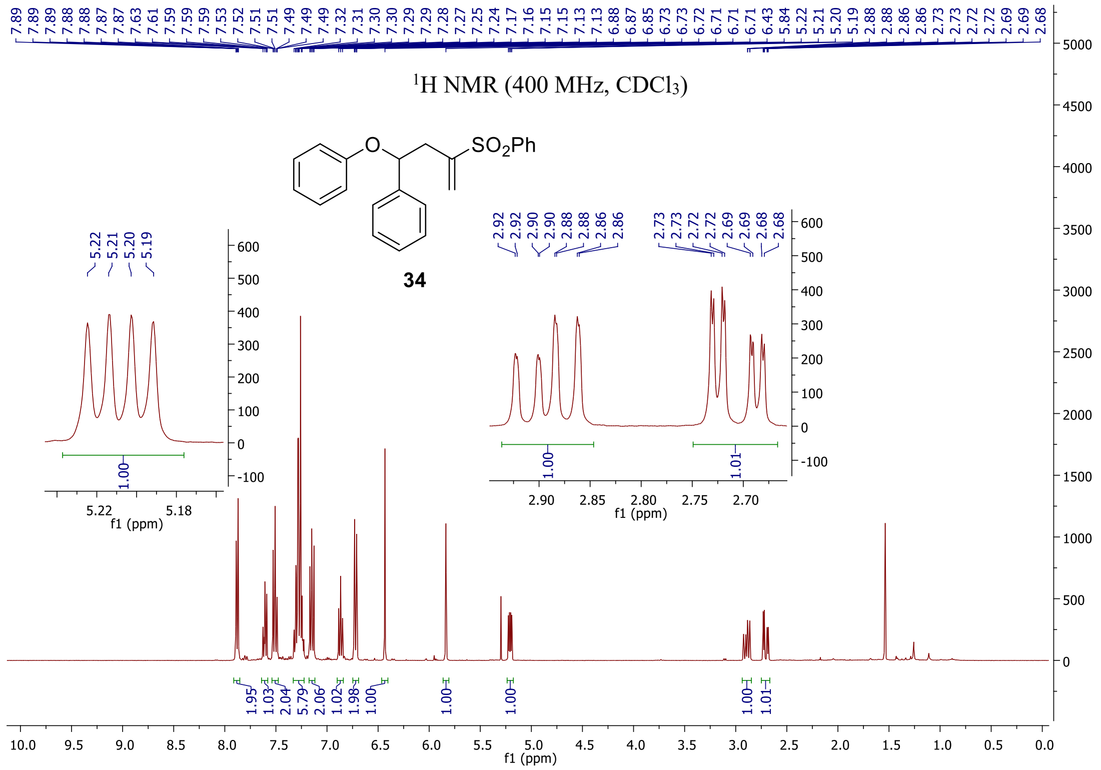

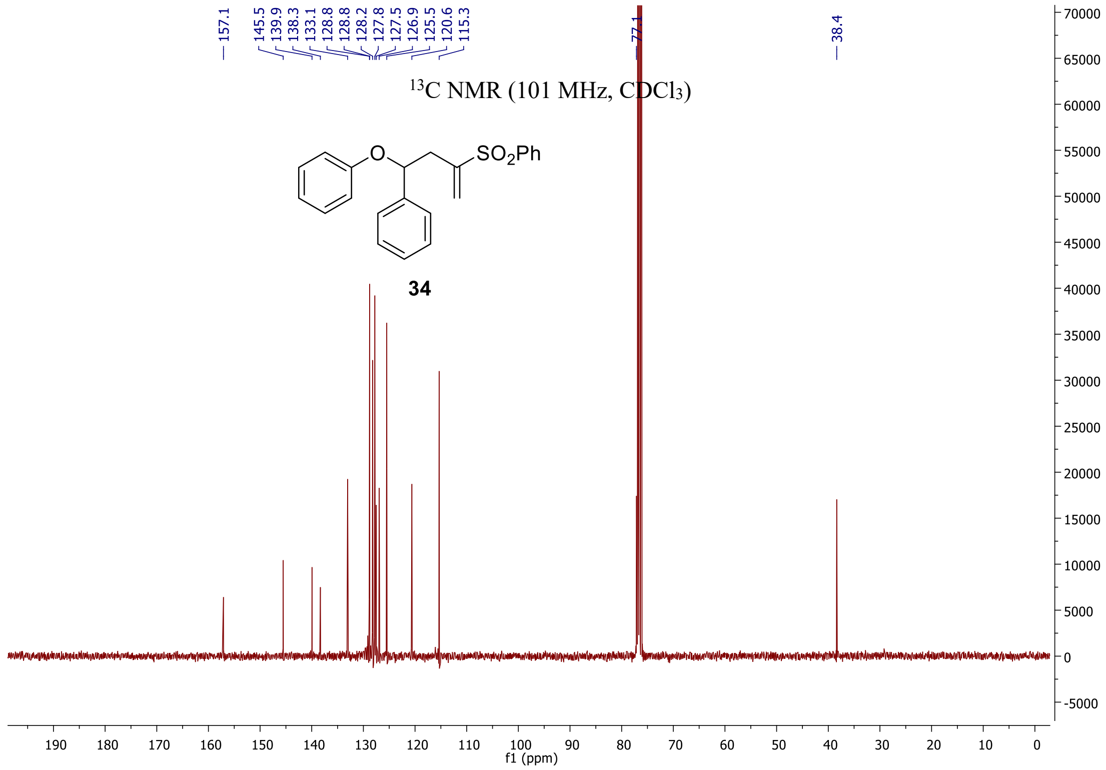

2-(3-Phenoxy-5-(phenylsulfonyl)hex-5-en-1-yl)isoindoline-1,3-dione (**35**)

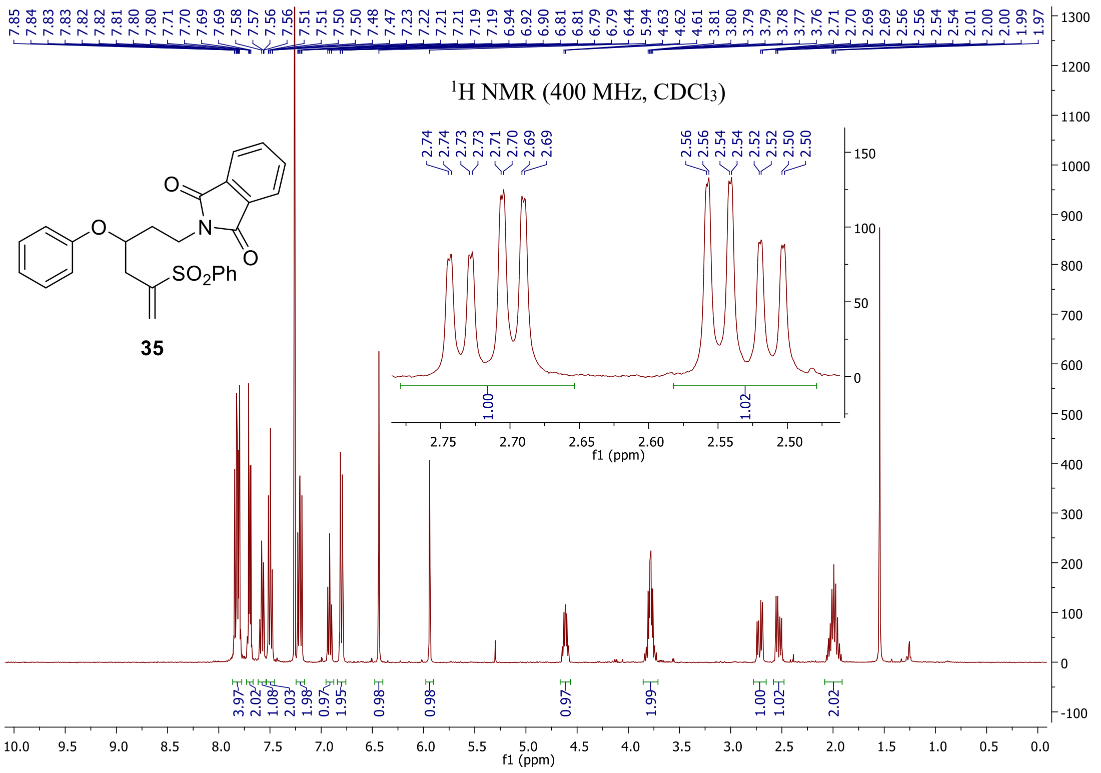

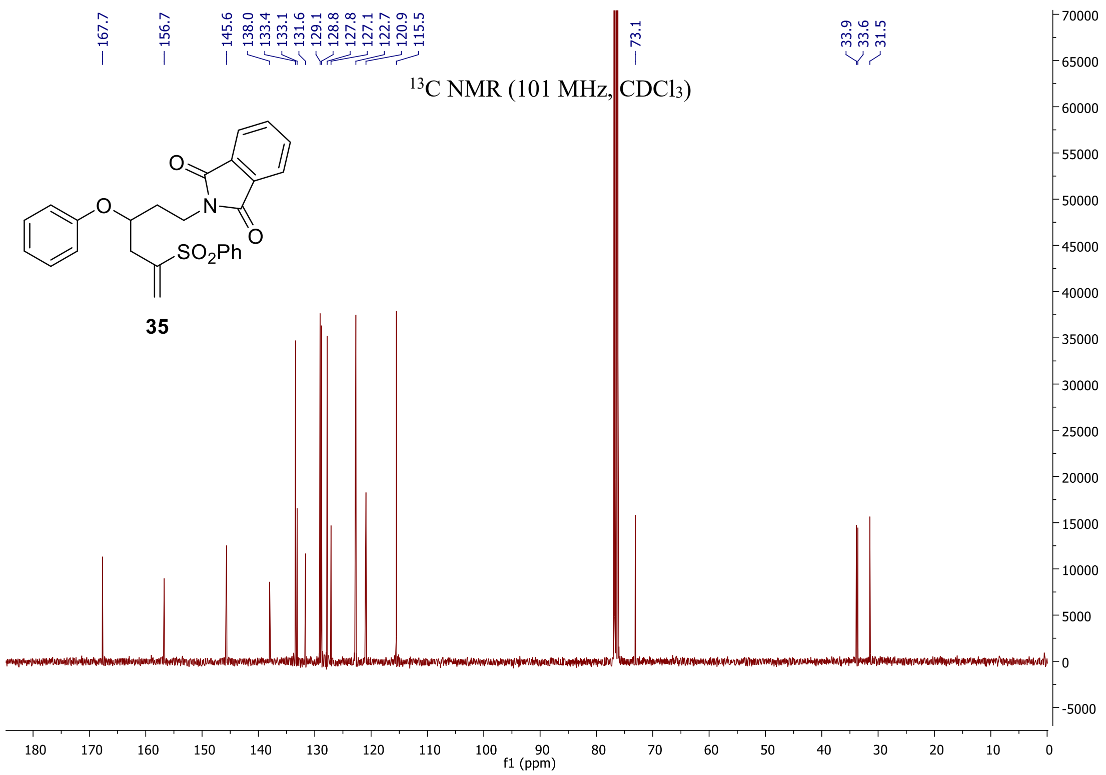

1-Methyl-3-((3-(phenylsulfonyl)but-3-en-1-yl)oxy)benzene (**36**)

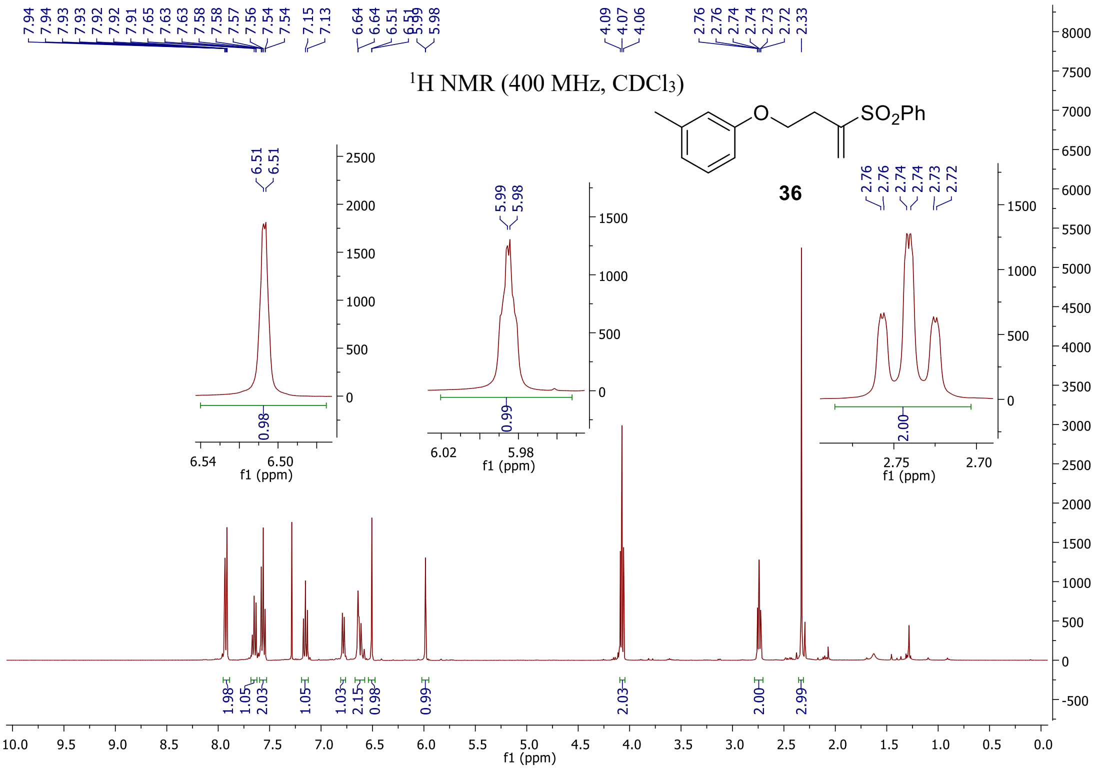

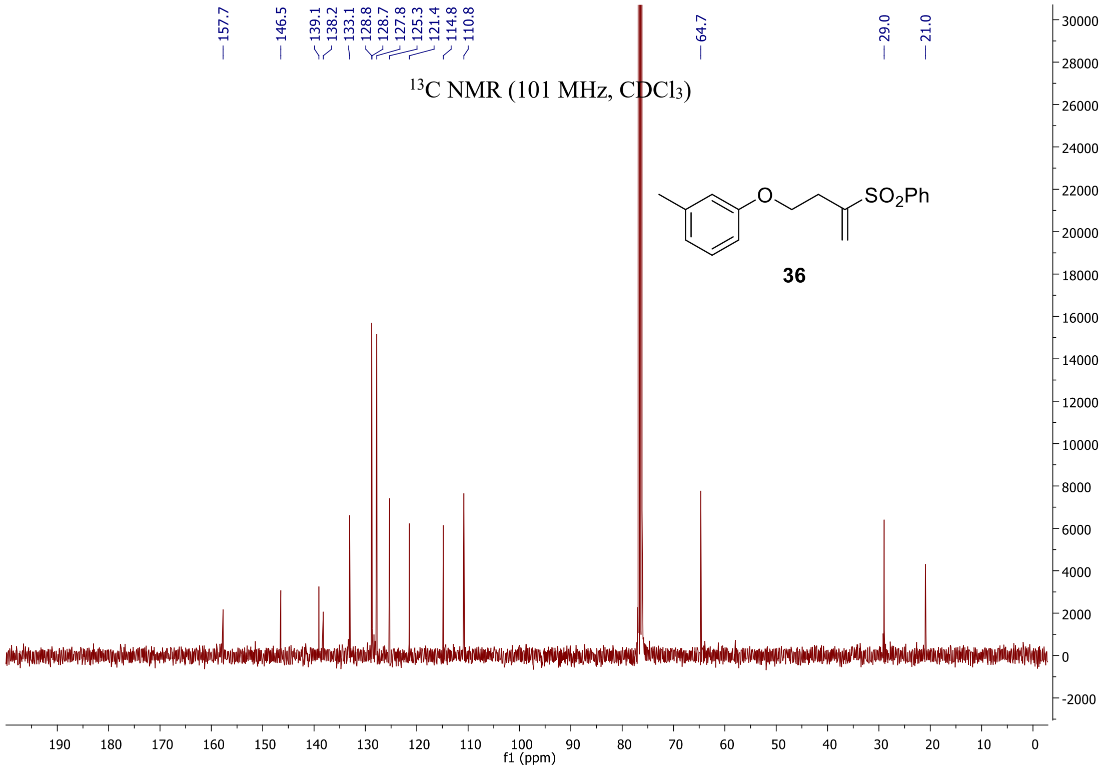

1-(*tert*-Butyl)-4-((3-(phenylsulfonyl)but-3-en-1-yl)oxy)benzene (**37**)

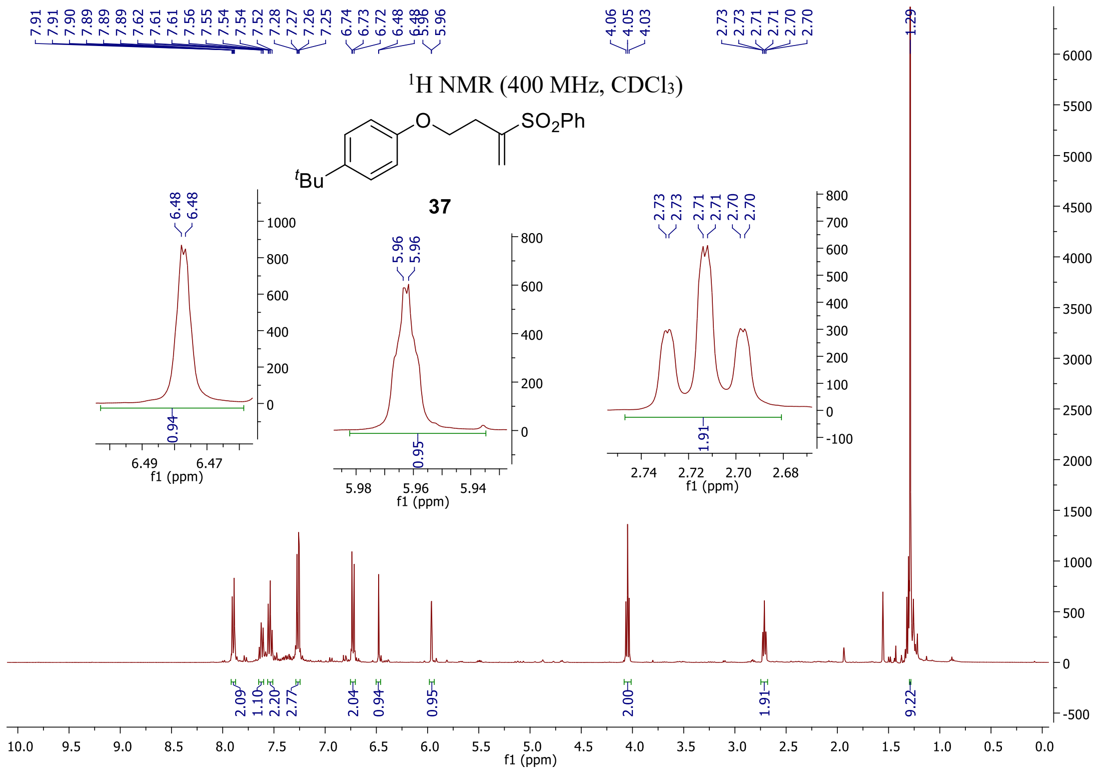

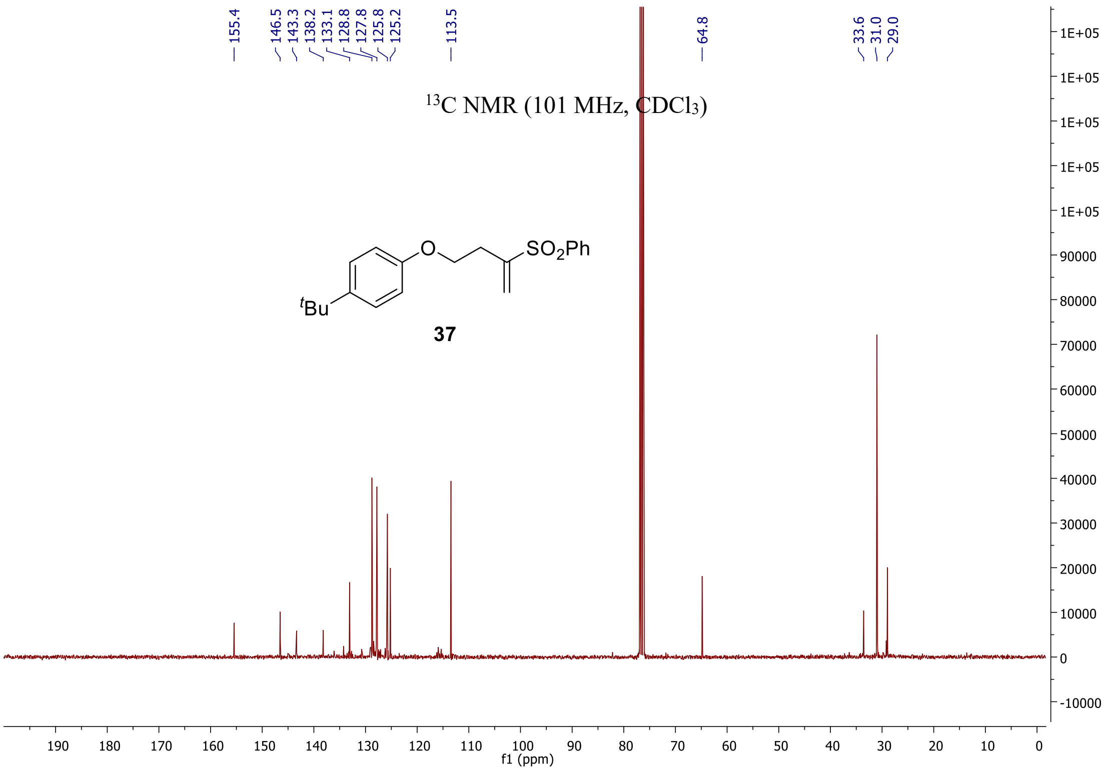

(4-(4-(*tert*-Butyl)phenoxy)hepta-1,6-diene-2,6-diyldisulfonyl)dibenzene (**38**)

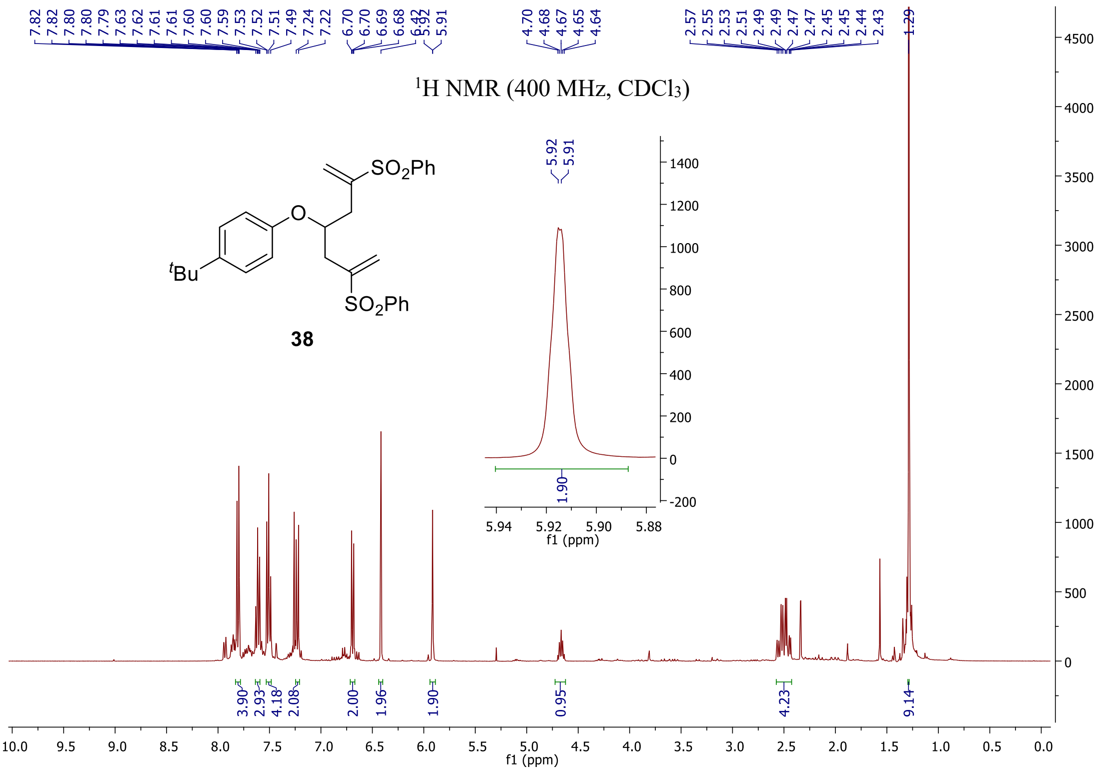

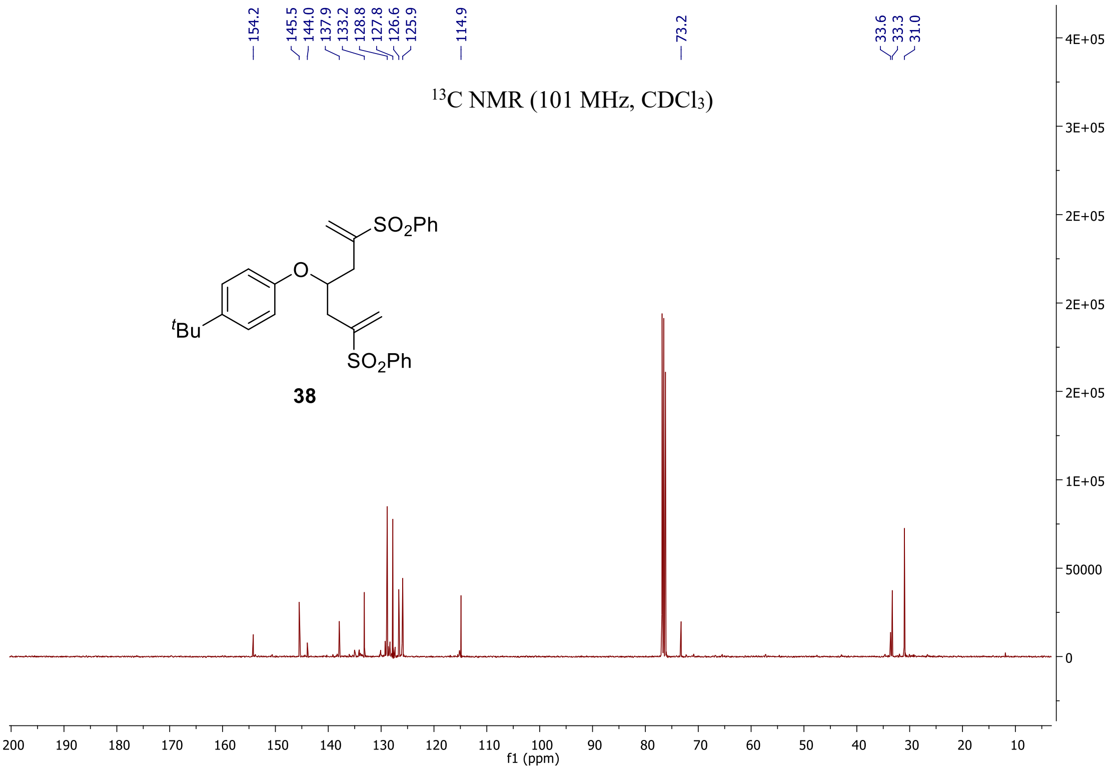

1-Fluoro-4-((3-(phenylsulfonyl)but-3-en-1-yl)oxy)benzene (**39**)

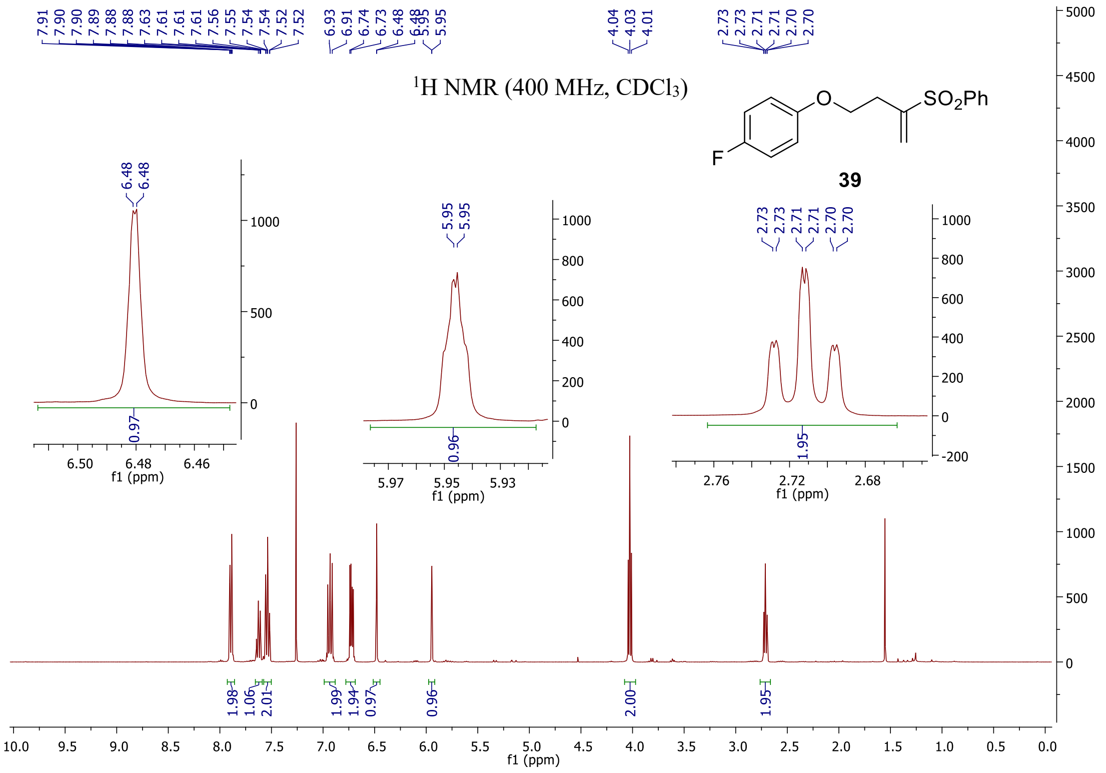

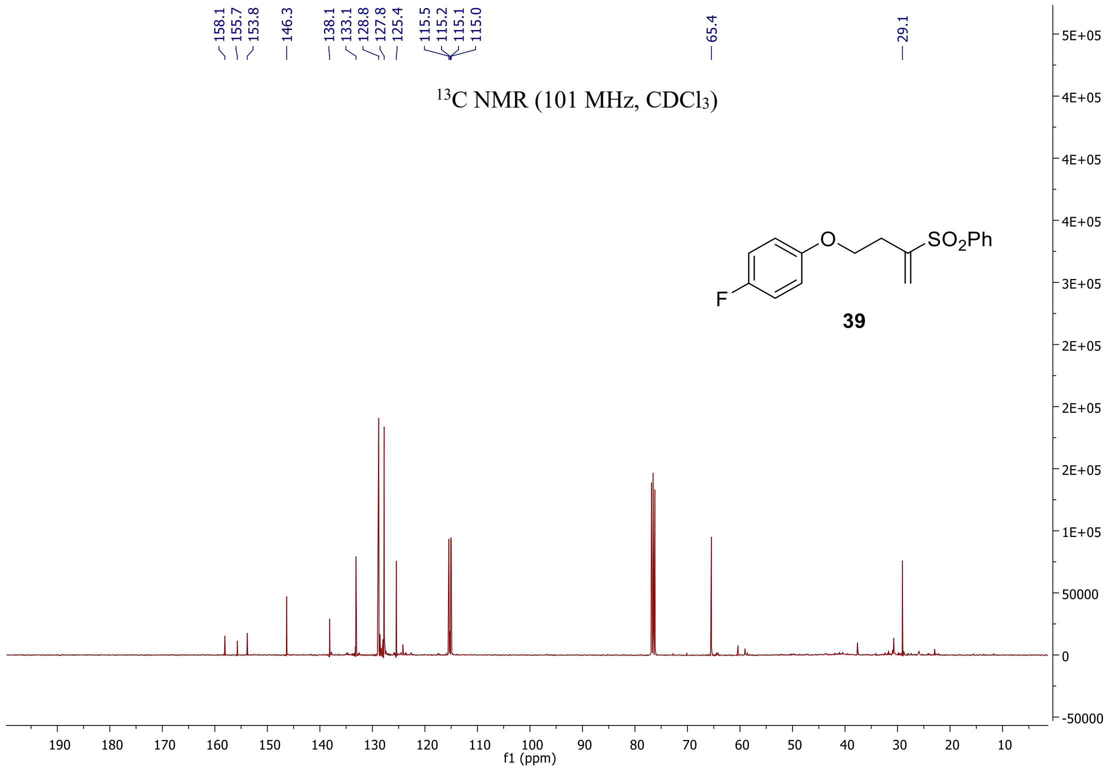

1-Chloro-4-((3-(phenylsulfonyl)but-3-en-1-yl)oxy)benzene (**40**)

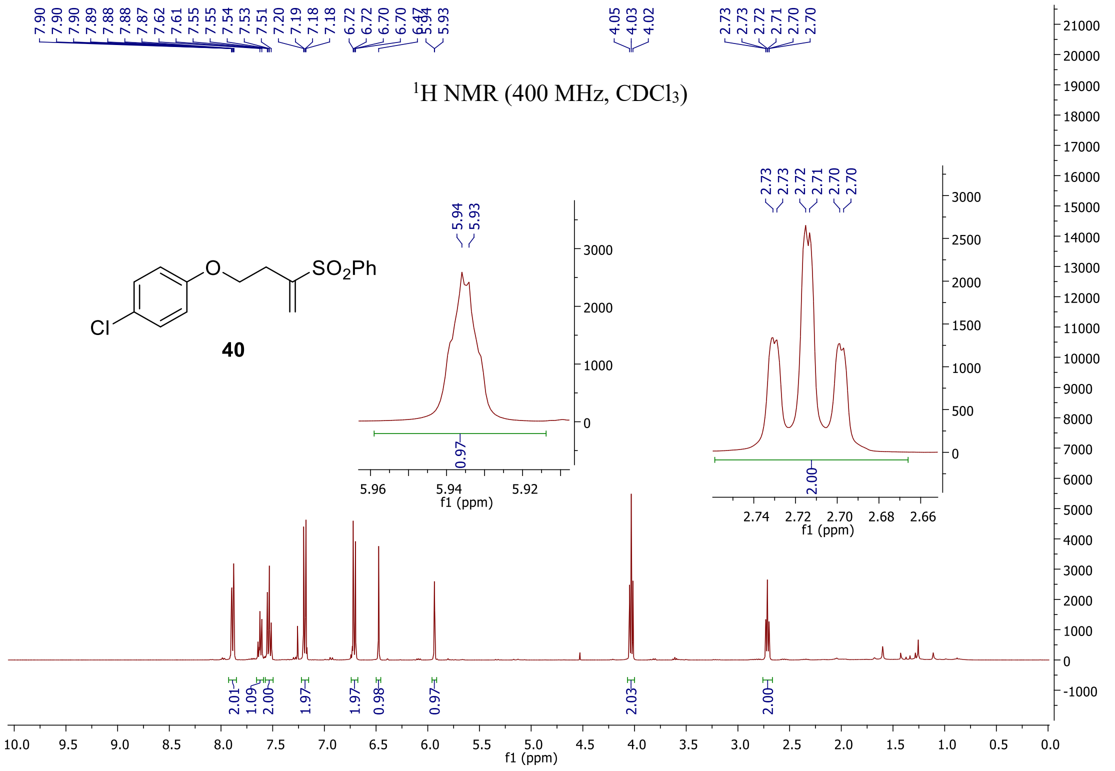

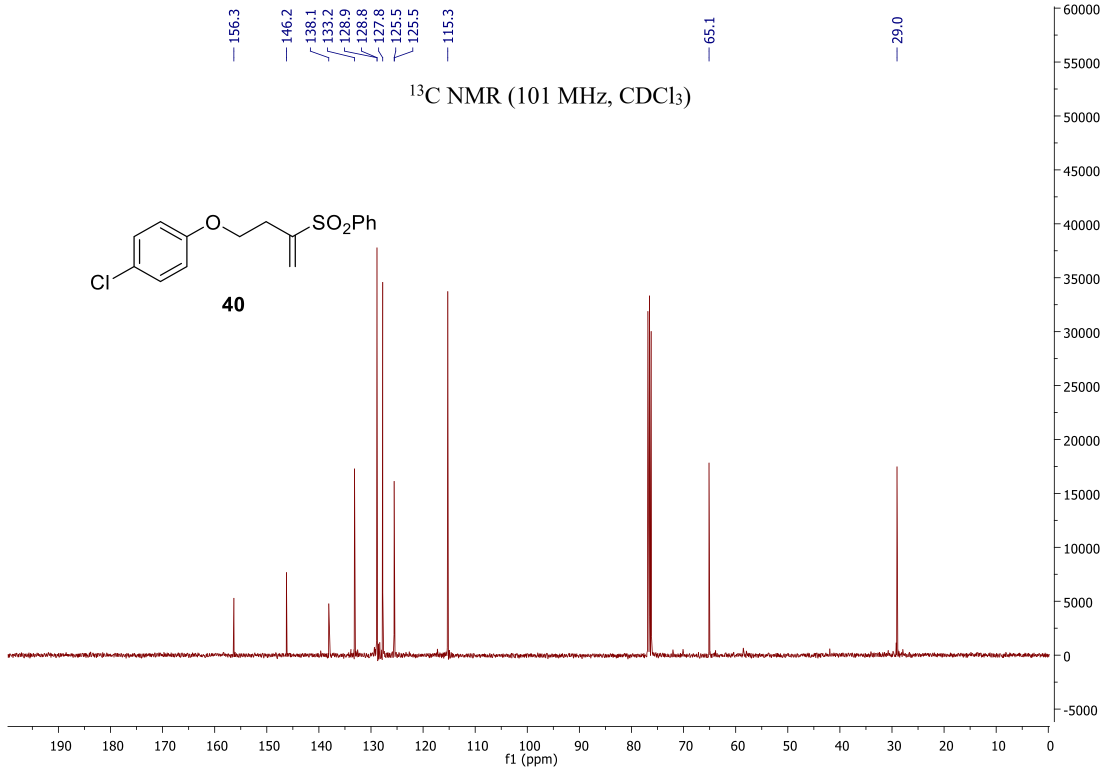

Ethyl 4-methyl-2-methylene-4-phenoxypentanoate (**41**)

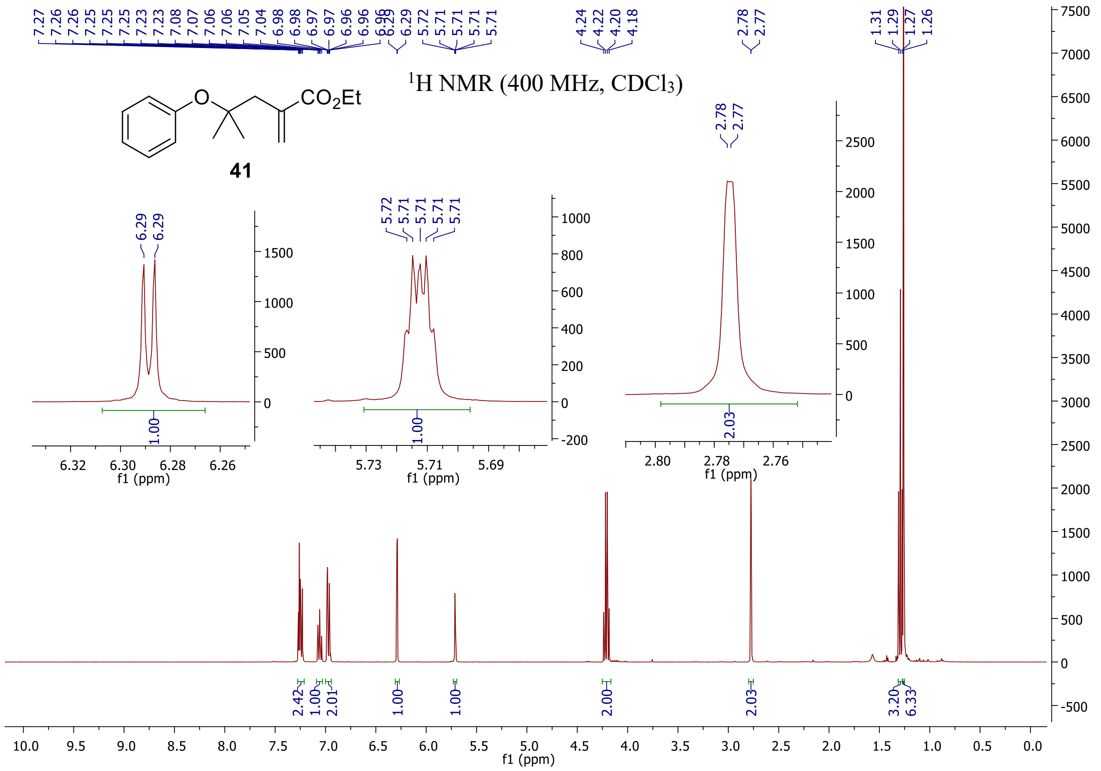

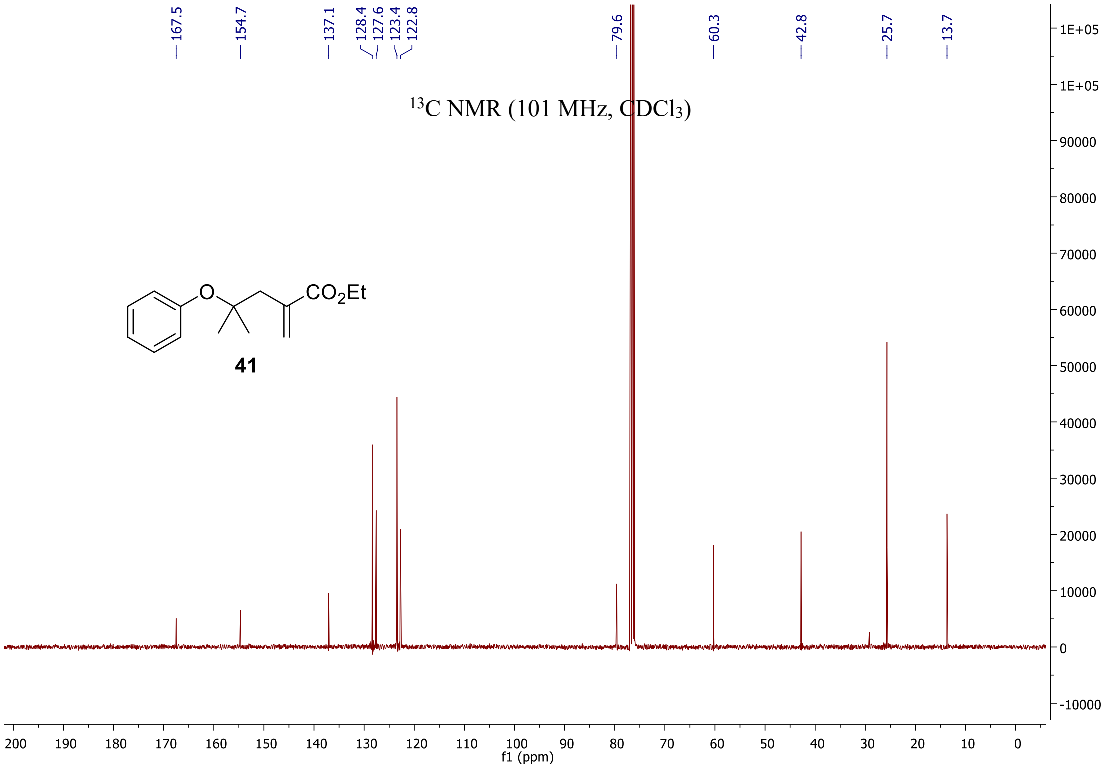

4-Methyl-2-methylene-4-phenoxy-1-phenylpentan-1-one (**42**)

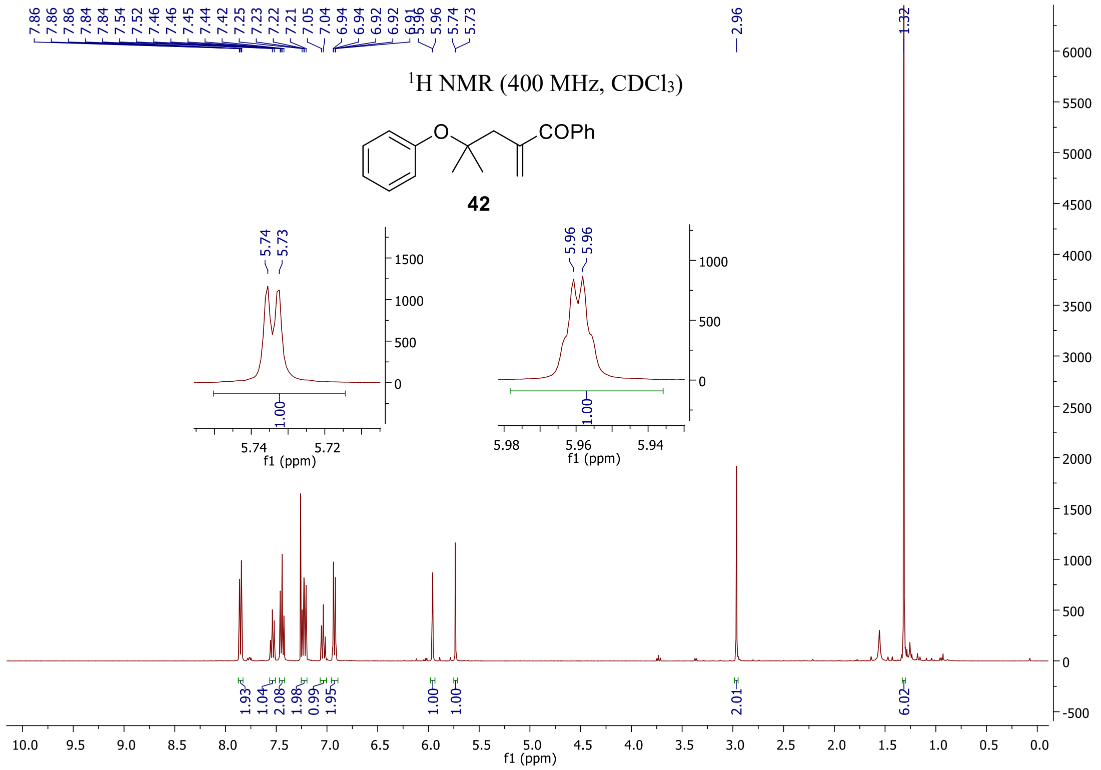

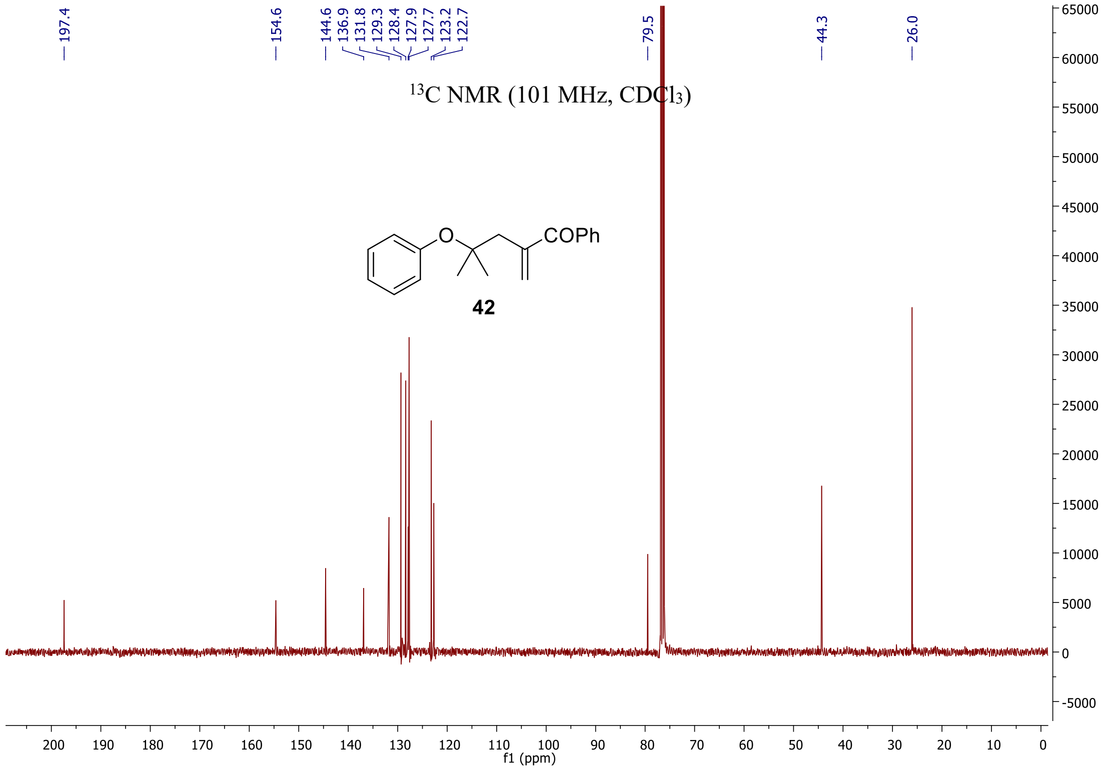

Further Functionalisation Products 44 and 46

1-Fluoro-4-((5-methyl-5-phenoxy-3-(phenylsulfonyl)hexyl)oxy)benzene (**44**)

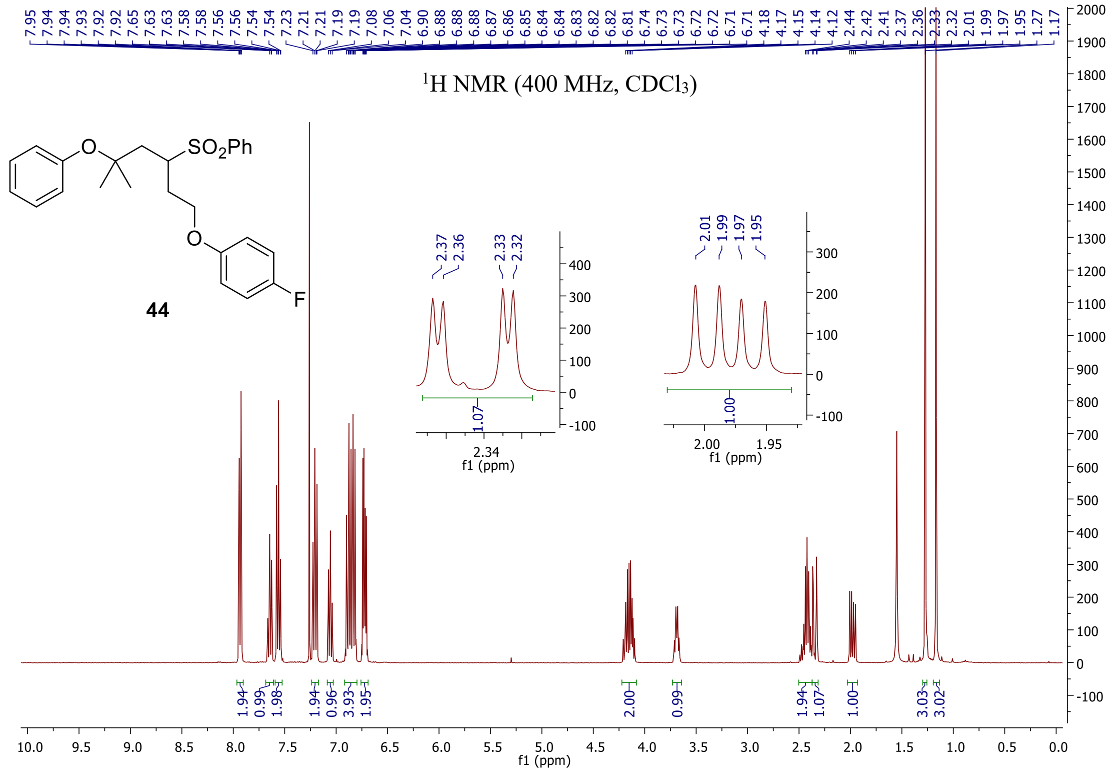

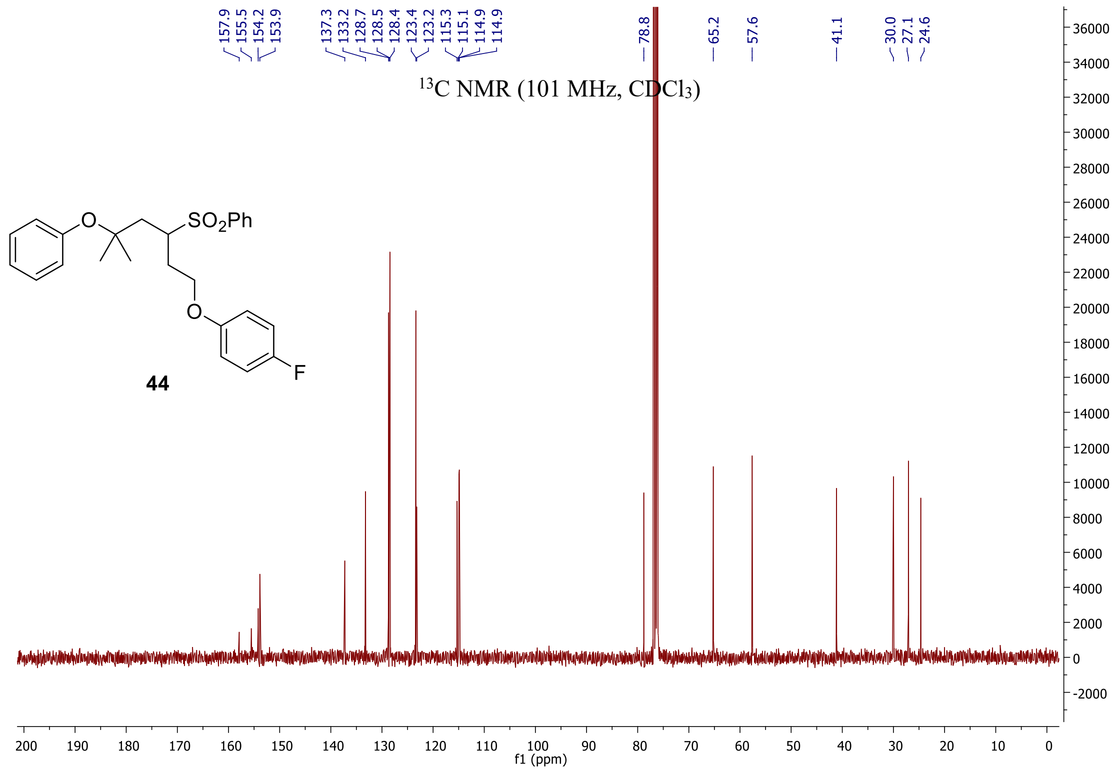

2-(3,7-Diphenoxy-5-(phenylsulfonyl)octyl)isoindoline-1,3-dione (**46**)

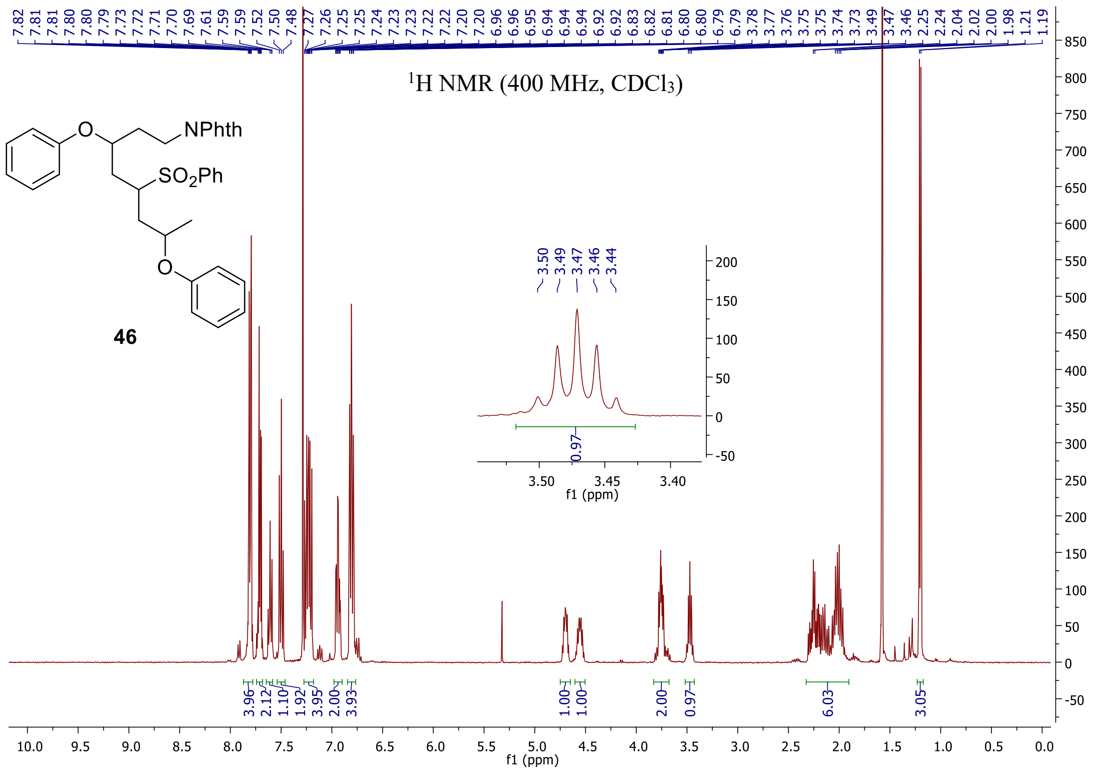

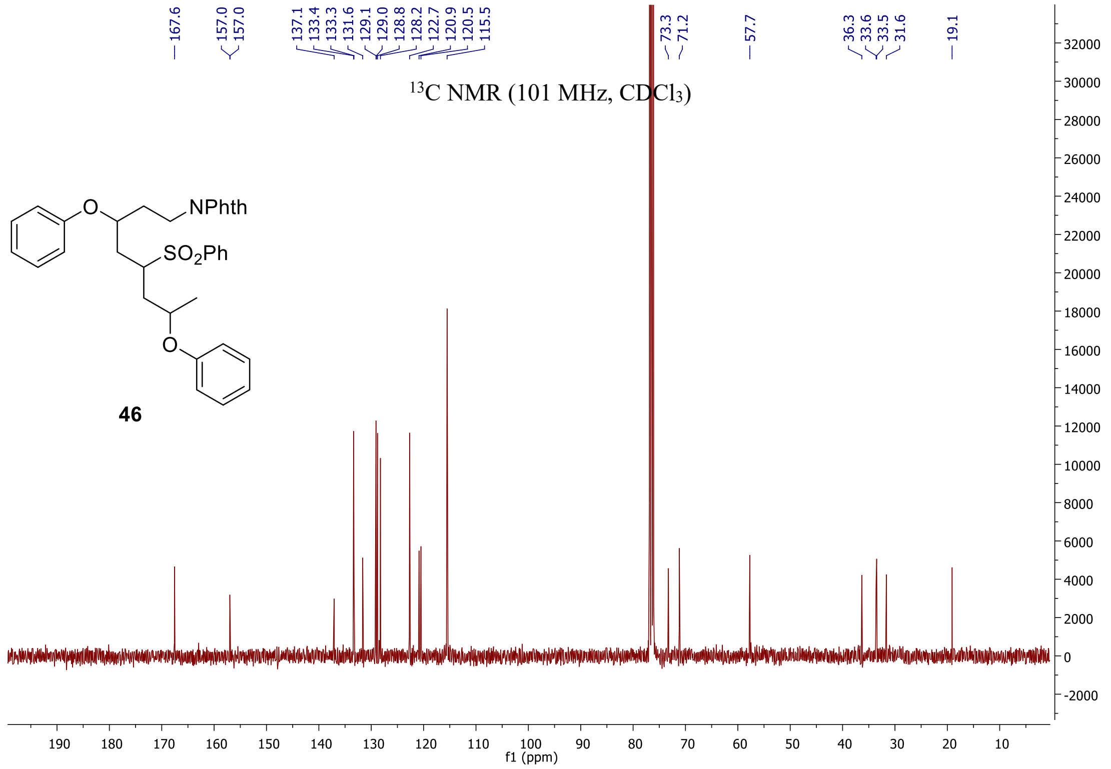

Appendix 2: ^{1}H and ^{13}C NMR Spectra of Complex Mixtures of Diastereomers of Compound 46

Complex Mixture 1

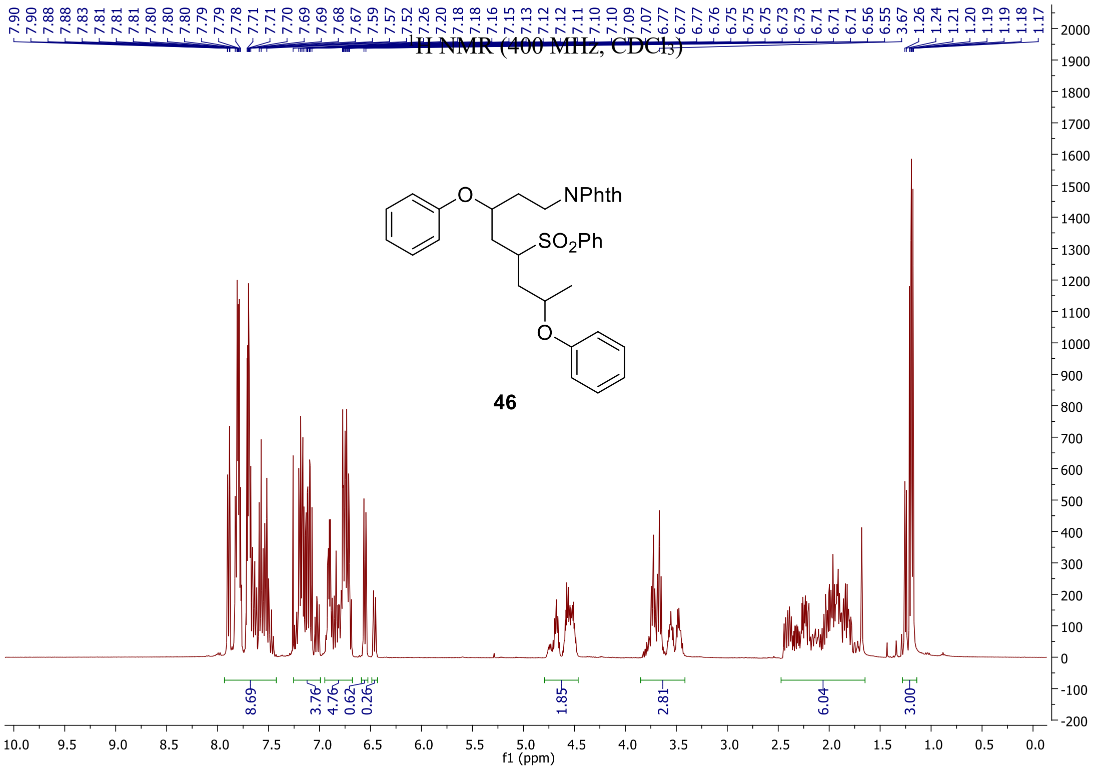

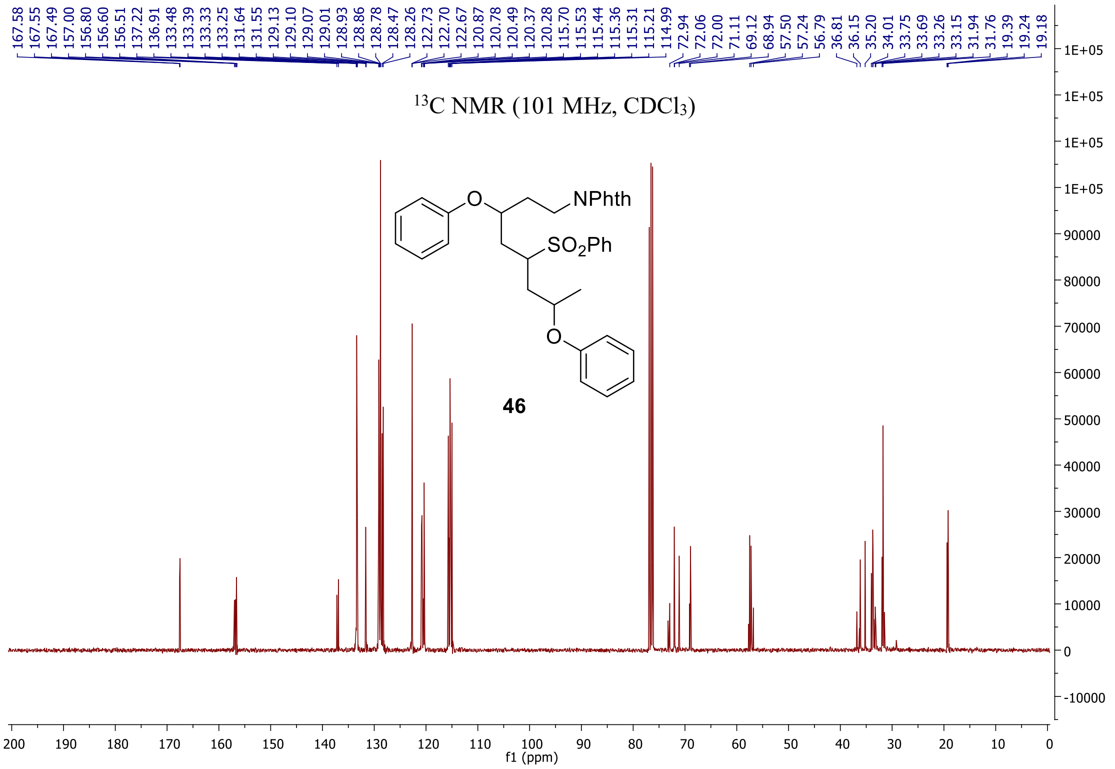

Complex Mixture 2

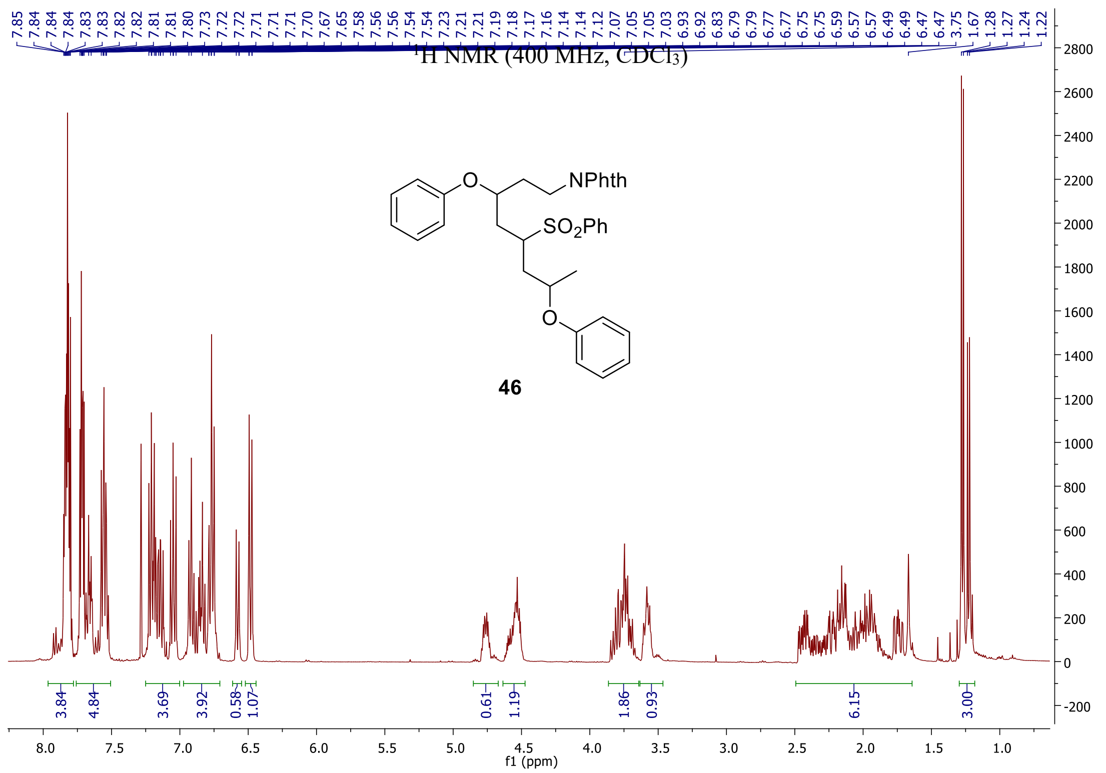

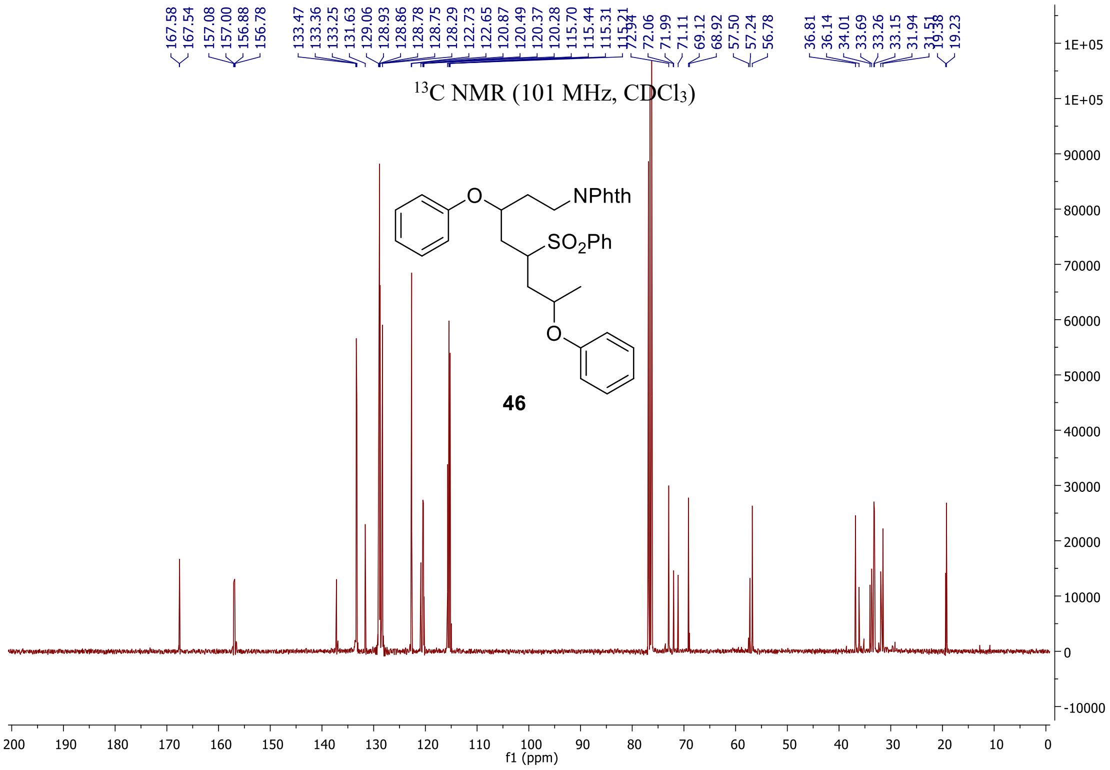

Appendix 3: LCMS Data

Complex Mixture 1 of Product 46 – (a) Positive MS scan, **(b)** Negative MS scan, **(c)** UV trace, **(d)** Expansion of +ve MS scan, **(e)** MS trace from +ve scan, **(f)** Expansion of -ve MS scan, **(g)** MS trace from -ve scan

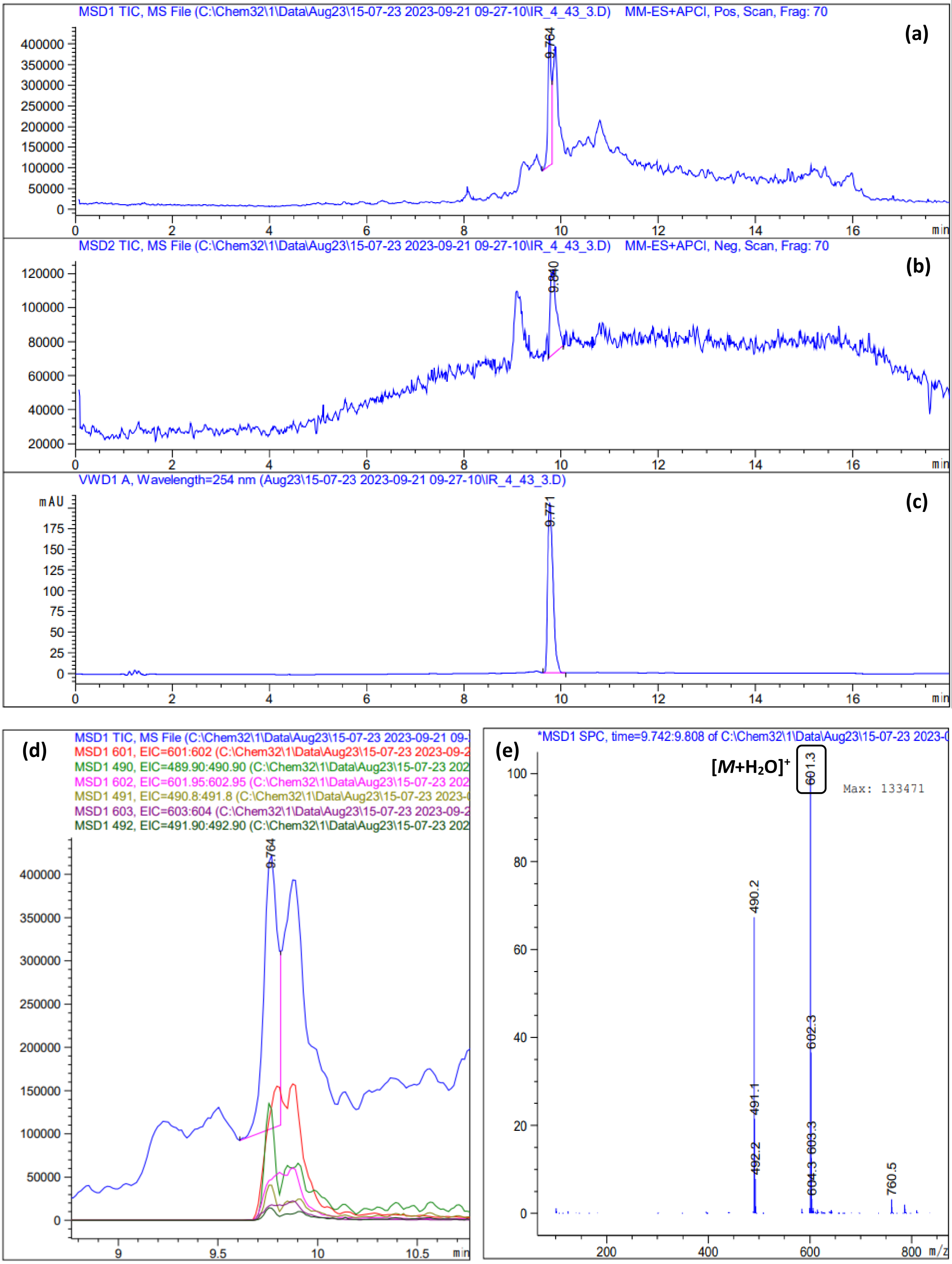

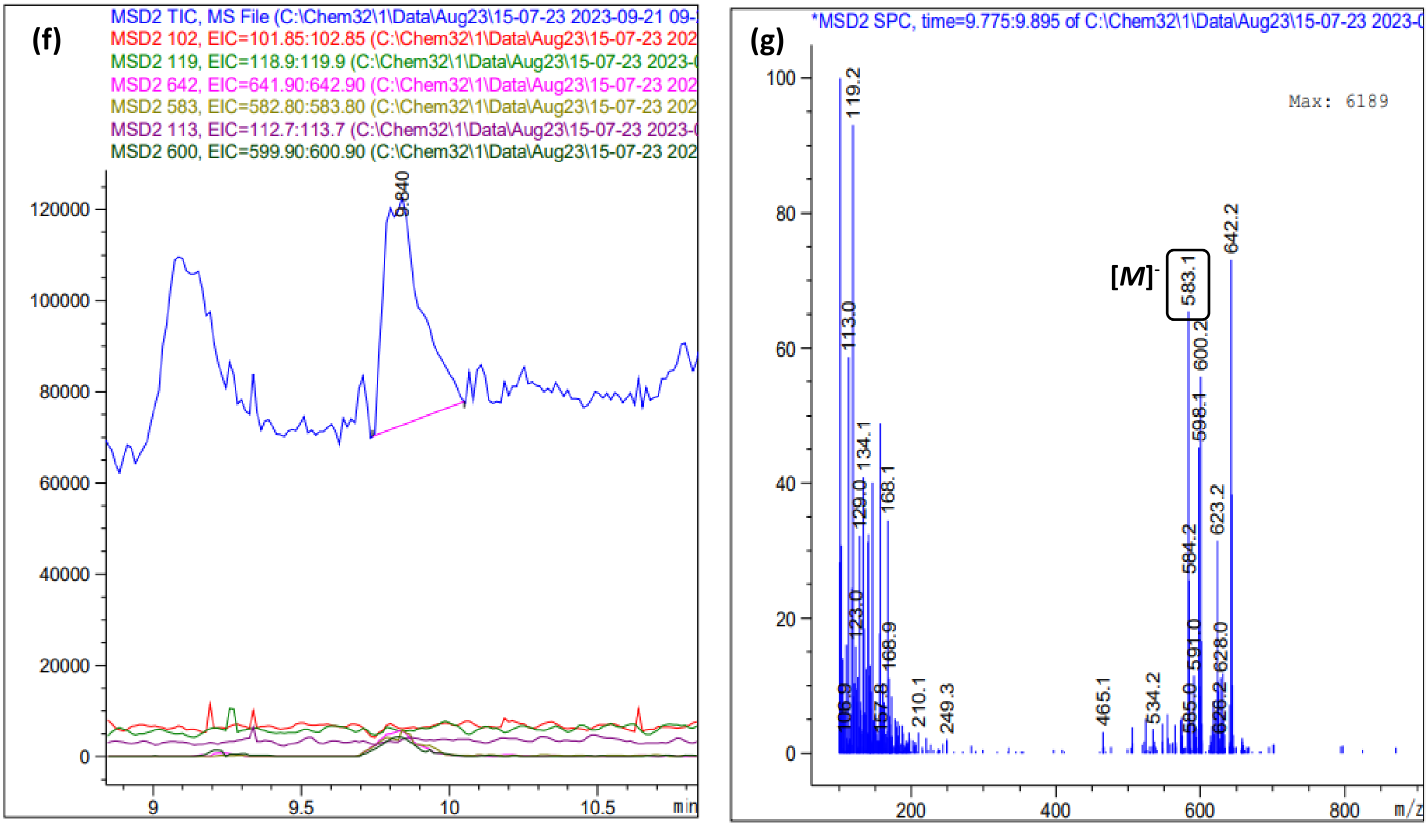

Complex Mixture 2 of Product 46 - (a) Positive MS scan, **(b)** Negative MS scan, **(c)** UV trace, **(d)** Expansion of +ve MS scan, **(e)** MS trace from +ve scan, **(f)** Expansion of -ve MS scan, **(g)** MS trace from -ve scan

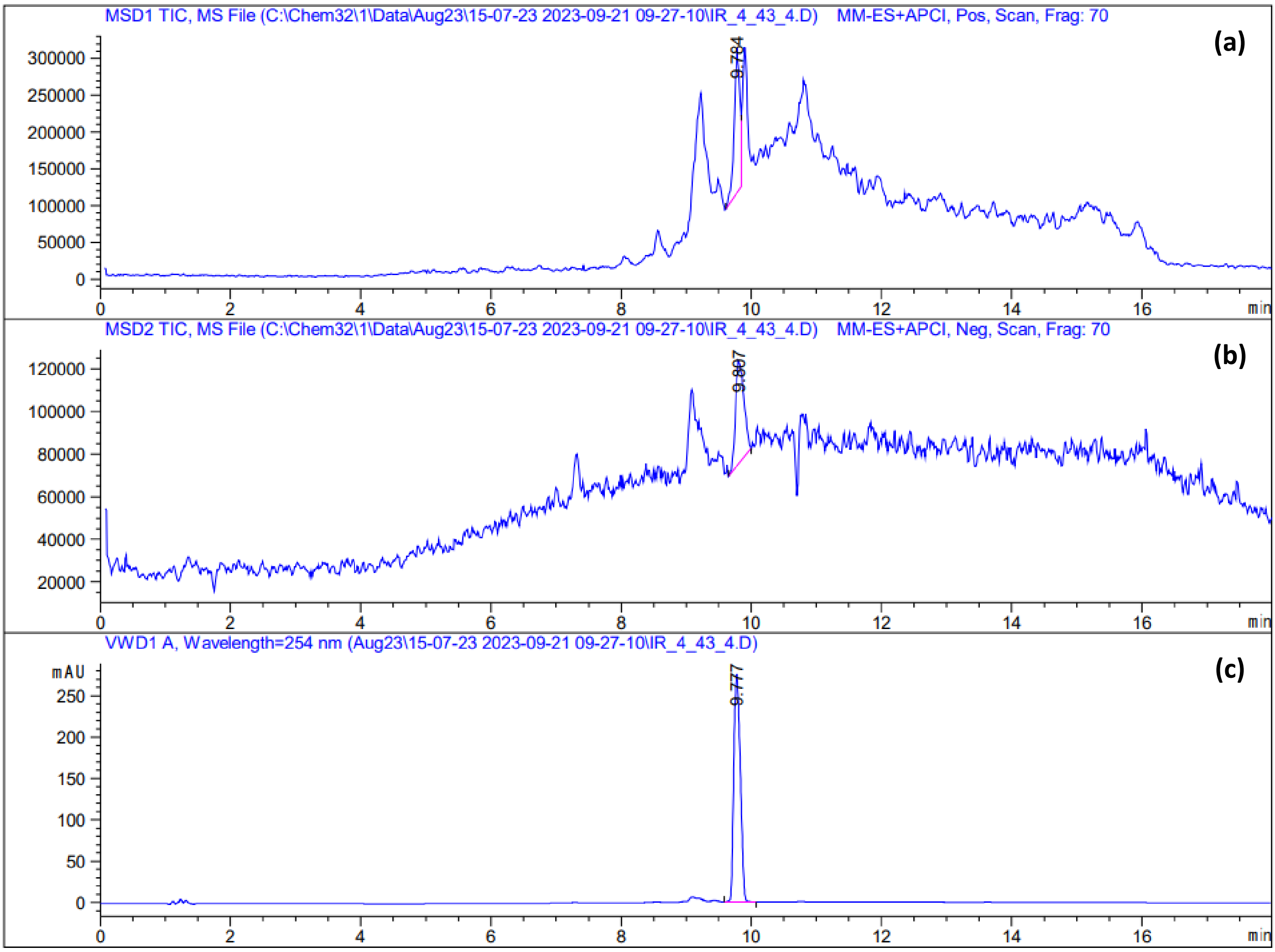

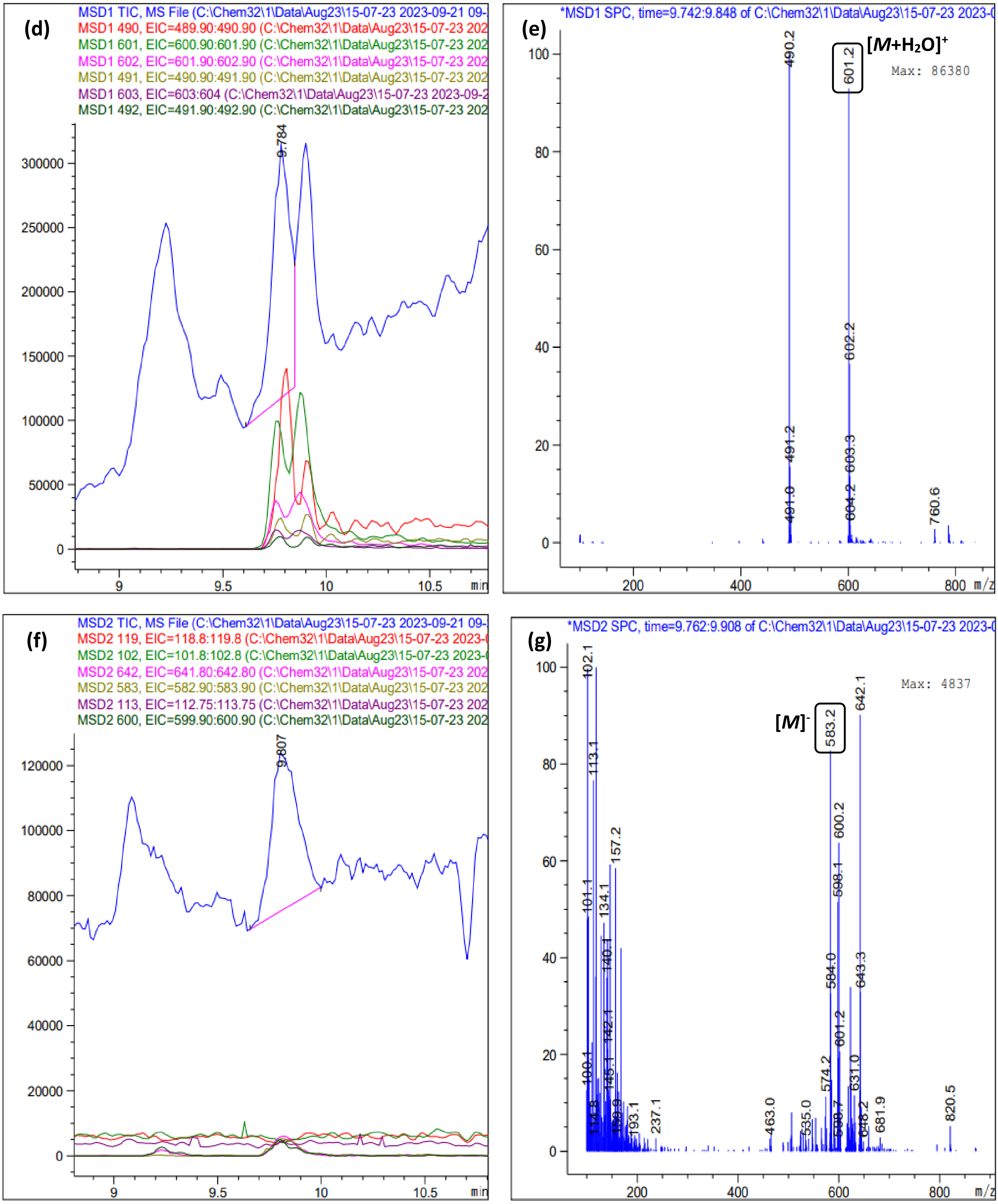
(d)
MSD1 TIC, MS File (C:\Chem32\1\Data\Aug23\15-07-23 2023-09-21 09-
MSD1 490, EIC=489.90:490.90 (C:\Chem32\1\Data\Aug23\15-07-23 202
MSD1 601, EIC=600.90:601.90 (C:\Chem32\1\Data\Aug23\15-07-23 202
MSD1 602, EIC=601.90:602.90 (C:\Chem32\1\Data\Aug23\15-07-23 202
MSD1 491, EIC=490.90:491.90 (C:\Chem32\1\Data\Aug23\15-07-23 202
MSD1 603, EIC=603:604 (C:\Chem32\1\Data\Aug23\15-07-23 2023-09-2
MSD1 492, EIC=491.90:492.90 (C:\Chem32\1\Data\Aug23\15-07-23 202
9.784
min
(e)
*MSD1 SPC, time=9.742:9.848 of C:\Chem32\1\Data\Aug23\15-07-23 2023-0
[M+H2O]+
Max: 86380
490.2
601.2
602.2
491.2
603.3
491.0
604.2
760.6
m/z
(f)
MSD2 TIC, MS File (C:\Chem32\1\Data\Aug23\15-07-23 2023-09-21 09-
MSD2 119, EIC=118.8:119.8 (C:\Chem32\1\Data\Aug23\15-07-23 2023-0
MSD2 102, EIC=101.8:102.8 (C:\Chem32\1\Data\Aug23\15-07-23 2023-0
MSD2 642, EIC=641.80:642.80 (C:\Chem32\1\Data\Aug23\15-07-23 202
MSD2 583, EIC=582.90:583.90 (C:\Chem32\1\Data\Aug23\15-07-23 202
MSD2 113, EIC=112.75:113.75 (C:\Chem32\1\Data\Aug23\15-07-23 202
MSD2 600, EIC=599.90:600.90 (C:\Chem32\1\Data\Aug23\15-07-23 202
9.807
min
(g)
*MSD2 SPC, time=9.762:9.908 of C:\Chem32\1\Data\Aug23\15-07-23 2023-0
Max: 4837
[M]-
583.2
642.1
102.1
113.1
157.2
600.2
101.1
134.1
598.1
140.1
584.0
643.3
142.1
601.2
100.1
145.1
574.2
631.0
114.8
159.9
193.1
237.1
463.0
535.0
681.9
820.5
m/z

Appendix 4: Crude ^{1}H NMR Spectra Used For Calculation of NMR Yields

2-Phenoxypropanenitrile (**16**)

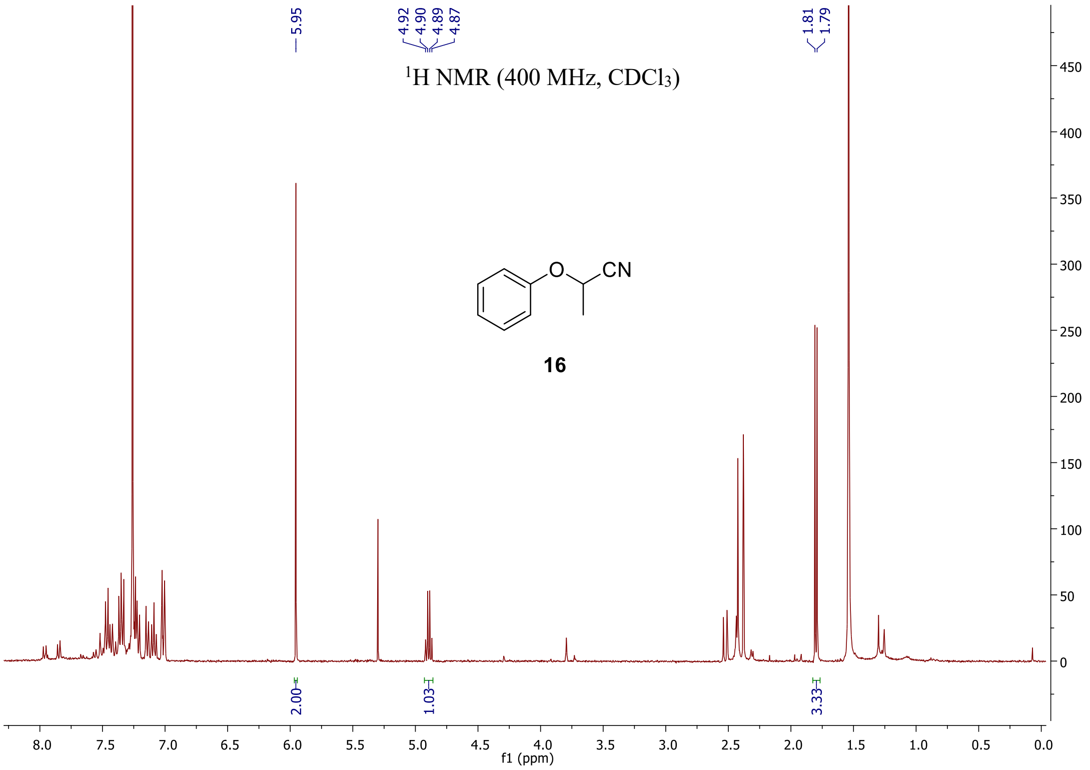

2-(3-Acetylphenoxy)acetonitrile (**26**)

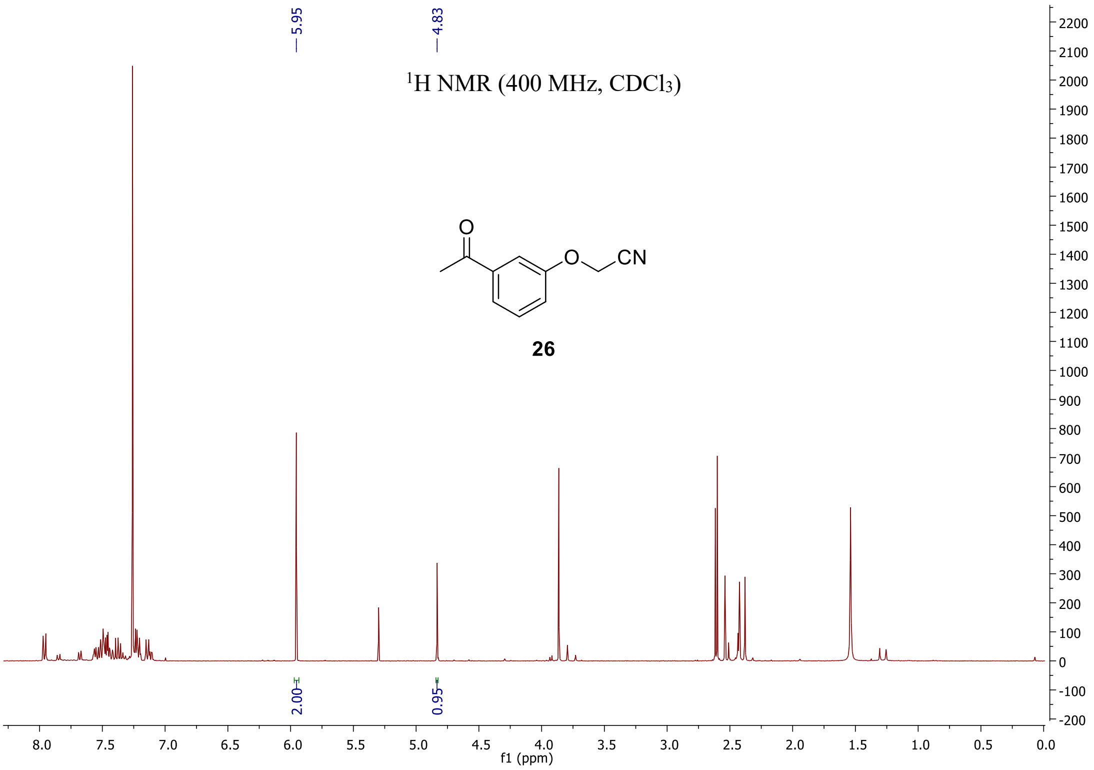

Appendix 5: Example Calculation of NMR Yield (for compound 16)

$$n_{IS} = \frac{m}{MW}$$

$$n_{IS} = \frac{41.6}{167.8}$$

$$n_{IS} = 0.248\,mmol$$

$$\frac{n_P}{n_{IS}} = \frac{(integral\ of\ product\ peak\ \div no.\ of\ protons)}{(integral\ of\ IS\ peak\ \div no.\ of\ protons)}$$

$$\frac{n_P}{n_{IS}} = \frac{(1.03\ \div 1)}{(2.00\ \div 2)}$$

$$\frac{n_P}{n_{IS}} = 1.03$$

$$\therefore\ n_P = 1.03\ \times\ n_{IS}$$

$$n_P = 1.03\ \times 0.248$$

$$n_P = 0.255\,mmol$$

$$NMR\ \%\ Yield = \frac{n_P}{Theoretical\ n} \times 100$$

$$NMR\ \%\ Yield = \frac{0.255}{0.300} \times 100$$

$$\underline{NMR\ \%\ Yield = 85\%}$$

References

1 White, A. R.; Wang, L.; Nicewicz, D. A. Synthesis and Characterization of Acridinium Dyes for Photoredox Catalysis, *Synlett* **2019**, *30*, 827–832.

2 Vechorkin, O.; Hu, X. Nickel-catalyzed cross-coupling of non-activated and functionalized alkyl halides with alkyl grignard reagents, *Angew. Chem. Int. Ed.* **2009**, *48*, 2937–2940.

3 Bell, J. D.; Robb, I.; Murphy, J. A. Highly selective α-aryloxyalkyl C-H functionalisation of aryl alkyl ethers, *Chem. Sci.* **2022**, *13*, 12921–12926.

4 Shan, W. J.; Huang, L.; Zhou, Q.; Meng, F. C.; Li, X. S. Synthesis, biological evaluation of 9-N-substituted berberine derivatives as multi-functional agents of antioxidant, inhibitors of acetylcholinesterase, butyrylcholinesterase and amyloid-β aggregation, *Eur. J. Med. Chem.* **2011**, *46*, 5885–5893.

5 Lever, O.W.; Bell, L. N.; McGuire, H. M.; Ferone, R. Monocyclic pteridine analogs. Inhibition of Escherichia coli dihydropteroate synthase by 6-amino-5-nitrosoisocytosines, *J. Med. Chem.* **1985**, *28*, 1870–1874.

6 Pickford, H. D.; Nugent, J.; Owen, B.; Mousseau, J. J.; Smith, R. C.; Anderson, E. A.; Twofold Radical-Based Synthesis of N , C -Difunctionalized Bicyclo[1.1.1]pentanes, *J. Am. Chem. Soc.* **2021**, *143*, 9729–9736.

7 Kamijo, S.; Kamijo, K.; Maruoka, K.; Murafuji, T. Aryl Ketone Catalyzed Radical Allylation of C(sp^3)–H Bonds under Photoirradiation, *Org. Lett.* **2016**, *18*, 6516–6519.

8 Liu, H.; Ge, L.; Wang, D.; Chen, N.; Feng, C. Photoredox-Coupled F-Nucleophilic Addition: Allylation of gem -Difluoroalkenes, *Angew. Chem. Int. Ed.* **2019**, *58*, 3918–3922.

9 Li, Y.; Zhang, J.; Li, D.; Chen, Y. Metal-Free C(sp^3)–H Allylation via Aryl Carboxyl Radicals Enabled by Donor–Acceptor Complex, *Org. Lett.* **2018**, *20*, 3296–3299.

10 Fuentes De Arriba, A. L.; Urbitsch, F.; Dixon, D. J. Umpolung synthesis of branched α-functionalized amines from imines via photocatalytic three-component reductive coupling reactions, *Chem. Commun.* **2016**, *52*, 14434–14437.

11 Undeela, S.; Thadkapally, S.; Nanubolu, J. B.; Singarapu, K. K.; Menon, R. S. Catalyst-controlled divergence in cycloisomerisation reactions of N-propargyl-N-vinyl sulfonamides: Gold-catalysed synthesis of 2-sulfonylmethyl pyrroles and dihydropyridines, *Chem. Commun.* **2015**, *51*, 13748–13751.

12 GLAXOSMITHKLINE Plc., WO2019/68782, 2019.

13 Zhu, L.; Song, D.; Liu, Y.-H.; Chen, M.-D.;. Zhang, X.-R; You, M.-Y.; Zhan, J.-L. Iron-catalyzed regioselective synthesis of (*E*)-vinyl sulfones mediated by unprotected hydroxylamines, *Org. Biomol. Chem.* **2022**, *20*, 9127–9131.

14 Lou, Y.; Qiu, J.; Yang, K.; Zhang, F.; Wang, C.; Song, Q. Ni-Catalyzed Reductive Allylation of α-Chloroboronates to Access Homoallylic Boronates, *Org. Lett.* **2021**, *23*, 4564–4569.

15 Zhang, J.; Wu, W.; Ji, X.; Cao, S. Perfluorobutyl iodide-assisted direct cyanomethylation of azoles and phenols with acetonitrile, *RSC Adv.* **2015**, *5*, 20562–20565.

16 Barkin, J. L.; Faust, M. D.; Trenkle, W. C. Cytosine Analogues from Substituted Acetonitriles via Thorpe Condensation, *Org. Lett.* **2003**, *5*, 3333–3335.

17 Ramirez, N. P.; Lana-Villarreal, T.; Gonzalez-Gomez, J. C. Direct Decarboxylative Allylation and Arylation of Aliphatic Carboxylic Acids Using Flavin-Mediated Photoredox Catalysis, *Eur. J. Org. Chem.*, **2020**, 1539–1550.
